# Virome in the cloaca of wild and breeding birds revealed a diversity of significant viruses

**DOI:** 10.1186/s40168-022-01246-7

**Published:** 2022-04-12

**Authors:** Tongling Shan, Shixing Yang, Haoning Wang, Hao Wang, Ju Zhang, Ga Gong, Yuqing Xiao, Jie Yang, Xiaolong Wang, Juan Lu, Min Zhao, Zijun Yang, Xiang Lu, Ziyuan Dai, Yumin He, Xu Chen, Rui Zhou, Yuxin Yao, Ning Kong, Jian Zeng, Kalim Ullah, Xiaochun Wang, Quan Shen, Xutao Deng, Jianmin Zhang, Eric Delwart, Guangzhi Tong, Wen Zhang

**Affiliations:** 1 Shanghai Veterinary Research Institute, Chinese Academy of Agricultural Sciences, Shanghai, 200241 China; 2 School of Medicine, Jiangsu University, Zhenjiang, 212003 Jiangsu China; 3 School of Geography and Tourism, Harbin University, Harbin, 150886 Heilongjiang China; 4 Key Laboratory of Wildlife diseases and Biosecurity Management of Heilongjiang Province, Harbin, 150886 Heilongjiang China; 5 Department of Clinical Laboratory, The Affiliated Huai’an Hospital of Xuzhou Medical University, Huai’an, 223002 Jiangsu China; 6 Animal Science College, Tibet Agriculture and Animal Husbandry University, Nyingchi, 860000 Tibet China; 7 Wildlife and Protected Area College/Center of Conservation Medicine and Ecological Safety Northeast Forestry University, Harbin, 150006 Heilongjiang China; 8 Vitalant Research Institute, San Francisco, CA 94118 USA; 9 College of Veterinary Medicine, South China Agricultural University, Guangzhou, 510642 Guangdong China; 10 Department of Laboratory Medicine, University of California San Francisco, San Francisco, CA 94118 USA; 11 Jiangsu Co-Innovation Center for the Prevention and Control of Important Animal Infectious Disease and Zoonose, Yangzhou University, Yangzhou, 225009 Jiangsu China; 12 International Center for Genomics Research, Jiangsu University, Zhenjiang, 212013 Jiangsu China

**Keywords:** Wild bird, Virome, Recombinant, Complete genome, Phylogenetic analysis, Cross-species infection

## Abstract

**Background:**

Wild birds may harbor and transmit viruses that are potentially pathogenic to humans, domestic animals, and other wildlife.

**Results:**

Using the viral metagenomic approach, we investigated the virome of cloacal swab specimens collected from 3182 birds (the majority of them wild species) consisting of > 87 different species in 10 different orders within the Aves classes. The virus diversity in wild birds was higher than that in breeding birds. We acquired 707 viral genomes from 18 defined families and 4 unclassified virus groups, with 265 virus genomes sharing < 60% protein sequence identities with their best matches in GenBank comprising new virus families, genera, or species. RNA viruses containing the conserved RdRp domain with no phylogenetic affinity to currently defined virus families existed in different bird species. Genomes of the astrovirus, picornavirus, coronavirus, calicivirus, parvovirus, circovirus, retrovirus, and adenovirus families which include known avian pathogens were fully characterized. Putative cross-species transmissions were observed with viruses in wild birds showing > 95% amino acid sequence identity to previously reported viruses in domestic poultry. Genomic recombination was observed for some genomes showing discordant phylogenies based on structural and non-structural regions. Mapping the next-generation sequencing (NGS) data respectively against the 707 genomes revealed that these viruses showed distribution pattern differences among birds with different habitats (breeding or wild), orders, and sampling sites but no significant differences between birds with different behavioral features (migratory and resident).

**Conclusions:**

The existence of a highly diverse virome highlights the challenges in elucidating the evolution, etiology, and ecology of viruses in wild birds.

Video Abstract

**Supplementary Information:**

The online version contains supplementary material available at 10.1186/s40168-022-01246-7.

## Background

With changes in ecological environment, climate change, expansion of human activities, and advances in life science and computing technology, novel pathogens are emerging and being found with increasing frequency [1, 2]. In 2020, the severe acute respiratory syndrome coronavirus 2 (SARS-CoV-2) pandemic and its wild animal origin have focused attention on zoonotic infections [3–6]. Wild birds, as important reservoirs for pathogenic viruses, pose a particularly acute threat to human health [7, 8]. Some viruses replicate in the digestive tract of wild birds and are then excreted at high titer in feces [9–11]. In addition to avian influenza virus (AIV), Newcastle disease virus (NDV), fowlpox virus (FPV), and duck plague virus (DPV) can cause severe diseases in domestic poultries. Wild birds also carry Foot and mouth disease virus (FMDV), Japanese encephalitis virus (JEV), West Nile virus (WNV), Borna disease virus, and Eastern equine encephalitis virus [12, 13], which can be pathogenic to humans and other mammals.

Studies have shown that the influenza virus can be isolated in lake water where migratory wild birds gather [14], and avian influenza viral particles can remain infectious for weeks or months in water, food, or sediments [15]. Many wild birds belonging to *Anseriformes* and *Gruiformes* have long migration routes and can migrate between continents. Microorganisms carried by wild birds may mutate and recombine to produce new pathogens, which may spread over a long distance and cause new outbreaks in animals or humans [16–18]. As more novel pathogenic viruses are found in wild birds their roles in spreading viral diseases are coming under increasing investigations. The migration pattern of wild birds, for example, has been suggested to increase the spread of AIV including among domestic poultries [19]. A better understanding of bird migration patterns and of the virome of birds will be useful in helping to predict future outbreaks of emerging zoonotic viruses [12]. Monitoring viruses in wild birds may be used as an early warning system for the incursion of zoonotic viruses. Advances in viral metagenomics prompted us to investigate the enteric virome in wild birds to begin to address such concerns.

When sequencing the human and animal gut virome, majority of the viral nucleotide sequences generally have no significant homology to known viral genomes [20, 21]. Viral metagenomics is a useful technique which combines next-generation sequencing (NGS) and bioinformatics to non-specifically detect both already known and highly divergent viruses. The continuous improvement of NGS makes it possible to massively sequence microbial genomes including viral genomes, powerful bioinformatics tools for identifying viral sequences from NGS big data become increasingly important. Some recent software for identification of viral sequences from metagenomic sequencing data, including the commonly used VirSorter [22], VirFinder [23], and DeepVirFinder [24], were developed, which allow us to effectively identify viruses through their nucleotide or translated protein sequence homologies to all known viruses.

Here we investigated the viral community in cloacal swab collected from 3182 wild and breeding birds to better understand the composition of their enteric viruses and identify potential emerging virus threats to humans or livestock.

## Materials and methods

### Sample collection and preparation

**Fig. 1:**
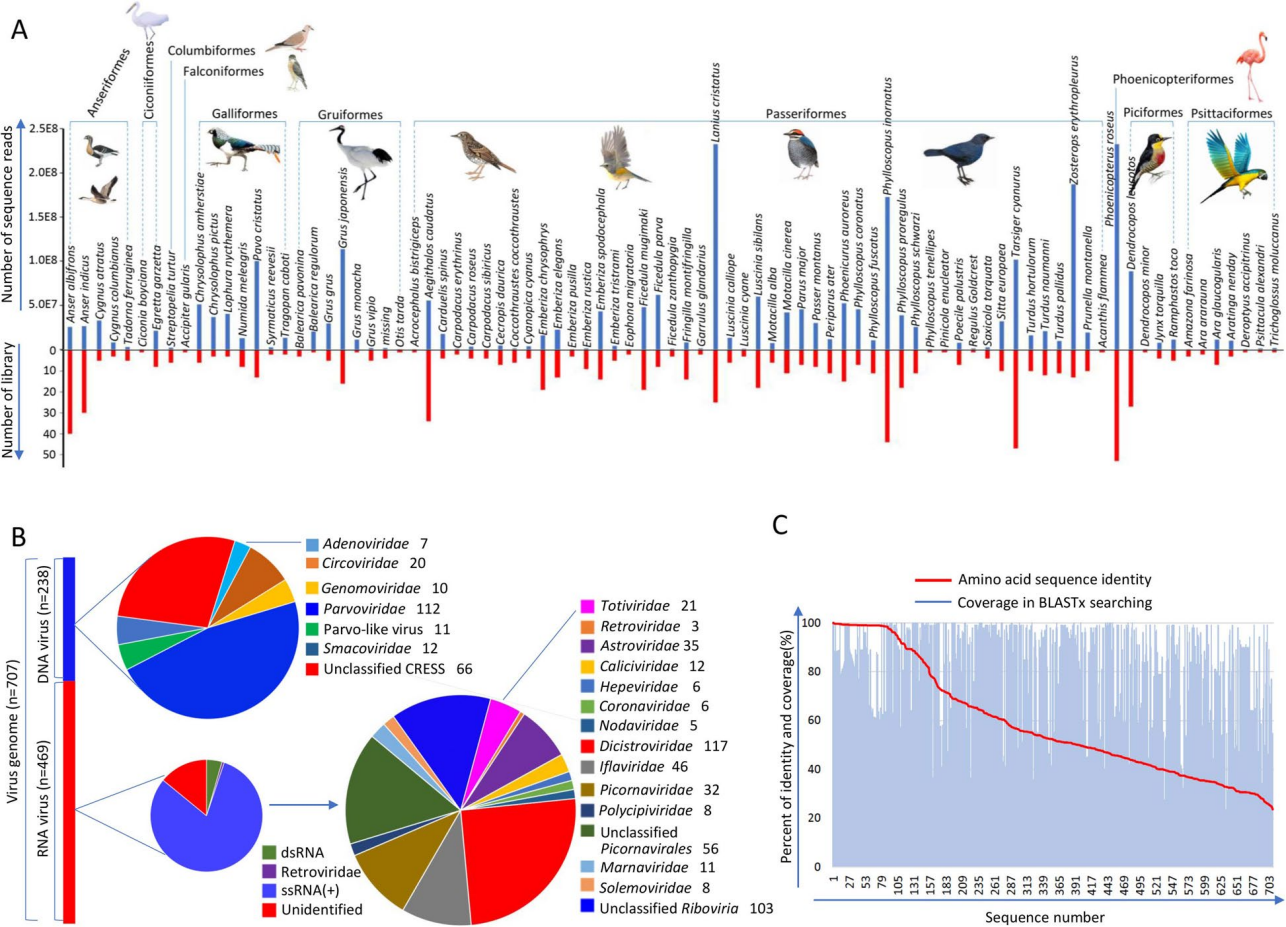
Identification of viruses in different species of birds. **A** Information of bird species and library. The blue bar in the top graph shows the total number of sequence reads in each library corresponding to individual species of bird. The bird species name is shown on top of each bar, while the host Orders are shown above the bar graph. The bottom graph shows the number of libraries for each species of bird in viral metagenomic analysis. **B** The composition and diversity of viruses with complete CDS identified in birds. The left histogram shows the numbers of DNA viruses (blue bar) and RNA viruses (red bar). The right pie charts show the composition of virus types identified in this study. **C** The amino acid sequence identity and coverage of viruses identified here with the best-matched virus strains in BLASTx searching

The goal of this study was to investigate the virome of cloacal swab specimens collected between 2018 and 2019 from 3182 birds in 18 different sampling sites in 8 provinces, China (Fig S1), including > 87 different species of birds belonging to 10 different orders within the Aves class (Fig. 1A and Supplementary Table 1). Among those birds, 2562 were wild and 620 were breeding birds in zoos, farms, or from first-aid centers. All wild birds were captured using cannon nets; cloacal swabs of wild and breeding bird samples were collected by using disposable absorbent cotton swabs and shipped on dry ice. Before viral metagenomic analysis, tips of the swabs were immersed into 0.5 mL of Dulbecco’s phosphate-buffered saline (DPBS) and vigorously vortexed for 5 min and incubated for 30 min at 4 °C. The supernatants were then collected after centrifugation (10 min, 15,000×g) and stored at − 80 °C until use. Most of the birds’ species were identified by experienced field biologists and the uncertain ones were identified by amplification and Sanger sequencing of the cytochrome b gene in the samples. No birds showed any signs of disease. Ethical approvals were given by the Ethics Committee of Chinese Academy of Agricultural Sciences with the reference number of SVRI2017091, the Ethics Committee of Jiangsu University with the reference number of 2018ujs18023, and the Ethics Committee of Key Laboratory of Wildlife Diseases and Biosecurity Management of Heilongjiang Province with the reference number of WDBM2018-023. Sample collecting was performed in accordance with the Wildlife Protection Law of the People’s Republic of China. All samples were shipped to the Shanghai Veterinary Research Institute of Chinese Academy of Agricultural Sciences where sample preparations were conducted in a biosafety level 2 laboratory.

### Viral metagenomic analysis

About 100 μL of the supernatant from each sample was pipetted and pooled into samples pools of the same bird species (details of each pool can be found in Supplementary Table 1). These samples were pooled into a total of 215 sample pools with the average number of 14.8 samples per pool. Sample pools were centrifuged at 12,000g for 20 min at 4 °C to remove eukaryotic and bacterial cell-sized particles and supernatants were filtered through a 0.45-μm filter. Filtrates were then digested by DNase and RNase at 37 °C for 60 min [25–28]. Total nucleic acids were then extracted using QIAamp MinElute Virus Spin Kit (Qiagen) according to the manufacturer’s protocol. Nucleic acid samples were dissolved in DEPC treated water and RNase inhibitors were added. The enriched viral nucleic acid preparations from the respective pools were individually subjected to reverse transcription reactions using reverse transcriptase (Super-Script IV, Invitrogen) and 100 pmol of random hexamer primers, followed by a single round of DNA synthesis using Klenow fragment polymerase (New England BioLabs). A total of 215 libraries were constructed using Nextera XT DNA Sample Preparation Kit (Illumina) and subjected to sequencing on Illumina Miseq or Hiseq platform. For bioinformatics analysis, paired-end reads of 250 bp were debarcoded using vendor software from Illumina. An in-house analysis pipeline running on a 32-node Linux cluster was used to process the data. Reads were considered duplicates if bases 5 to 55 were identical and only one random copy of duplicates was kept. Clonal reads were removed and low sequencing quality tails were trimmed using Phred quality score ten as the threshold. The total read number of each library is shown in Supplementary Table 1. Adaptors were trimmed using the default parameters of VecScreen which is NCBI BLASTn with specialized parameters designed for adapter removal. The cleaned reads were de novo assembled within each barcode using the ENSEMBLE assembler [29]. Contigs and singlets reads were translated in all possible 6 frames in silico and then matched against a customized viral proteome database using BLASTx with an E value cutoff of < 10^−5^, where the virus BLASTx database was compiled using the NCBI virus reference proteome (ftp://ftp.ncbi.nih.gov/refseq/release/viral/) to which was added viral protein sequences from the NCBI nr fasta file (based on annotation taxonomy in Virus Kingdom). Candidate viral hits are then compared to an in-house non-virus non-redundant (NVNR) protein database to remove false-positive viral hits, where the NVNR database was compiled using non-viral protein sequences extracted from the NCBI nr fasta file (based on annotation taxonomy excluding Virus Kingdom). Contigs without a significant BLASTx similarity to the viral proteome database are searched against viral protein families in the vFam database [30] using HMMER3 to detect remote viral protein similarities [31–33].

### Confirmation and extension of virus genomes

Viral contigs that may be from the same genome but without overlap were merged using the software Geneious v11.1.2 and PCR primers bridging gaps were designed [34]. Gaps were filled by (RT–)PCR and Sanger sequencing. To confirm the assembly results of a full genome, reads were de novo assembled back to the full-length genome using the low sensitivity/fastest parameter in Geneious 11.1.2, where the number of sequence reads mapped against each target viral genome was analyzed [34] and shown in the column “Virus Reads” in Supplementary Table 2. For genomes with novel structures, we verified the complete or near-complete coding sequence (CDS) of the viral genome by designing overlapping PCR primers based on the assembled sequences followed by amplicon Sanger sequencing.

### Analyses of virus diversity and distribution

In order to investigate the diversity and distribution of viruses in birds’ cloacal swab samples, we conducted two types of analyses. First, all the reads with sequence length > 50 bp in the clean data were compared to the viral proteome database using BLASTx as mentioned above and the BLASTx results were then loaded into the MEGAN program [35]; rarefaction curves were then conducted to visualize differences in the virus community composition. Second, using the function of “Map to Reference” in the software Geneious v11.1.2, the clean data of NGS from the 215 libraries were respectively aligned to each of the 707 viral genomes which were used as reference genomes, the libraries with > 10 different reads uniformly matched to the reference genome were considered positive. Numbers of each library’s sequence reads mapped to the 707 genomes are shown in Supplementary Table 3 that is further used for the establishment of heat maps of virus distribution.

### Phylogenetic analysis of viruses

To infer phylogenetic relationships, protein sequences of reference strains belonging to different groups of viruses were downloaded from the NCBI GenBank database. Related protein sequences were aligned using the alignment program implemented in the CLC Genomics Workbench 10.0, and the resulting alignment was further optimized using MUSCLE in MEGA v7.0 [36] and MAFFT v7.3.1 employing the E-INS-I algorithm [37]. Sites containing more than 50% gaps were temporarily removed from alignments. Bayesian inference trees were then constructed using MrBayes v3.2 [38]. During MrBayes analysis, we used “lset nst=6 rates=invgamma” for phylogenetic analysis based on nucleotide sequences of *Deltacoronavirus*, which set the evolutionary model to the GTR substitution model with gamma-distributed rate variation across sites and a proportion of invariable sites ("GTR+I+Г"), while we set “prset aamodelpr=mixed” for the phylogenetic analysis using amino acid sequences, which allows the program to utilize the 10 built-in amino acid models. The number of generations was increased to a maximum of 10 million until the standard deviation of split frequencies is below 0.01, in which every 50 generations were sampled and the first 25% of Markov chain Monte Carlo (mcmc) samples were discarded as burn-in. Maximum likelihood trees were also constructed to confirm all the Bayesian inference trees using software Mega v7.0 [36].

### Virus genome annotation

Putative viral open reading frames (ORFs) were predicted by Geneious v11.1.2 with built-in parameters (minimum size: 300; genetic code: standard; start codons: ATG )[34], further were checked through comparing to related viruses by Blastp in NCBI. The annotations of these ORFs were based on comparisons to the Conserved Domain Database. Potential exon and intron of some viruses (e.g., viruses showing similarity to the family *Genomoviridae*) were predicted by Netgenes2 at http://www.cbs.dtu.dk/services/NetGene2/. Sequence similarity scanning among related virus genomes was performed using the Simplot software version 3.5.1. International Committee on Taxonomy of Viruses (ICTV) criteria (https://talk.ictvonline.org/ictv-reports/ictv_online_report/) used to genetically and phylogenetically characterize each of the putative new genus or species of viruses described in this study are listed in Supplementary Table 4.

### Quality control in library construction and virus detection

To test laboratory environment and reagent contamination, 5 swab samples were collected from the surface of laboratory worktops, instruments, reagent bottle, and pipettes using DPBS-soaked swabs. Then, the cotton tips of swab samples were re-suspended in 0.5 mL of DPBS and vigorously vortexed for 5 min, following frozen and thawed three times on dry ice. The supernatants were then collected after centrifugation (10 min, 15,000 g) and subjected to viral metagenomic analysis as those of birds’ cloacal swab samples. The Illumina Miseq sequencing data of the control samples were deposited into the GenBank SRA database with accession no. SRX11518259, SRX11518260, SRX11518262, SRX11518284, and SRX11518414.

Finding a large number of unclassified *Riboviria* and unclassified CRESS DNA virus, conventional PCR was conducted with primers designed based on 20 randomly selected genomes of these two groups of viruses to test whether these viruses were present in the original cloacal swab samples, where the PCR templates were nucleic acid extracted from the original sample pools using Trizol reagents. The positive PCR products were further confirmed by Sanger sequencing. Information of the 20 genomes and primers used in the PCR reactions are provided in Supplementary Table 5.

## Results

### Overall view of the virome

We performed a large-scale viral metagenomics survey of potential viruses in 3182 cloacal swab specimens which were collected from 620 breeding birds in zoos or farms and 2562 wild birds in natural reserves or parks, respectively, located in 18 different sampling sites in 8 different provinces in China (Fig. 1A, Supplementary Table 1, and Supplementary Fig. 1). In total, the 215 libraries generated 480,609,464 sequence reads. The assembled sequence contigs and showing significant similarity to known viruses were further analyzed. From these data, 707 different complete or nearly complete CDS of virus genomes were determined by using sequence assembly combined with PCR and Sanger sequencing of gaps between contigs. These 707 genomes belong to 23 different groups of viruses including 19 defined families and 4 unclassified virus groups (Fig. 1B and Supplementary Table 2). A total of 572 viral genomes were from wild birds and 135 genomes were from breeding birds. A total of 469 genomes belonged to RNA viruses and 238 belonged to DNA viruses (Fig. 1B). BLASTx search results for these 707 genomes revealed that 265 genomes shared < 60% amino acid sequence identities with their best matches in GenBank (Fig. 1C and Supplementary Table 2), suggesting these virus genomes could be considered novel viruses, forming putative new virus families, genera, or species. To phylogenetically analyze these viruses, the protein sequence of their most conserved regions, including their RNA-dependent RNA polymerase (RdRp) domains for virus belonging to *Riboviria*, replication proteins (Rep) for CRESS DNA virus, and non-structural protein (NS) for parvoviruses and adenovirus were used in phylogenetic analysis.

The vial metagenomic analysis results indicated although a small number of viral reads showing sequence similarity to human or plant viruses (including anellovirus, papillomavirus, and herpesvirus which may come from human skin and geminivirus and partitivirus which may come from pl potted plant in our laboratory), most of the viral reads in the environmental samples belong to phages hosted by prokaryotic organisms (Supplementary Fig. 2).

PCR confirmation and Sanger sequencing of the 20 randomly selected genomes of unclassified CRESS DNA virus or unclassified Riboviria confirmed the real existence of these viruses in the original bird cloacal samples.

### Virus diversity and distribution in cloacal swab of birds

**Fig. 2:**
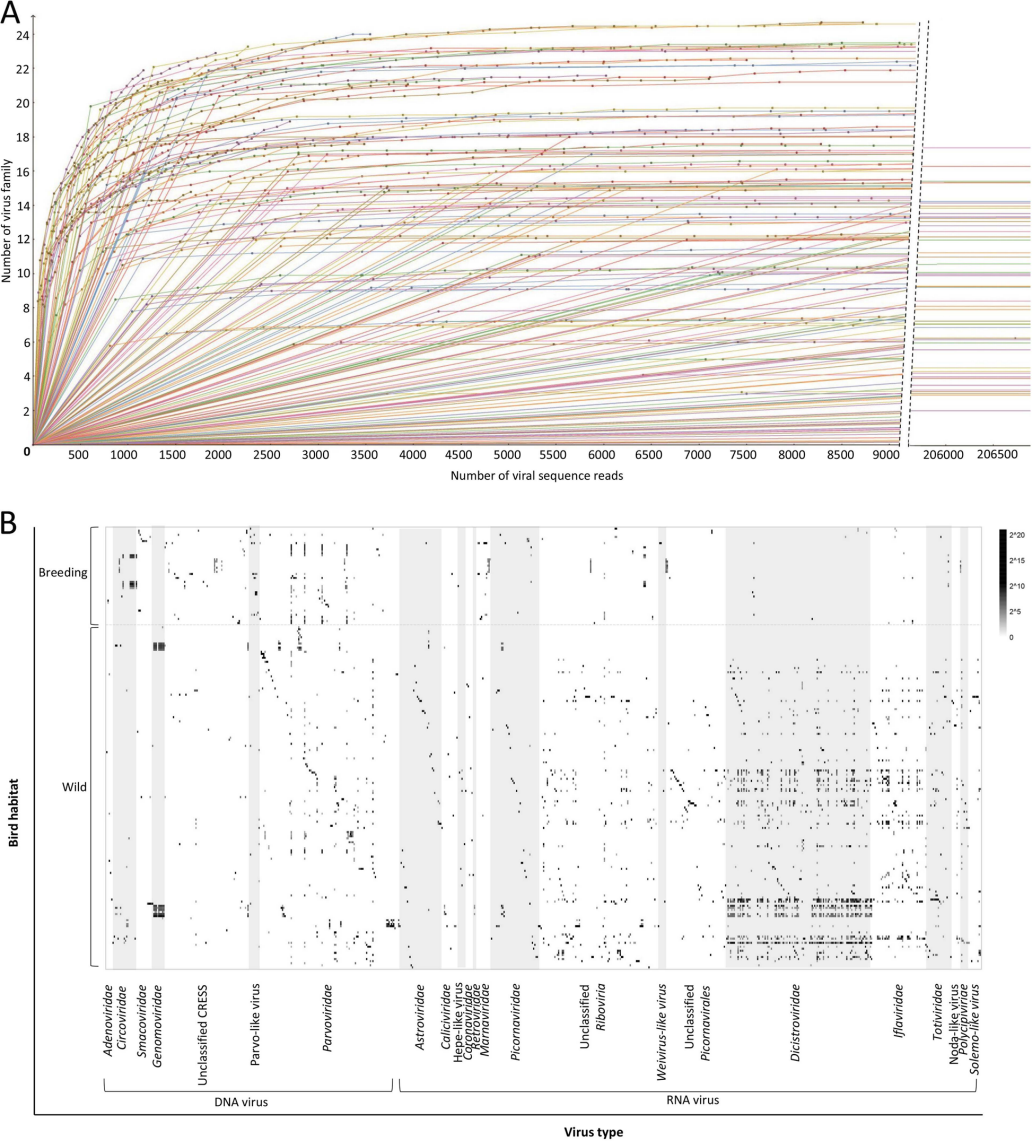
Rarefaction curves of observed virus family and virus distribution pattern in the 215 birds’ cloacal swab pools based on birds’ habitat. **A** In the rarefaction curves the horizontal ordinate represents the number of viral sequence reads obtained from the NGS data (where the “break” axis is used because some libraries contain too large a number of viral reads), the longitudinal axis represents the number of virus families observed in the virome of birds’ cloaca swab samples. **B** The horizontal ordinate represents different virus genomes which are further divided into 24 different virus families or groups, while the longitudinal axis represents the 215 libraries that are arranged based on birds’ habitat (breeding or wild). Heatmap representing the read number (in exponential form) in the mapping analysis using the 707 genomes against NGS data of the 215 libraries (see color legend)

Here, rarefaction curves were used to visualize virus family composition in the cloacal swab of birds (Fig. 2A), which showed that the number of viral sequence reads in each library ranged from 897 (library ID: parrot87) to 28,260,000 (library ID: siskin57), and in most of the 215 libraries, the observed virus families showed stable when the viral sequences number reached to a certain number, suggesting that the sequencing depths in most the 215 libraries were sufficient to represent the presence of virus families in the birds’ fecal samples and the sequencing data were rational and cogent. The rarefaction curves also showed that the number of virus families in different libraries varied from 1 to 25. The mapping analysis using the 707 genomes against the 215 NGS data revealed the virus distribution in the 215 sample pools, where the distribution patterns were further analyzed based on birds’ habitat (breeding or wild) (Fig. 2B), orders (Supplementary Fig. 3), behavioral feature (migratory and resident) (Supplementary Fig. 4), and sampling sites (Supplementary Fig. 5).

On the whole, the virus diversity in wild birds (birds living in a natural or undomesticated state) is higher than that in breeding birds (Fig. 2B). Some viruses showed different distribution patterns between breeding and wild birds. The prevalences of *Dicistroviridae* and *Iflaviridae* in wild birds are much higher than those in breeding birds. Over the bird order level, the virus diversity in birds belonging to *Passeriformes* is slightly higher than those in birds belonging to the other 9 orders (Supplementary Fig. 3). The virus diversities between migratory birds (birds that travel from one place to another at regular times often over long distances in order to breed, feed, and raise their offspring) and resident birds showed no evident difference (Supplementary Fig. 4). Comparing the virus diversity in different sampling sites indicated that the fecal samples from Changbaishan Mountain and MES Mountain had higher diversity than those from other sampling sites (Supplementary Fig. 5). The distribution pattern of different types of viruses showed differences according to birds’ diet and behavioral features. For example, reflecting the diet of birds, the *Dicistroviridae* and *Iflaviridae* were wildly present in the wild birds. Viruses belonging to unclassified *Riboviria* and *Picornavirales* showed distributions different from those of *Dicistroviridae* and *Iflaviridae* but slightly similar to those of viruses belong to *Picornaviridae*, *Caliciviridae*, and *Totiviridae* (Fig. 2B and Supplementary Figs. 3, 4, 5).

### Novel RNA viruses identified in cloaca of birds

We identified 469 RNA virus genomes in cloaca swab of birds belonging to 15 virus families or unclassified groups. Potential avian viruses showing similarity to *Astroviridae* (*n* = 35), *Picornaviridae* (*n* = 32), *Caliciviridae* (*n* = 12), *Hepeviridae* (*n* = 6), *Coronaviridae* (*n* = 6), and *Retroviridae* (*n* = 3) were discovered in these samples (Fig. 1B). Other RNA viruses with no prior evidence of infecting birds were also identified including *Dicistroviridae* (*n* = 117), *Iflaviridae* (*n* = 46), *Totiviridae* (*n* = 21), *Marnaviridae* (*n* = 11), *Polycipiviridae* (*n* = 8), *Solemoviridae* (*n* = 8), *Nodaviridae* (*n* = 5), unclassified *Picornavirales* (*n* = 56), and unclassified *Riboviria* (*n* = 103). Viruses belonging to the family of *Dicistroviridae*, *Iflaviridae*, *Totiviridae*, *Marnaviridae*, *Polycipiviridae*, *Solemoviridae*, and *Nodaviridae* are known to infect plants, fungi, insects, or parasites and thus possibly originate from the diet of birds. It is conceivable that some viruses showing similarity to unclassified *Picornavirales* and unclassified *Riboviria* may contain some members infecting birds.

**Fig. 3:**
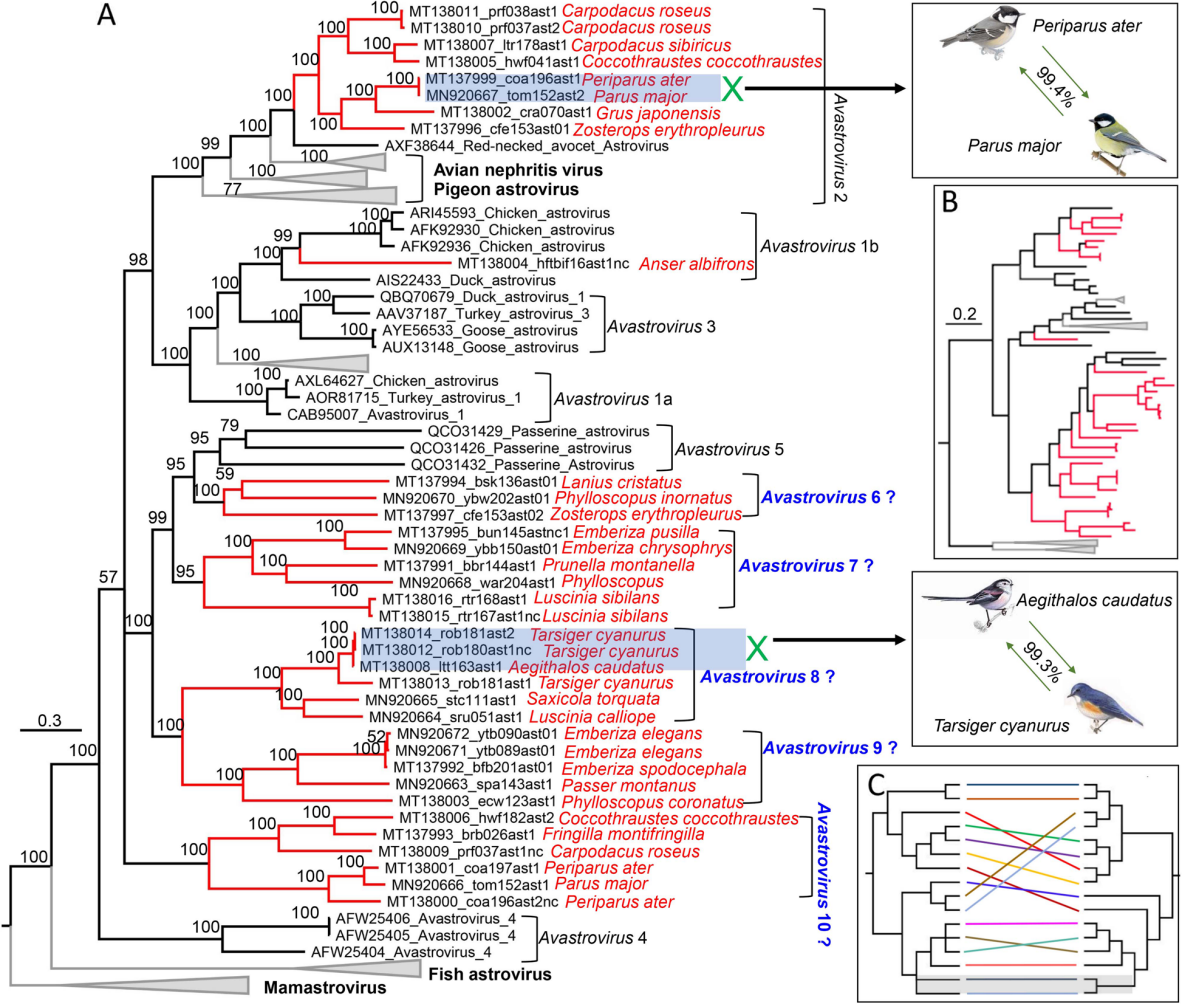
Phylogenies of astroviruses identified in the cloaca of birds. (**A**), (**B**) Bayesian inference trees were constructed using MrBayes v3.2 respectively based on amino acid sequences of Capsid protein (**A**) RdRp (B) of astroviruses, within trees the viruses found in this study are marked with a red line. Species names of birds are indicated. Each scale bar indicates the amino acid substitutions per site. Putative cross-species infections of astrovirus between different species of birds are shown. Putative new species name within the genus *Avastrovirus* is labeled beside the corresponding clade. (**C**) Putative recombination is presented by trees based RdRp (left tree) and capsid protein (right tree) of astroviruses obtained from birds in the present study

Thirty-five astrovirus genomes were characterized from 23 different species of birds (Fig. 3A). Phylogenetic trees based on capsid (Fig. 3A) and RdRp protein sequences (Fig. 3B) indicated that these astroviruses are grouped into 7 different clades in the genus *Avastrovirus*. The classification of astroviruses has been redefined several times since they were first discovered in 1975. According to ICTV, the current classification does not correspond to the phylogeny of this group of viruses, being based on the host and the genetic distances (*p*-distance) among complete amino acid sequences of the capsid region (ORF2). The *Astroviridae* study group of ICTV also establishes that viruses with a *p*-distance > 75% identity in the complete protein sequence of ORF2 should be considered members of the same species [39]. In the clade of *Avastrovirus* 2, 8 novel astroviruses were from 6 different species of birds and clustered together, sharing < 65% sequence similarity with other members of *Avastrovirus* 2 based on the amino acid sequence of capsid and RdRp. An astrovirus (MT138004) from white-fronted goose (*Anser albifrons*) formed a single branch within the clade of *Avastrovirus* 1 (Fig. 3A) and shared < 70% similarity with the RdRp and capsid of other duck and chicken astroviruses, belonging to defined species of *Avastrovirus* 1b. The remaining 26 novel astroviruses showed no close relationship with any defined species and were grouped into 5 distinct clusters, forming 5 putative new different species (i.e., *Avastrovirus* 6–10) within genus *Avastrovirus*. Although the phylogenetic analysis based on RdRp overall showed a similar topological structure to that of capsid (Fig. 3B and Supplementary Fig. 6A), some strains showed discordant positions in the tree over RdRp and capsid protein (Fig. 3C and Supplementary Fig. 6B), suggesting genomic recombination had occurred (i.e., MT138004, MT137991, MT138003, and MT137998). Sequence analysis indicated that the same astrovirus could be discovered in different species of birds as 2 astroviruses (MT138008 from long-tailed tit and MT138012 from red-flanked bush robin) shared 99.3% capsid sequence identity and another 2 astroviruses (MT137999 from coal tit and MN920667 from tomtit) shared 99.4% sequence identity (Fig. 3A), indicating likely cross-species transmissions.

**Fig. 4:**
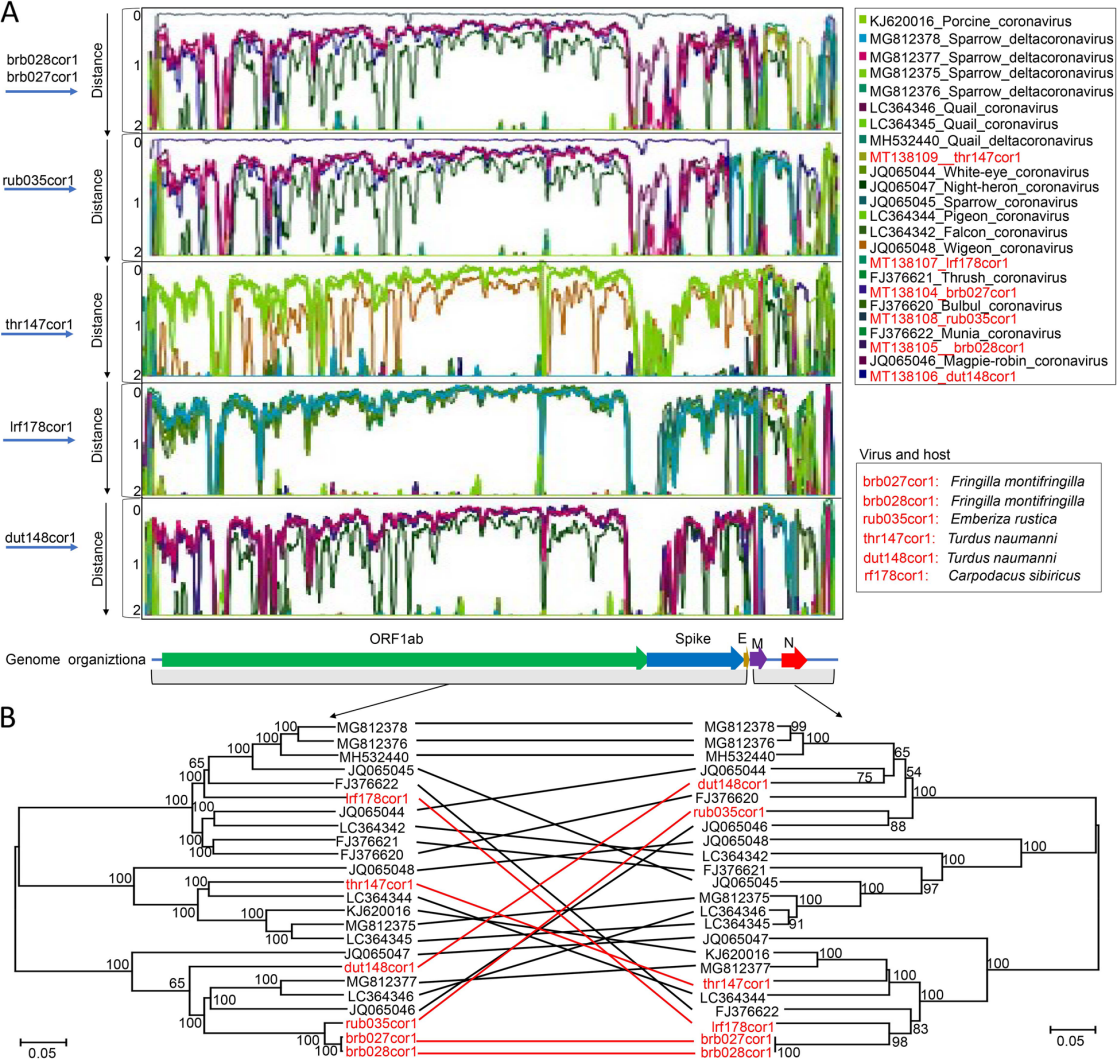
Sequence similarity scanning and recombination analysis of *deltacoronaviruses* from the cloaca of birds. **A** Sequence similarity scanning of the 6 coronavirus genomes against the related coronavirus genomes within *Deltacoronavirus* genus using Simplot software version 3.5.1. Different strains are presented by lines in different colors which are shown on the right side. **B** Bayesian inference trees based on putative exchanged genomic regions. The stain names of genomes acquired here are marked with red color. Each scale bar indicates the amino acid substitutions per site

We characterized 6 coronavirus genomes from 4 species of birds belonging to the *Passeriformes* including one from long-tailed rosefinch, one from rustic bunting, two from dusky thrush, and two from brambling. Based on BLASTn searches, all 6 coronaviruses matched best with *Deltacoronavirus*, sharing < 75% sequence identities with them. Phylogenetic analysis based on the complete CDS of the 6 coronaviruses and those related representative *Deltacoronavirus* genomes from GenBank indicated that they clustered into three different groups (Supplementary Fig. 7). According to the ICTV criteria, coronaviruses that share > 90% aa sequence identity in the conserved replicase domains are considered to belong to the same species; the 6 coronaviruses identified here are qualified to be 3 new species in the *Deltacoronavirus* genus. Sequence similarity plots using 6 genomes identified here against the other related genomes revealed that sequences right downstream of the ORF1ab region of these coronaviruses are hypervariable regions and homologous exchange within genomes occurred in the membrane (M) and nucleocapsid (N) regions (Fig. 4A). Phylogenetic trees based on nucleotide sequence 5′ from M and including M plus downstream regions revealed discordant trees, indicating that 4 of the 6 coronavirus genomes acquired in this study (with the exception of brb028cor1 (MT138105) and brb027cor1 (MT138104) appear to be recombinants (Fig. 4B).

**Fig. 5:**
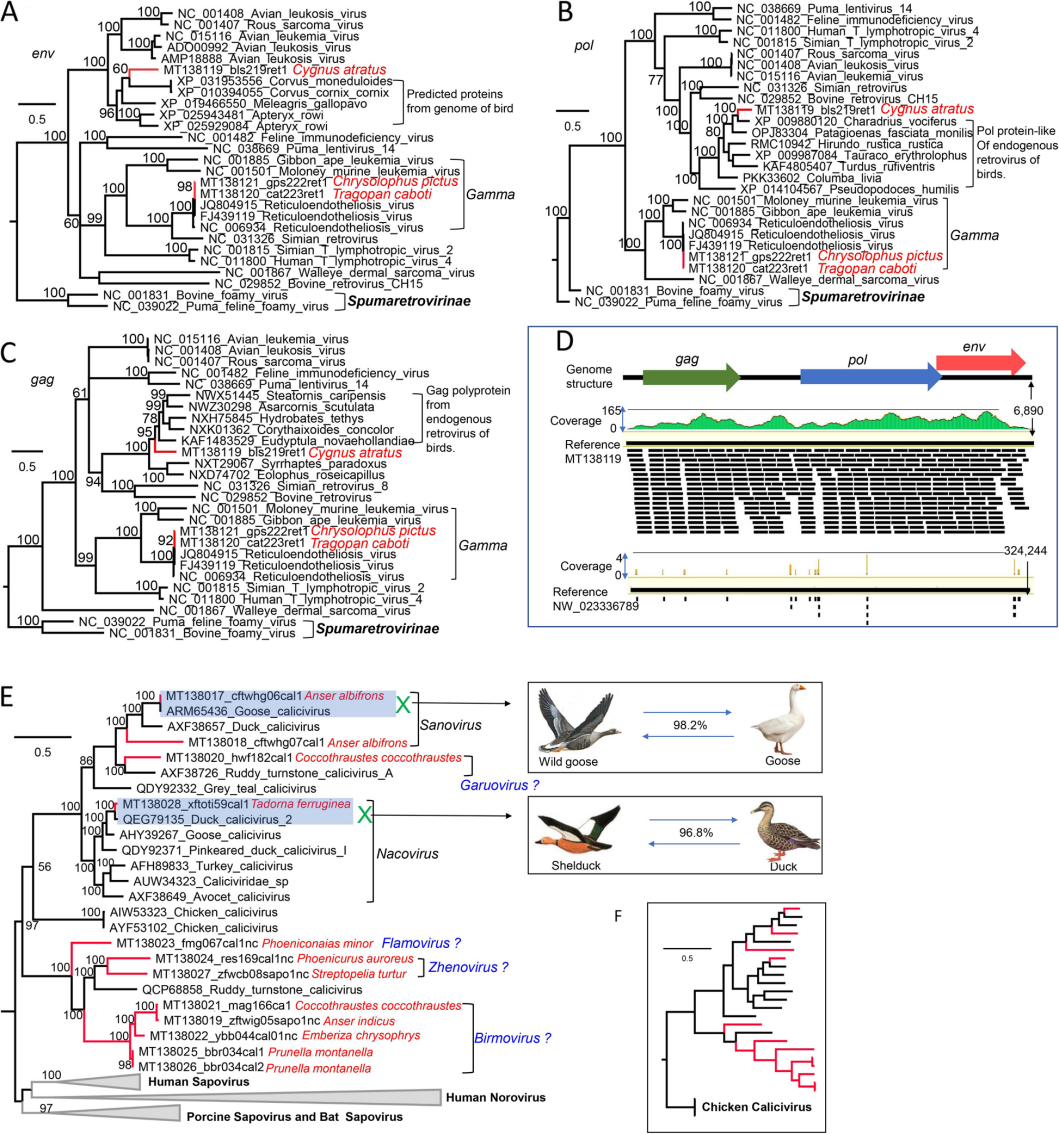
Phylogenies of retroviruses and caliciviruses identified in the cloaca of birds. **A** Bayesian inference tree established based on amino acid sequences of env of retroviruses. **B** Bayesian inference tree established based on amino acid sequences of *pol* of retroviruses. **C** Bayesian inference tree established based on amino acid sequences of *gag* of retroviruses. **D**The top graph shows the genome organization of the new retrovirus identified in black swan (*Cygnus atratus*), the middle graph shows the coverage of sequence reads in library mapping against the genome of this retrovirus, and the bottom graph shows the sequence reads in library mapping against a representative genomic fragment of black swan. **E** Bayesian inference tree established based on amino acid sequences of RdRp of caliciviruses. Putative cross-species infection of calicivirus between wild birds and domestic poultries are shown. Putative new genus name is labeled beside the corresponding clade. **F** Bayesian inference tree established based on amino acid sequences of the capsid of caliciviruses. Within trees, the viruses found in this study are marked with a red line. Species names of birds are indicated. Each scale bar indicates the amino acid substitutions per site

Three genomes belonging to *Retroviridae* were acquired from 3 different species of birds named wild black swan, breeding golden pheasant, and yellow-bellied tragopan (Fig. 5A). Two of the retrovirus genomes belonged in the *Gammaretrovirus* genus showing a close relationship to reticuloendotheliosis viruses previously identified from domestic ducks and geese (Fig. 5A, B, and C), sharing > 99% whole-genome sequence identities with them. The remaining retrovirus (MT138119) presented the typical genome organization of retrovirus with Gag, Pol, and Env ORFs (Fig. 5D). The genome sequence of this novel retrovirus was divergent from all known retroviruses encoding 3 proteins (i.e., Gag, Pol, and Env) with sequence similarity of about 40% to the proteins of endogenous retrovirus predicted from the birds’ genomes (Fig. 5A, B, and C), which makes us think that this retrovirus sequence may be from the endogenous retrovirus sequence integrated into the host genome. However, a comparison of the coverage in mapping analysis using this novel retrovirus genome and 3 genomic fragments of black swan (represented by a genomic scaffold of the *Cygnus atratus* isolate AKBS03, GenBank no. NW_023336789) against the total reads of this library indicated the mapping coverage of this retrovirus genome is significantly higher (with mean coverage = 74.8) than that of the host genome (with mean coverage = 0.006) (Fig. 5D). Considering that we have used DNase and RNase to digest the un-encapsidated host nucleic acids and the genome mapping against the total reads have not produced edged sequence showing similarity to birds’ genome, this retrovirus genome recovered from this library is most likely from virus particle instead of endogenous retrovirus sequence integrated into the genome of birds. Based on the phylogenetic analysis over the 3 encoding proteins, this novel retrovirus may be a new genus within the subfamily *Orthoretrovirinae* (Fig. 5A, B, and C)*.*

Twelve calicivirus genomes were characterized from 9 different species of birds, based on phylogenies of RdRp and capsid proteins 4 of which clustered closely with several known goose or duck caliciviruses while the remaining 8 genomes together with their best BLASTx match, a ruddy turnstone calicivirus, formed a separate clade genetically distinct from the other avian caliciviruses (Fig. 5E and F). Except for 3 genomes which can be grouped into known defined genera, the remaining 9 viruses are qualified to be 4 different putative new genera (temporarily named *Garuovirus*, *Flamovirus*, *Zhenovirus*, and *Birmovirus*) based on the ICTV criteria [40] and phylogenetic analysis over the 3 encoding proteins, where 2 of them also include previously unclassified caliciviruses (Fig. 5E). The phylogenetic trees respectively based on RdRp and capsid showed consistent topological structure suggesting no putative recombination occurred involving these caliciviruses. Sequence analysis indicated that 2 caliciviruses from wild goose and breeding shelduck shared high sequence similarity with caliciviruses from domestic goose and duck respectively, sharing > 96% identities in RdRp (Fig. 5 E).

**Fig. 6:**
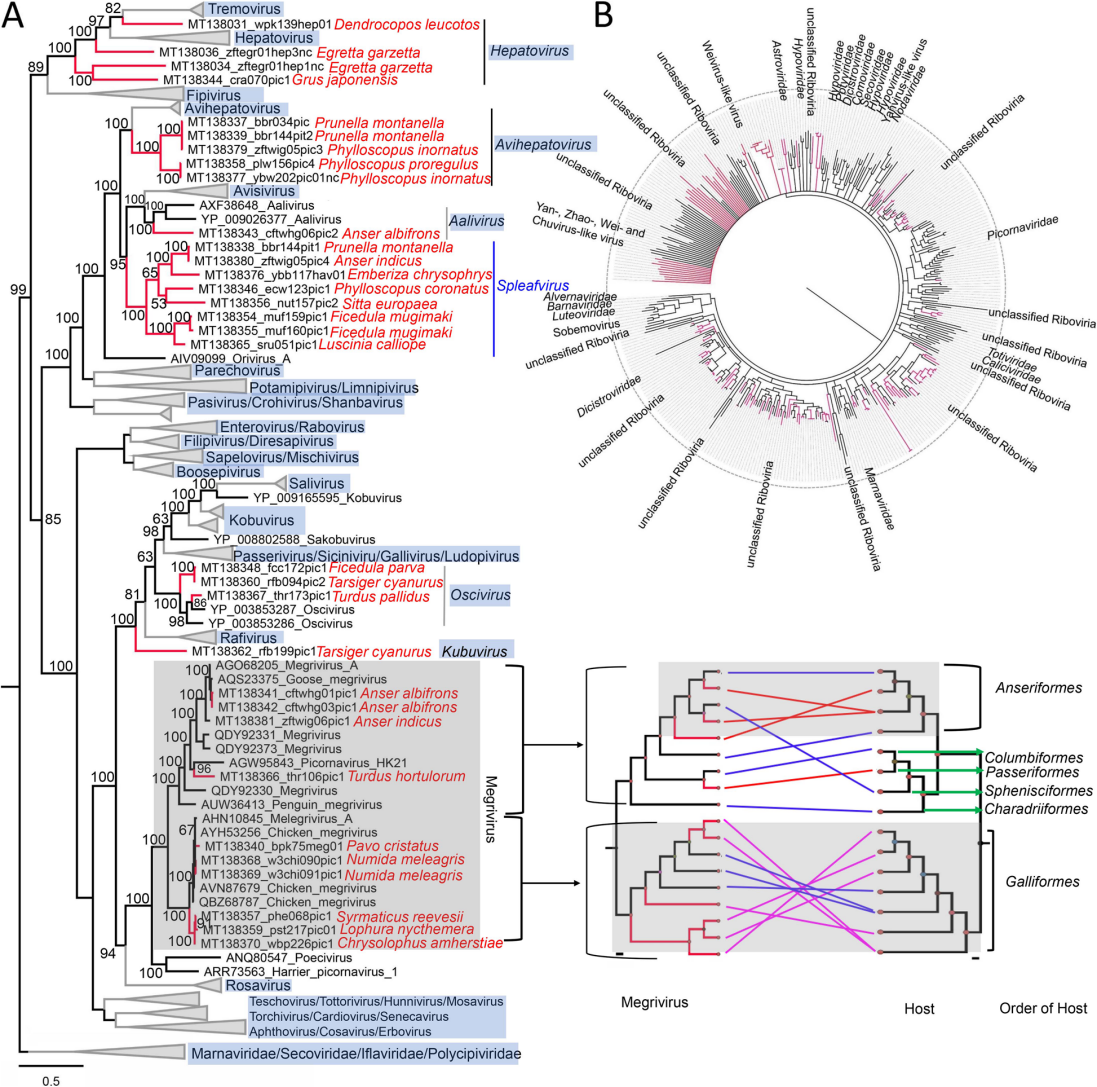
Phylogenies of viruses belonging to the family *Picornaviridae* and unclassified *Riboviria* identified in the cloaca of birds. **A** Bayesian inference tree based on amino acid sequences of RdRp of viruses belonging to the family *Picornaviridae*. Representative strains of all genera in the family *Picornaviridae* are included. Species names of birds are indicated. Comparisons of *Megrivirus* phylogeny based on RdRp and their corresponding host phylogeny are also shown beside the big tree. **B** Bayesian inference tree based on amino acid sequences of RdRp of viruses belonging to unclassified *Picornavirales* and *Riboviria* identified here. Within trees, the viruses found in this study are marked with red line. Each scale bar indicates the amino acid substitutions per site

The family of *Picornaviridae* comprises 63 genera containing 147 species, but many viruses are presently awaiting classification. A novel virus genus may be proposed according to the ICTV criteria which include (i) showing distinctive genome organization features in comparison to their closest relatives, (ii) sharing amino acid sequence identity of < 34 % in P1 and < 36 % amino acid sequence identity in the non-structural proteins 2C+3CD, and (iii) no detectable homology of proteins L (if exist), 2B, 3A, 3B [41]. In the present study, 32 virus genomes showing sequence similarity to viruses belonging to the family *Picornaviridae* were also acquired. A phylogeny over the amino acid sequence of RdRp showed the relationship of the viruses identified here and their relatives (Fig. 6A). According to the sequence analysis and phylogeny, ten of these picornaviruses clustered with genus *Megrivirus*, three of them were closely related to viruses in genus *Oscivirus*, and one can be a member of the genus *Aalivirus*, these classifications were confirmed by sequence analysis based on the P1 protein. Four viruses were detected in white-backed woodpeckers, egrets and red-crowned cranes, respectively. Although they were clustered in an intermediate position among the clades of genera *Tremovirus*, *Hepatovirus*, and *Fipivirus*, they shared >34% sequence identities with their closest relatives (Supplementary Fig. 8A), belonging to the genus *Hepatovirus*. Five genomes from 3 different host bird species clustered together outside *Avihepatovirus* (Fig. 6A)*,* sharing > 34% sequence identities with their closest relatives (Supplementary Fig. 8B), belonging to the genus *Avihepatovirus.* Eight genomes from 7 different species of birds clustered together outside of the clades of genus *Avisivirus* and *Aalivirus*. These 8 viruses showed distinct genome organization, possessing an excess fragment (approximately 150 aa) at the downstream of 3CD region (Supplementary Fig. 8C), and shared amino acid sequence identity of < 34 % in P1 (Supplementary Fig. 8D) and < 36 % in 2C+3CD (Supplementary Fig. 8E), which suggest that these 8 viruses together form a possible new genus *Spleafvirus* (Fig. 6A). Although a novel picornavirus from red-flanked bush robin (*Tarsiger cyanurus*) fell outside of the clade which includes other 8 different defined genera based on phylogeny of RdRp protein (Fig. 6A), sequence analysis based on P1 sequence indicated that it shared > 44% amino acid sequence identity with those viruses belonging to *Kobuvirus*, suggesting this virus belongs to a member in the genus *Kobuvirus*.

Although viruses in the genus *Megivirus* are classified into 5 different species (i.e., *Megrivirus* A-E) according to their hosts, these viruses are evidently phylogenetically grouped into 2 separate clusters which also includes the 10 newly identified genomes in this study (Fig. 6A), where one cluster included megriviruses from birds belonging to 5 different bird orders while the other cluster of megriviruses only included those from birds belonging to the order *Galliformes*, suggesting that infection with this group of megriviruses was restricted in the birds belonging to this order.

Virus genomes showing sequence similarity to the family of *Dicistroviridae* (*n* = 118), *Iflaviridae* (*n* = 46)*, Totiviridae* (*n* = 28)*, Marnaviridae* (*n* = 11), and *Polycipiviridae* (*n* = 8) were assembled in libraries of these birds, although some of these viruses were too divergent to be classified into known genus in these virus families (Supplementary Fig. 9), these viruses most likely come from infected insects and other components of the diet of birds. Genomes of Weivirus-like virus (grouped into the unclassified *Riboviria*) (*n* = 7), Hepe-like virus (*n* = 6), and Noda-like virus (*n* = 5) were also detected in these birds (Supplementary Fig. 9), however, whether these viruses can replicate in bird hosts needs further study. The remaining 152 RNA viruses (except for Weivirus-like viruses) named unclassified *Picornavirales* or *Riboviria* were not phylogenetically related to known virus families (Fig. 6B and detailed phylogeny in Supplementary Fig. 10), some of which may be from the insect and plant diet of birds (or parasites within them) while other may conceivably be replicating in bird cells.

### Novel DNA viruses identified in the cloaca of birds

We also characterized 238 DNA viral genomes belonging to 8 different virus families or unclassified groups. Potential avian pathogens include viruses showing sequence similarity to *Parvoviridae* (*n* = 112), Unclassified CRESS-DNA virus (*n* = 66)*, Circoviridae* (*n* = 20), *Smacoviridae* (*n* = 12), *Genomoviridae* (*n* = 10), and *Adenoviridae* (*n* = 7) were identified (Fig. 1B).

**Fig. 7:**
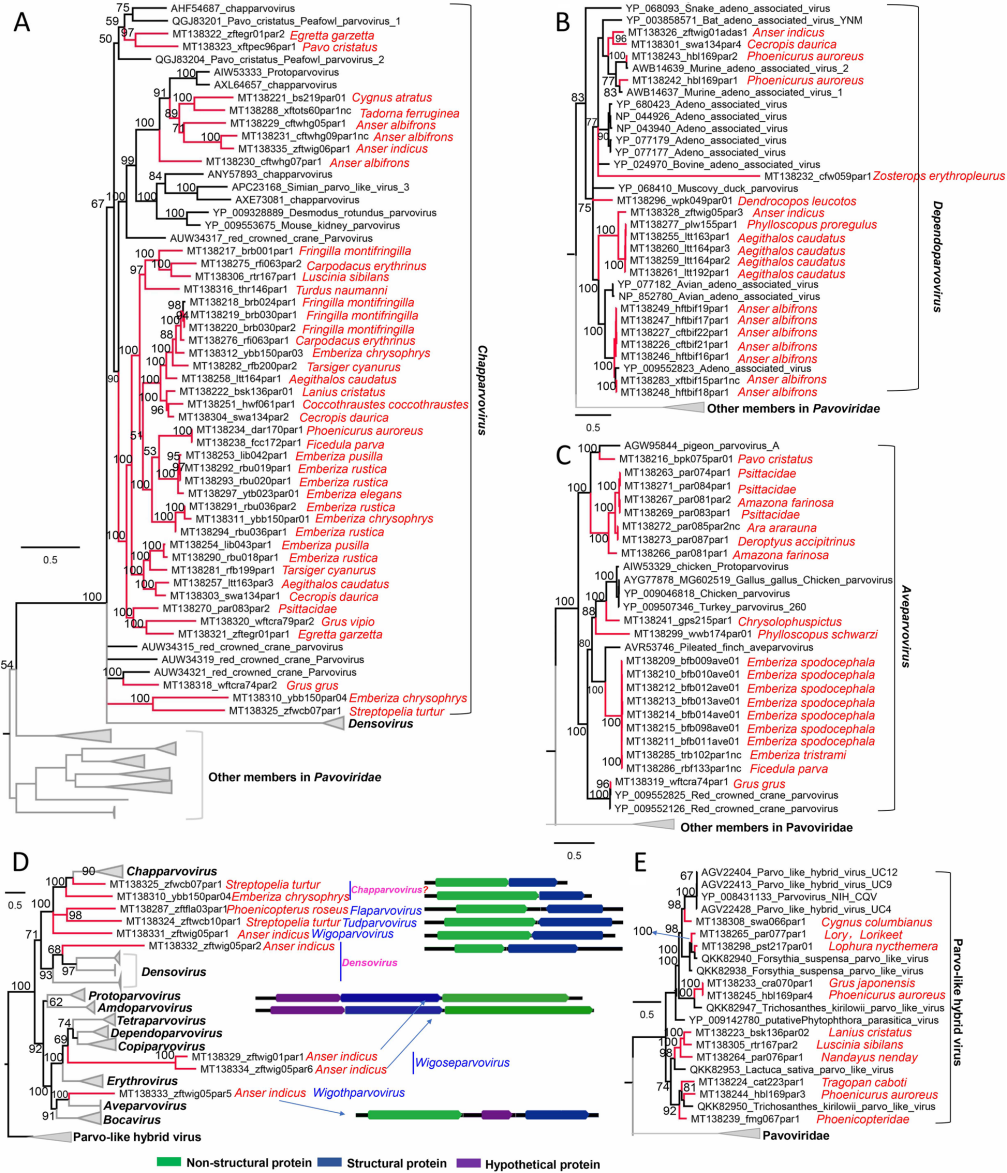
Phylogenies of Parvoviruses and parvo-like viruses identified in the cloaca of birds. **A** Bayesian inference tree established based on amino acid sequences of NS protein of *Chapparvovirus*. **B** Bayesian inference tree established based on amino acid sequences of NS protein of *Dependoparvovirus*. **C** Bayesian inference tree established based on amino acid sequences of NS protein of *Aveparvovirus*. **D** Bayesian inference tree established based on amino acid sequences of NS protein of these divergent parvoviruses which showed no close relationship to known parvovirus. Genome organization of each virus is also shown beside the corresponding strain. **E** Bayesian inference tree established based on amino acid sequences of NS protein of Parvo-like hybrid virus. Within trees, the viruses found in this study are marked with a red line. Species names of birds are indicated. Each scale bar indicates the amino acid substitutions per site

The classification criteria for virus belonging to the family *Parvoriridae* are that members of the same genus should share at least 35–40% amino acid sequence identity with a coverage of > 80% based on NS1 proteins and that parvoviruses can be considered members of the same species if their NS1 proteins share more than 85% amino acid sequence identity [42]. In the group of genomes showing similarity to parvovirus, 42 genomes from 28 different bird species were phylogenetically grouped into or close to the genus *Chapparvovirus* (Fig. 7A), with 8 strains closely clustered with previous species within the genus *Chapparvovirus* while most of the remaining strains were divergent to known species and therefore were potential new species within the genus as their NS1 proteins shared < 85% sequence identities to previously published parvoviruses. Nineteen genomes from different species of birds showed a close relationship to viruses belonging to the *Dependoparvovirus* genus, where only one strain clustered separately and might be a new species (Fig. 7B), sharing 51.2% sequence identity to its genetically closest relative within the genus *Dependoparvovirus*. Twenty genomes from 10 different species of birds were grouped into the genus of *Aveparvovirus* (Fig. 7C). Interestingly, 9 genomes from 4 different species of birds were too divergent from previously known genera within the family *Parvoviridae* to be grouped into any defined genus, being located between these known genera, except for two of them which may be grouped into the genus *Chapparvovirus* and one of them may be clustered into *Densovirus*; the other 6 genomes shared < 35% amino acid sequence identities with previously known parvoviruses based on NS1 proteins and formed five potential novel genera within the family *Parvoviridae* (temporarily named *Flaparvovirus*, *Tudparvovirus*, *Wigoparvovirus*, *Wigoseparvovirus*, and *Wigothparvovirus*) (Fig. 7D and Supplementary Table 2). Seven genomes of these 9 novel parvoviruses have typical genomic organization while 2 of them (MT138329 and MT138334) are both from wild bird species of *Anser indicus* and show atypical genomic size (> 7000 bp) and organization which contain an additional major ORF besides the two major ORFs encoding non-structural and capsid protein (Fig. 7D). Besides these genomes being grouped into the family *Parvoviridae*, 11 genomes from 10 different species of birds showed a close relationship to known parvo-like hybrid viruses [43] which belong to a new type of parvovirus-like genomes of uncertain origin possibly diatoms (Fig. 7E).

**Fig. 8:**
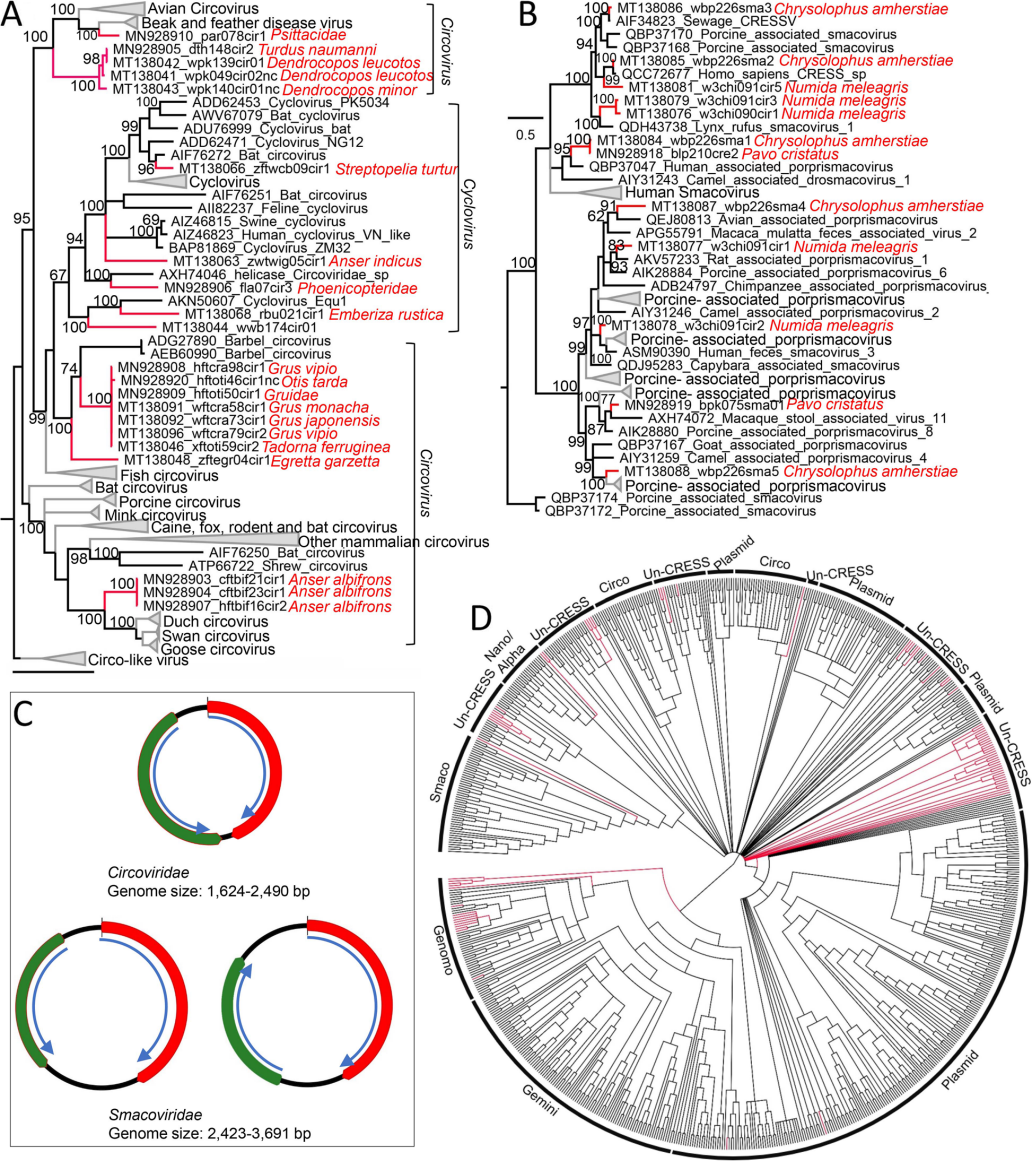
Phylogenies of circoviruses, smacoviruses, and other CRESS-DNA viruses identified in the cloaca of birds. **A** Bayesian inference tree established based on amino acid sequences of Rep protein of circoviruses. **B** Bayesian inference tree established based on amino acid sequences of Rep protein of smacovirus. **C** Genome organization of circoviruses and smacoviruses identified in this study. **D** Bayesian inference tree established based on amino acid sequences of Rep protein of other CRESS-DNA viruses. Within trees, the viruses found in this study are marked with a red line. Species names of birds are indicated. Each scale bar indicates the amino acid substitutions per site

Circular rep-encoding single-stranded DNA (CRESS DNA) viruses including those belonging to *Circoviridae* (*n* = 20), *Smacoviridae* (*n* = 12), *Genomoviridae* (*n* = 10), and unclassified CRESS DNA virus (*n* = 66) were identified from these samples. The 20 genomes belonging to *Circoviridae* were from 15 different species of birds and clustered into 7 different clades, with 5 genomes from wild birds closely related to beak and feather disease viruses (BFDV) infecting domestic poultries (Fig. 8 A). One circovirus genome (MT138066) from wild turtledoves showed a close relationship to a group of circoviruses from bats and shared the highest sequence identity of 86.2% to a bat circovirus (KJ641734) based on Rep protein sequence, suggesting this avian circovirus has a recent common ancestor with a bat circovirus. These circovirus genomes had a genome size of 1624–2490 nt and classic genomic organization encoding Rep protein and the Cap protein in the same orientation (Fig. 8B). Twelve smacovirus genomes were discovered in samples from 4 different species of birds and closely clustered with known smacoviruses previously identified in mammals (including human, pig, rat and lynx), birds and sewage samples (Fig. 8C). The host for viruses in the family *Smacoviridae* are currently unknown. Species and genus criteria demarcation of *Smacoviridae* are based on genome-wide and rep amino acid sequences with cut-off of 77.0% and 40.0%, respectively [44]. Based on these criteria, none of the 12 smacoviruses can be designated as new genera but 7 of them may be novel species within their corresponding genera (Supplementary Table 2). The remaining CRESS DNA viruses were phylogenetically analyzed together, where the genomoviruses were well grouped into their corresponding virus family. Although most of the unclassified CRESS DNA viruses identified in the present study are genetically related to previous unclassified CRESS DNA viruses, some of them were so divergent that they phylogenetically fell outside the all known CRESS DNA viruses (Fig. 8D and detailed phylogeny in Supplementary Fig. 11), suggesting some of these CRESS DNA viruses may comprise new virus families.

**Fig. 9:**
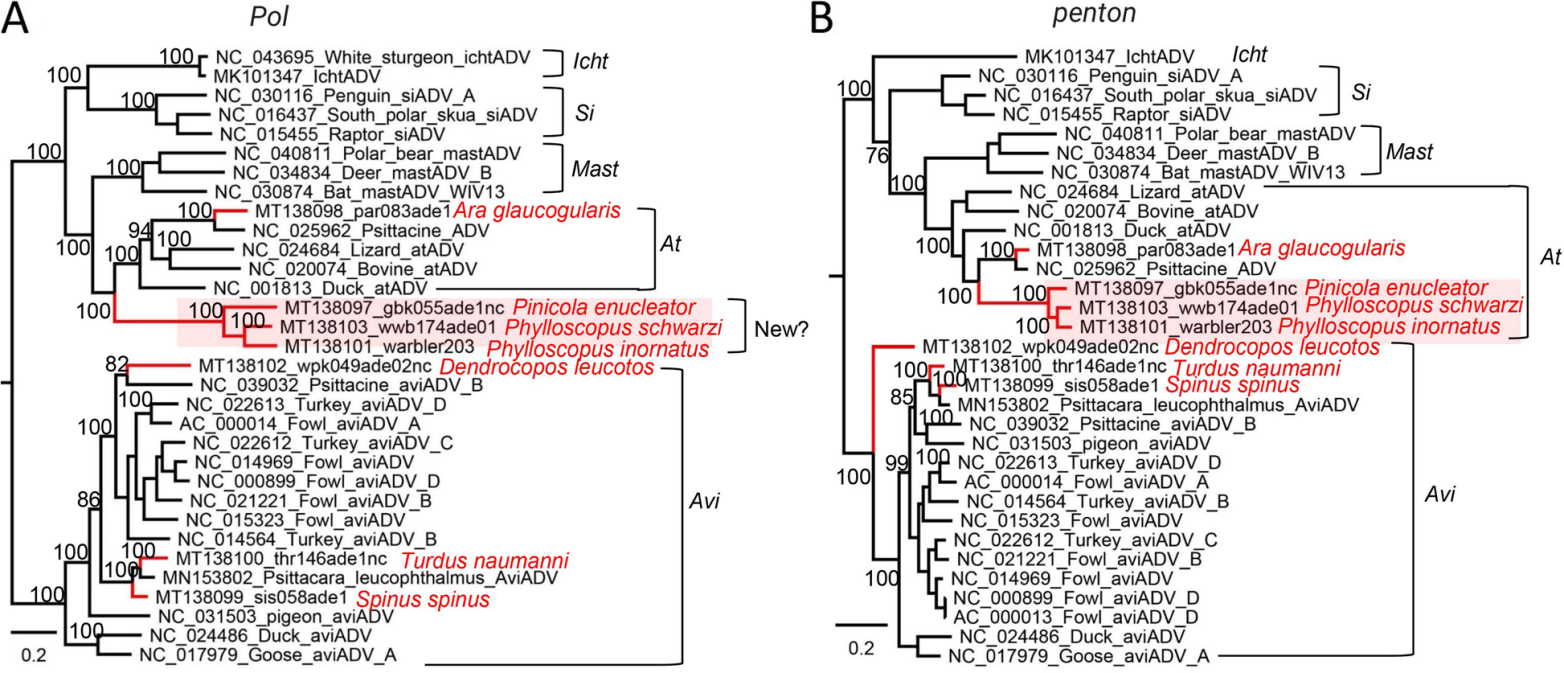
Phylogenies of adenoviruses identified in the cloaca of birds. **A** Bayesian inference tree established based on amino acid sequences of *pol* protein of adenovirus. **B** Bayesian inference tree established based on amino acid sequences of *penton* protein of adenoviruses. Within trees, the viruses found in this study are marked with a red line. Species names of birds are indicated. Each scale bar indicates the amino acid substitutions per site

Seven genomes showing sequence similarity to adenoviruses were identified in 7 different species of birds, 5 of which have sequence length > 30,000 nt and therefore are nearly complete genomes. Phylogenetic analysis based on the amino acid sequence of a non-structural protein (DNA polymerase) and a structural protein (penton) were performed (Fig. 9). To *Aviadenovirus* and *Atadenovirus*, the phylogenetic distance of DNA polymerase amino acid sequences greater than 5–15% is one of the several species demarcation criteria in these genera. Trees based on the DNA polymerase protein indicated that one strain (MT138098) from a parrot clustered with an adenovirus strain from another parrot (NC_025962) in the genus *Atadenovirus* sharing 76.63% sequence identity, suggesting it can be a novel species within the genus *Atadenovirus*. Another 3 strains from 3 different species of birds belonging to the order of *Passeriformes* were grouped outside of the genus *Atadenovirus* and shared 63.2–64.8% sequence identities with their best matches available in GenBank (Fig. 9A), suggesting the three may form a single novel species within genus *Atadenovirus*. However, the tree based on penton protein sequence showed the 3 strains together with the previously identified parrot adenovirus clustered into the same clade within the group of genus *Atadenovirus* (Fig. 9B), suggesting genomic recombination. The remaining 3 adenoviruses were from 3 different species of birds showed relationship to members of the genus *Aviadenovirus*, where the adenovirus from woodpecker (MT138102) clustered with a parrot adenovirus while the other two finch adenoviruses (MT138100 and MT138099) clustered with another parrot adenovirus.

Other viruses with more partial genomes showing sequence similarity to pathogens including influenza A virus, polyomavirus, and picornavirus were also detected in these birds.

## Discussion

Wild birds, including migratory ones, can be the long-distance transmitters of a variety of microorganisms. This creates the possibility of establishing novel foci of emerging or re-emerging infectious diseases along bird migration routes. Wild birds are the reservoirs of several viruses, including WNV, Eastern equine encephalitis virus, St. Louis encephalitis virus (SLE), Western equine encephalitis virus, and influenza viruses, among others [13, 45]. Birds are also the natural reservoirs of all of the pathogenic avian influenza A viruses. The spread of highly pathogenic avian influenza A virus (HPAI H5N1 Asian lineage) and WNV infection has resulted in greater attention to wild birds transmitting and spreading zoonotic pathogens. Some pathogens in migratory birds are more often isolated than in other animal species [46, 47]. The last two decades have seen the rise of viral metagenomics that has been used in numerous animal virome investigations including some avian viromes [48–75], providing information on the composition of animal viromes and helping to provide candidates to identify the etiology of infectious disease in birds and identify zoonotic and emerging viruses [76]. The present study investigated the virome in cloacal swab samples from 3182 birds belonging to > 87 different species, of which 620 samples were from breeding birds in zoos or farms and 2562 samples from wild birds in natural reserves or parks. Among these 2562 wild birds, 2083 were resident birds and the remaining 479 were migratory birds. Most of the resident birds analyzed belonged to 34 species within the order of *Passeriformes* which is the largest order in the Aves class, including more than 50% of all bird species. Birds of this order are especially active in places inhabited by humans and their pathogens and therefore pose a threat to both humans and other mammals. For example, the 6 coronavirus genomes identified here were all from *Passeriformes* birds, which together with other previous *Deltacoronavirus* sequences from birds were genetically related to *porcine deltacoronavirus*(PDCoV), an emerging entero-pathogen of swine with a worldwide distribution [77]. Some wild resident birds from the order of *Galliformes*, which are genetically close to domestic chickens, carry viruses that may be pathogenic to domestic chickens, for instance, some viral genomes which were genetically close to the chicken pathogenic megrivirus [78, 79], aveparvovirus [80], and gammaretrovirus [81] were reported here. Most of the wild migratory birds in this study were from the order of *Anseriformes* which are close relatives of domestic goose and duck. Thus, the viruses harbored by these species may be potentially infectious to these domestic poultries. For example, some of the calicivirus genomes discovered from wild geese here shared high sequence identities with previously reported caliciviruses from domestic geese and ducks.

Although most of the 469 RNA viruses identified in this study were closely related to known viruses, some of them grouped into unclassified *Riboviria*, which encode polyproteins containing the RdRp domain that is not genetically related to known RNA viruses. Whether these RdRp sequences are on viral genomes that can both replicate and be pathogenic to some birds will require future investigations as all the sampled animals analyzed here showed no obvious signs of disease. Apart from these RNA viruses with uncertain hosts, RNA viruses potentially infecting birds include those viruses belonging to the families *Astroviridae*, *Picornaviridae*, *Caliciviridae*, *Hepeviridae*, *Coronaviridae*, and *Retroviridae.* Among the 238 DNA viruses, nearly half belonged to the family *Parvoviridae* which contains many pathogenic members. Other potential pathogenic avian DNA viruses included members of the family *Circoviridae* and *Adenoviridae*. Bird infections with other viruses of these families can result in enteritis, bronchitis, reticuloendotheliosis, hepatitis, nephritis, and other disease signs [82–84].

The Aves class is considered to be the largest group of vertebrates, with > 10,000 species, and ubiquitously exists in ecosystems, which may be key factors in the spread and evolution of pathogenic avian viruses. A limited number of large-scale investigations of bird virome have been conducted [48, 70], and the genetic information of viruses harbored in wild birds is therefore still highly fragmentary. The family *Astroviridae* currently includes two genera, *Avastrovirus* and *Mamastrovirus*. The 39 astroviruses identified here all phylogenetically belong to the genus *Avastrovirus*. The genus *Avastrovirus* includes three species currently; *Avastrovirus* 1 (turkey astrovirus 1), *Avastrovirus* 2, (avian nephritis viruses 1 and 2, and chicken astrovirus), and *Avastrovirus* 3 (duck astrovirus, and turkey astrovirus 2). In the present study, although 15 astroviruses were grouped into the 3 defined species the remaining 24 strains have no close relatedness to known species and were phylogenetically located outside of the species *Avastrovirus* 3, which suggested the detection of new species of *Avastrovirus*. The family *Picornaviridae* presently comprises 47 genera with 110 species [85] and consists of genetically diverse viruses of vertebrates, where 12 of the 47 picornavirus genera infect birds. In the present study, although 32 different picornavirus genomes were acquired from these birds, only 14 genomes were well grouped into previously known genera including 10 strains belonging to *Megrivirus*, 3 strains belonging to *Oscivirus*, and 1 strain belonging to Aalivirus. The remaining 18 genomes were not closely related to any picornavirus genera, and may therefore comprise multiple new picornavirus genera significantly expanding the number of bird infecting picornavirus genera. Caliciviruses have been detected in an extensive broad range of vertebrate hosts; however, information relating to caliciviruses in wild birds is limited [86]. Here, 12 different calicivirus genomes were described and characterized, including 2 genomes from wild waterfowls (wild goose and shelduck) showing high sequence identities to previous caliciviruses from domestic poultries suggesting cross-species transmission. The other 10 calicivirus genomes may comprise several novel species or genera of calicivirus based on their divergence with currently known caliciviruses. Retroviruses discovered in birds included the common Avian sarcoma/leukosis viruses (ASLV) belonging to the *Alpharetrovirus* genus of the family *Retroviridae* and other types including reticuloendotheliosis virus (REV) and lymphoproliferative disease viru*s* of turkeys (LPDV) belonging to the genus *Gammaretrovirus* [87]. Here, apart from 2 genomes showing high sequence identity to REV were respectively detected in breeding golden pheasant and yellow-bellied tragopan (2 species in the order Galliformes), a new type of retrovirus was obtained from wild black swan (*Cygnus atratus*) which phylogenetically clustered with endogenous retroviruses from the genome of several species of birds and might be a new genus in the subfamily *Orthoretrovirinae*.

The *Parvoviridae* family includes two subfamilies, the *Parvovirinae* and *Densovirinae*, which are distinguished primarily by their respective ability to infect vertebrates (including humans) versus invertebrates. In the present study, we focus on vertebrates infecting parvovirus genomes including putative pathogenic ones showing a close relationship to *Chapparvovirus* and *Aveparvovirus* which are known agents of severe disease to vertebrates [80, 88]. Most of the new parvoviruses detected here belonged to the newly formed *Chaphamaparvovirus* genus. Detecting different *Chapparvovirus* in a variety of bird species suggested that this virus genus had a broad biogeographic distribution in bird species. Other interesting findings about parvovirus are those genomes without a close relationship to any known subfamily or genus within *Parvoviridae* which may include some pathogens to mammals or birds, especially the two parvovirus genomes which have atypical genome length and structure, where the structural protein ORF is located upstream of the non-structural protein and there is an additional ORF whose encoding protein showed no significant sequence similarity to any protein in the publica database but contain a putative domain of microbial transglutaminase. Whether these 2 parvoviruses can replicate in vertebrates or invertebrates needs further investigation. The family *Circoviridae* is a member of the big group of CRESS DNA viruses, which contains the smallest known viral pathogens of animals, including those infecting birds such as the beak and feather disease virus (BFDV )[89] and chicken anemia virus (CAV )[90]. In this study, 20 circovirus genomes that phylogenetically fell into the *Circoviridae* family were identified from 15 different species of wild and breeding birds, including those showing a close relationship to previously identified bird circoviruses and different mammalian ciroviruses, indicating the high genetic diversity of circovirus in bird populations. Apart from circovirus, numerous CRESS DNA virus genomes were also identified in these wild and breeding birds, some of which showed no close relationship to known CRESS DNA viruses thus were grouped into unclassified CRESS DNA viruses. In recent years, many genomes of unclassified CRESS DNA virus have been characterized in mammals, birds, insects, fungi, and environmental samples bringing to light a high level of genetic diversity among these viruses [91], whether these unclassified CRESS DNA viruses include members can replicate in bird hosts needs further investigation. The family *Adenoviridae* currently comprises five genera [92] which include *Mastadenovirus* that infect only mammalian species; *Aviadenovirus* that infect only birds; *Atadenovirus* that infect a broad host range, including reptiles, birds, opossums, and ruminants; *Siadenovirus* that includes adenoviruses of birds, reptiles, and amphibians; and *Ichtadenovirus* that includes adenoviruses of fish [93, 94]. In the current study, although some of the adenoviruses were well grouped into the genus of *Aviadenovirus* or *Atadenovirus*, some strains showed a signal of genomic recombination as trees based on different proteins revealed discordant topological structures.

## Conclusions

The cloaca viromes of 3182 birds belonging to > 87 different species in 10 different orders were investigated. A total of 707 viral genomes were assembled from these viromes and classified into 19 defined families and 4 unclassified virus groups. New virus species, genera, or families were identified in these wild or breeding birds. Putative cross-species transmissions and genomic recombination were observed with some viruses identified here. This study has largely expanded our understanding of birds’ viral diversity. Further molecular epidemiological studies are needed to explore the diversity, biology, and detailed pathogenesis of these viruses in domestic and wild bird species.

## Supplementary Information

**Additional file 1: Supplementary Fig. 1** Map with sampling sites. The sampling sites are marked with red dots and labelled with province names.

**Additional file 2: Supplementary Fig. 2** Virome analysis in the environmental samples collected in laboratory. Heatmap representing the read number of each viral family in each library (see color legend). Outline of possible host species and sample ID are shown with corresponding colors.

**Additional file 3: Supplementary Fig. 3** Virus distribution in the 215 birds’ cloacal swab pools based on birds’ order. The horizontal ordinate represents different virus genomes which are further divided into 24 different virus families or groups, while the longitudinal axis represents the 215 libraries that are arranged based on birds’ birds’ order (10 different orders of birds). Heatmap representing the read number in mapping of the 707 genomes against NGS data of the 215 libraries (see color legend).

**Additional file 4: Supplementary Fig. 4** Virus distribution in the 215 birds’ cloacal swab pools based on birds’ behavioral feature. The horizontal ordinate represents different virus genomes which are further divided into 24 different virus families or groups, while the longitudinal axis represents the 215 libraries that are arranged based on birds’ behavioral feature (migratory and resident). Heatmap representing the read number in mapping of the 707 genomes against NGS data of the 215 libraries (see color legend).

**Additional file 5: Supplementary Fig. 5** Virus distribution in the 215 birds’ cloacal swab pools based on birds’ sampling site. The horizontal ordinate represents different virus genomes which are further divided into 24 different virus families or groups, while the longitudinal axis represents the 215 libraries that are arranged based on birds’ sampling site. Heatmap representing the read number in mapping of the 707 genomes against NGS data of the 215 libraries (see color legend).

**Additional file 6: Supplementary Fig. 6** Phylogenies of astroviruses identified in cloaca of birds. (A) Bayesian inference tree established based on amino acid sequences of capsid protein of astroviruses. Within trees the viruses found in this study are marked with red line. Species names of birds are indicated. Each scale bar indicates the amino acid substitutions per site. (B) Phylogenies of RdRp (left) and capsid (right) protein sequences of astroviruses with putative genomic recombination.

**Additional file 7: Supplementary Fig. 7** Phylogenetic analysis based on complete CDS of the 6 coronaviruses and those related representative deltacoronavirus genomes. Within trees the viruses found in this study are marked with red line. Scale bar indicates the nucleotide substitutions per site.

**Additional file 8: Supplementary Fig. 8** The sequence identity between the newly identified pircornaviruses and their closest relatives (A, B, D, and E) and the genome organization feature for the viruses qualified to be a putative new genus (C).

**Additional file 9: Supplementary Fig. 9** The phylogenies of other RNA viruses possibly being from the diet of birds. Eight Bayesian inference trees were established using MrBayes v3.2 based on RdRp proteins, within each tree, the viruses found in this study are marked with red line. The names of the virus family or type are shown on the top of each tree. Each scale bar indicates 0.5 amino acid substitutions per site.

**Additional file 10: Supplementary Fig. 10** Bayesian inference tree based on amino acid sequences of RdRp of viruses belonging to unclassified *Picornavirales* and *Riboviria* identified here. Within trees the viruses found in this study are marked with red line. (For clear figure with high resolution please see the separately Fig. S9 uploaded in the submission system)

**Additional file 11: Supplementary Fig. 11** Bayesian inference tree based on amino acid sequences of Rep of the unclassified CRESS DNA viruses identified here. Within trees the viruses found in this study are marked with red line. (For clear figure with high resolution please see the separately Fig. S10 uploaded in the submission system)

**Additional file 12: Supplementary Table 1**. Information of bird species and library included in the present study.

**Additional file 13: Supplementary Table 2**. Information of viruses with complete or nearly complete genome identified in cloaca of birds.

**Additional file 14: Supplementary Table 3**. Number of sequence reads of the mapping analysis using the 707 virus genomes respectively against the 215 NGS data.

**Additional file 15: Supplementary Table 4**. ICTV criteria for new genus or species of viruses identified in this study.

**Additional file 16: Supplementary Table 5**. Primers used in the PCR confirmation of the 10 unclassified CRESS DNA viruses and 10 unclassified Riboviria genomes in the original sample pools of birds’ cloacal swabs.

## Data Availability

The 215 sets of sequence reads data generated on the Illumina sequencing platform in this study were deposited into the Sequence Read Archive of GenBank database and the accession nos. are shown in Supplementary Table 1. All complete or partial viral genomes acquired in this study were deposited in GenBank and the accession nos. are provided in Supplementary Table 2.

## Abbreviations

SARS-CoV2: Severe acute respiratory syndrome coronavirus 2
AIV: Avian influenza virus
NDV: Newcastle disease virus
FPV: Fowlpox virus
DPV: Duck plague virus
FMDV: Foot and mouth disease virus
JEV: Japanese encephalitis virus
WNV: West Nile virus
DPBS: Dulbecco’s phosphate-buffered saline
NVNR: Non-virus non-redundant
CDS: Complete coding sequence
mcmc: Markov chain Monte Carlo
ORFs: Open reading frames
RdRp: RNA-dependent RNA polymerase
Rep: Replication proteins
BFDV: Beak and feather disease virus
SLE: St. Louis encephalitis virus
ASLV: Avian sarcoma/leukosis virus
REV: Reticuloendotheliosis virus
LPDV: Lymphoproliferative disease virus
