# Supplementary figures and images for: Virome in the cloaca of wild and breeding birds revealed a diversity of significant viruses
Source: Microbiome. 2022 Apr 12;10:60. doi: 10.1186/s40168-022-01246-7 (PMC9001828; doi:10.1186/s40168-022-01246-7)

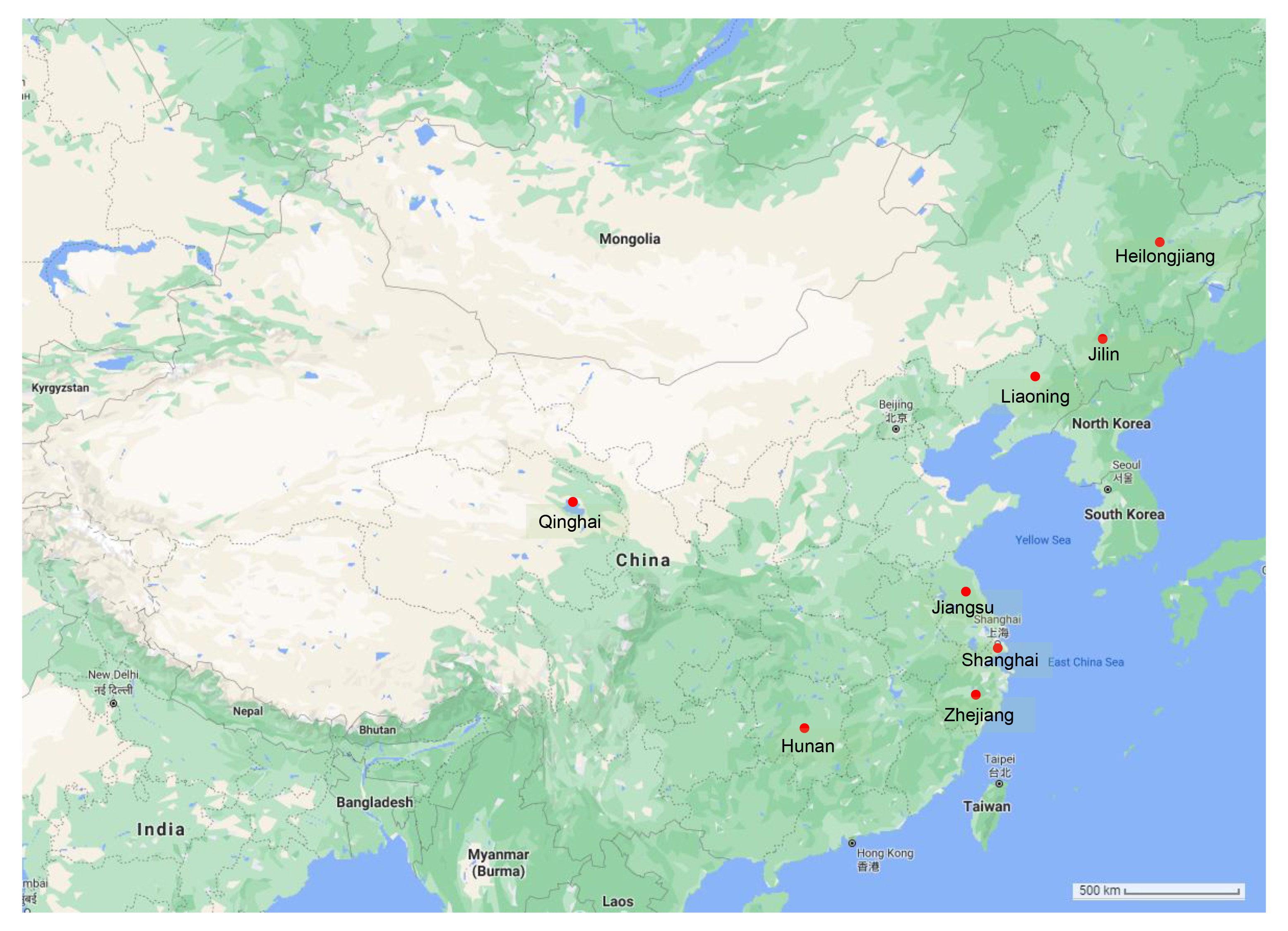

Supplement: Supplementary file 2 — Additional file 1: Supplementary Fig. 1 Map with sampling sites. The sampling sites are marked with red dots and labelled with province names. [file 40168_2022_1246_MOESM1_ESM.jpg]

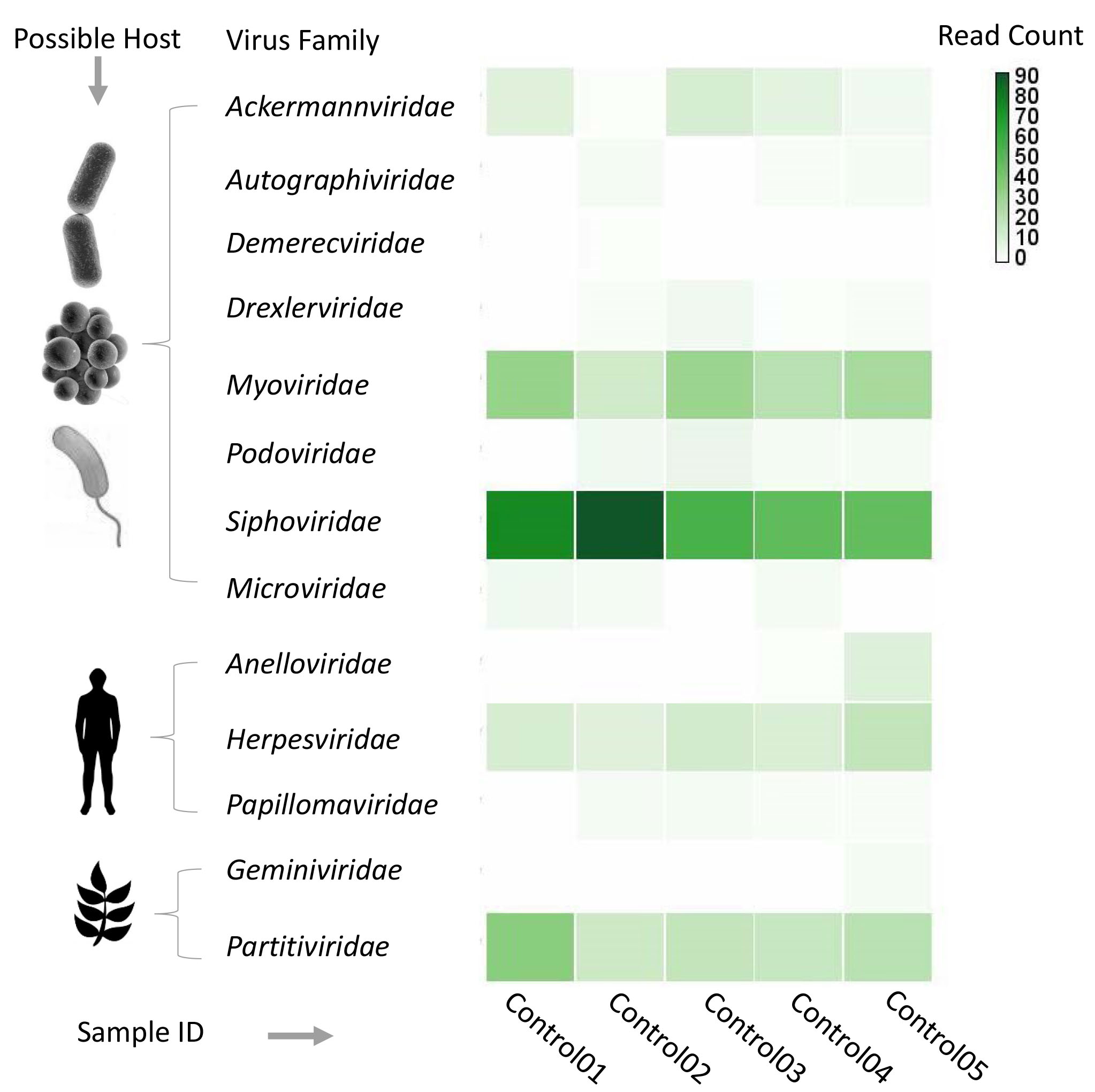

Supplement: Supplementary file 3 — Additional file 2: Supplementary Fig. 2 Virome analysis in the environmental samples collected in laboratory. Heatmap representing the read number of each viral family in each library (see color legend). Outline of possible host species and sample ID are shown with corresponding colors. [file 40168_2022_1246_MOESM2_ESM.jpg]

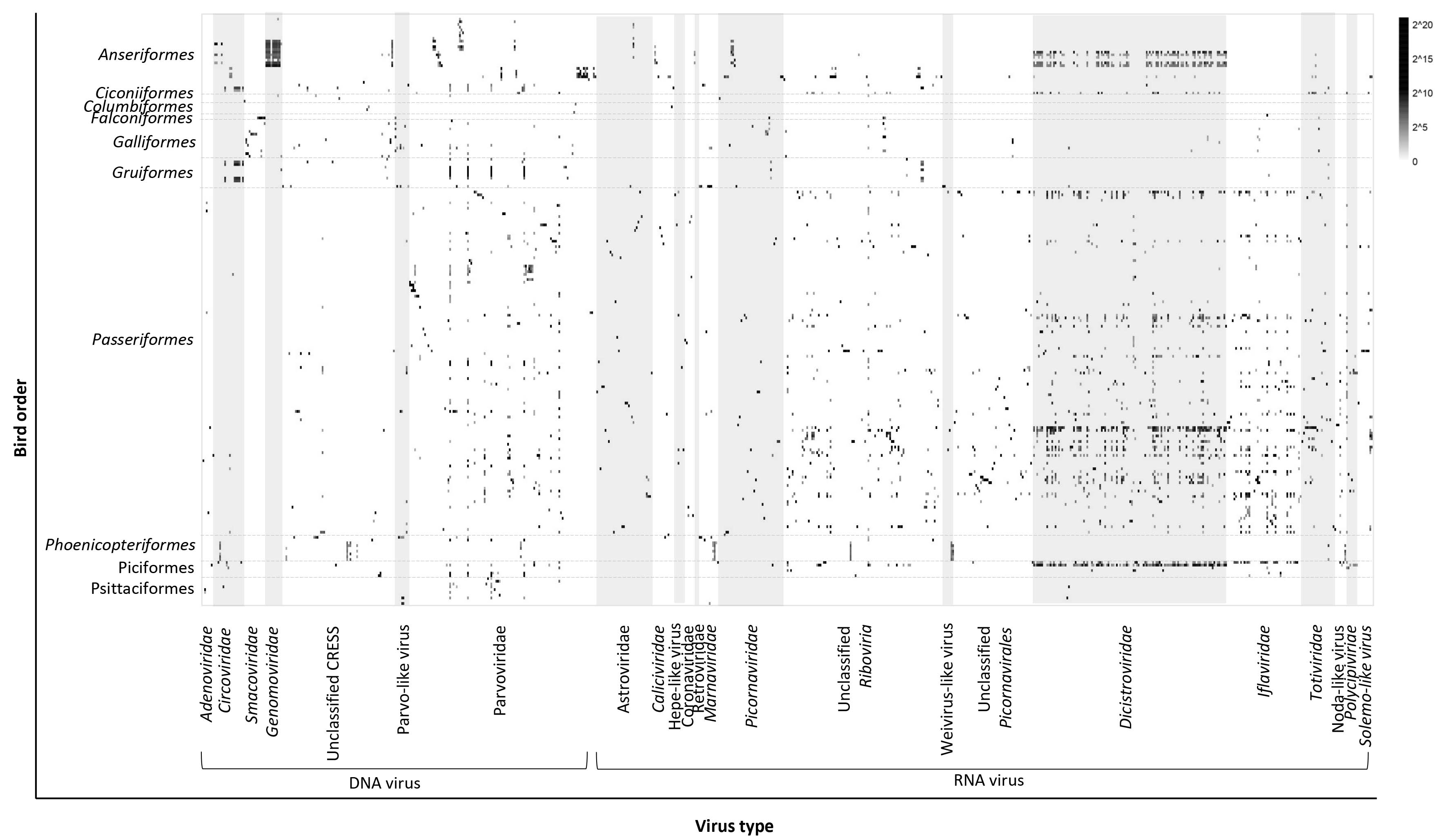

Supplement: Supplementary file 4 — Additional file 3: Supplementary Fig. 3 Virus distribution in the 215 birds’ cloacal swab pools based on birds’ order. The horizontal ordinate represents different virus genomes which are further divided into 24 different virus families or groups, while the longitudinal axis represents the 215 libraries that are arranged based on birds’ birds’ order (10 different orders of birds). Heatmap representing the read number in mapping of the 707 genomes against NGS data of the 215 libraries (see color legend). [file 40168_2022_1246_MOESM3_ESM.jpg]

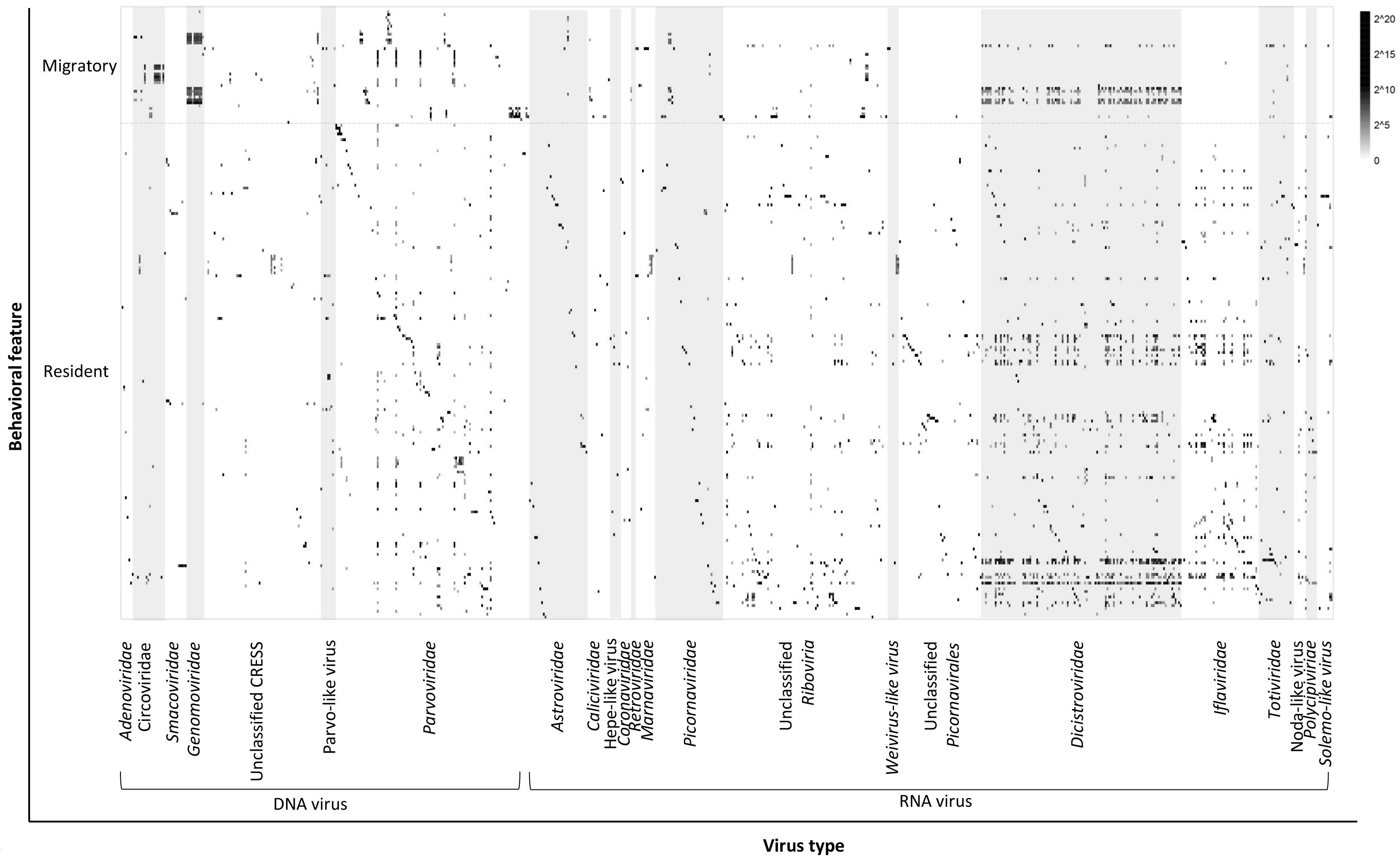

Supplement: Supplementary file 5 — Additional file 4: Supplementary Fig. 4 Virus distribution in the 215 birds’ cloacal swab pools based on birds’ behavioral feature. The horizontal ordinate represents different virus genomes which are further divided into 24 different virus families or groups, while the longitudinal axis represents the 215 libraries that are arranged based on birds’ behavioral feature (migratory and resident). Heatmap representing the read number in mapping of the 707 genomes against NGS data of the 215 libraries (see color legend). [file 40168_2022_1246_MOESM4_ESM.jpg]

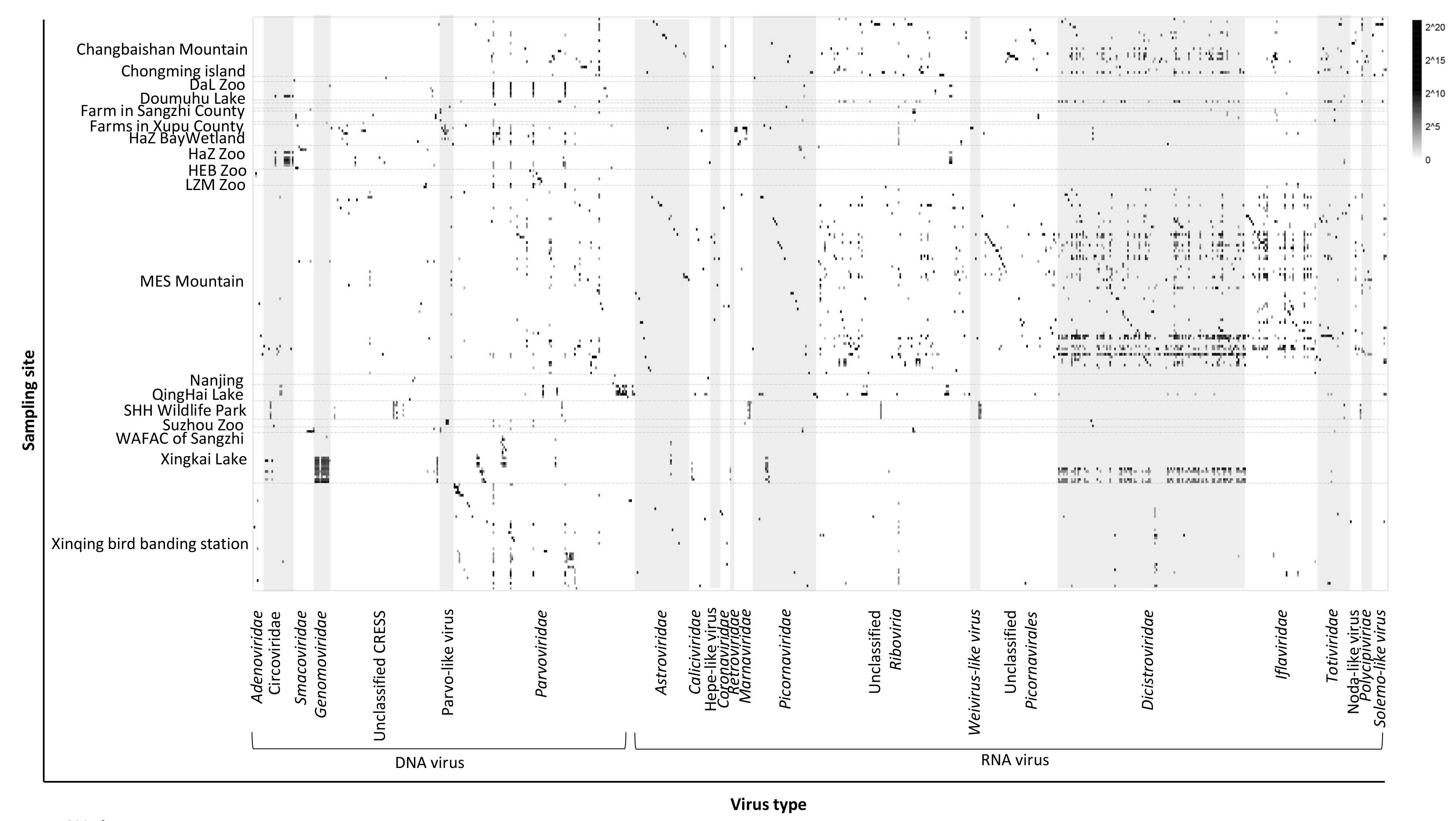

Supplement: Supplementary file 6 — Additional file 5: Supplementary Fig. 5 Virus distribution in the 215 birds’ cloacal swab pools based on birds’ sampling site. The horizontal ordinate represents different virus genomes which are further divided into 24 different virus families or groups, while the longitudinal axis represents the 215 libraries that are arranged based on birds’ sampling site. Heatmap representing the read number in mapping of the 707 genomes against NGS data of the 215 libraries (see color legend). [file 40168_2022_1246_MOESM5_ESM.jpg]

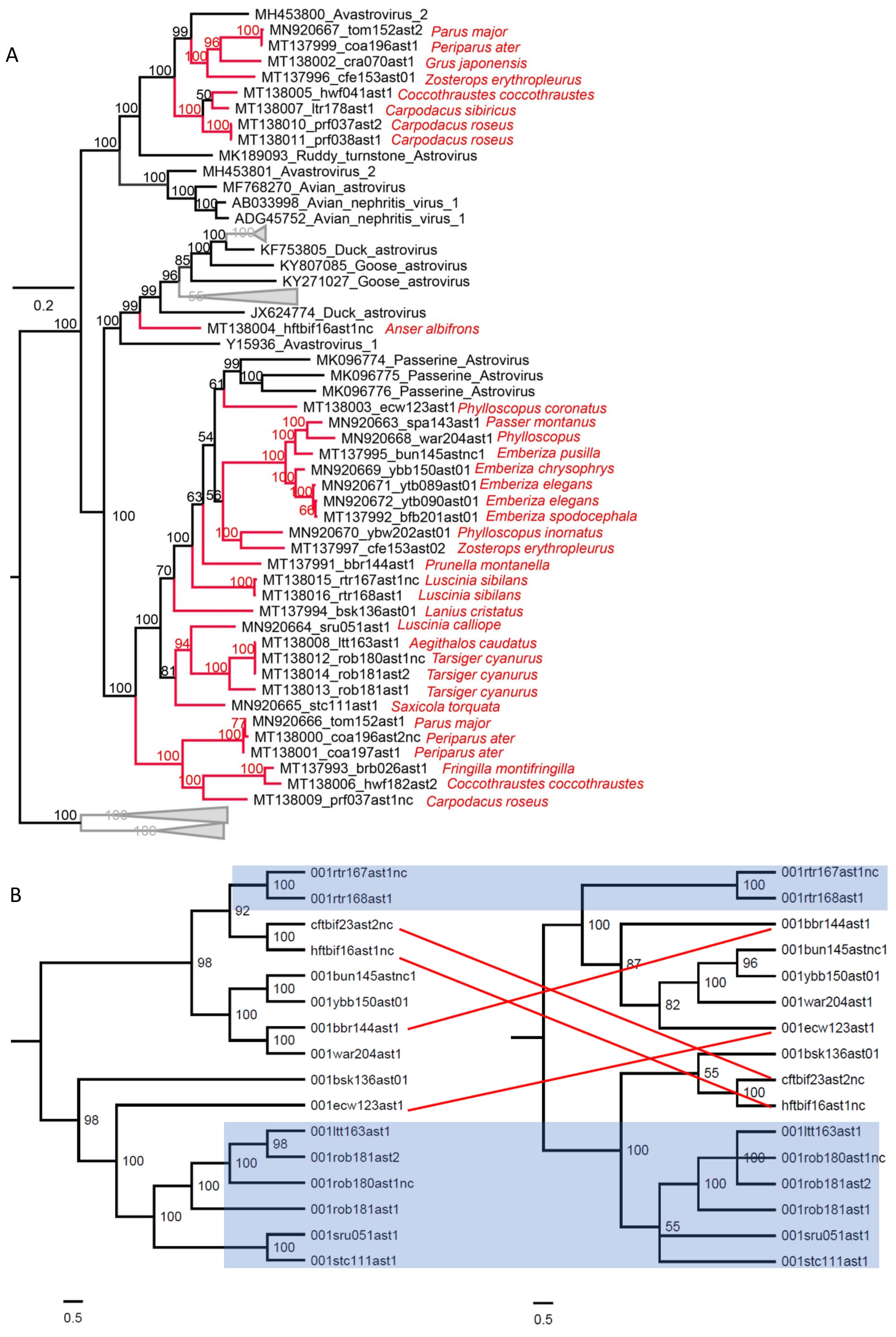

Supplement: Supplementary file 7 — Additional file 6: Supplementary Fig. 6 Phylogenies of astroviruses identified in cloaca of birds. (A) Bayesian inference tree established based on amino acid sequences of capsid protein of astroviruses. Within trees the viruses found in this study are marked with red line. Species names of birds are indicated. Each scale bar indicates the amino acid substitutions per site. (B) Phylogenies of RdRp (left) and capsid (right) protein sequences of astroviruses with putative genomic recombination. [file 40168_2022_1246_MOESM6_ESM.jpg]

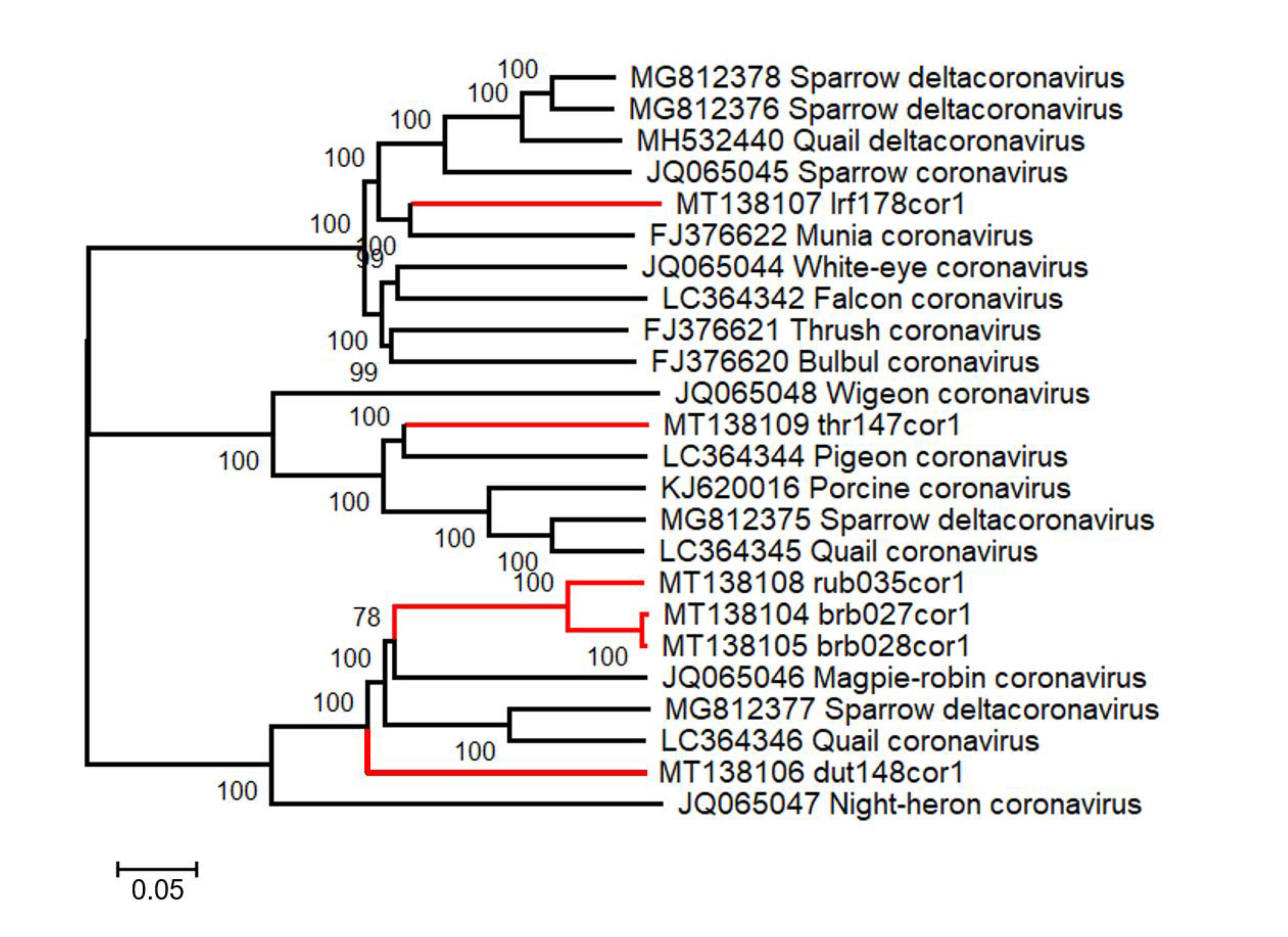

Supplement: Supplementary file 8 — Additional file 7: Supplementary Fig. 7 Phylogenetic analysis based on complete CDS of the 6 coronaviruses and those related representative deltacoronavirus genomes. Within trees the viruses found in this study are marked with red line. Scale bar indicates the nucleotide substitutions per site. [file 40168_2022_1246_MOESM7_ESM.jpg]

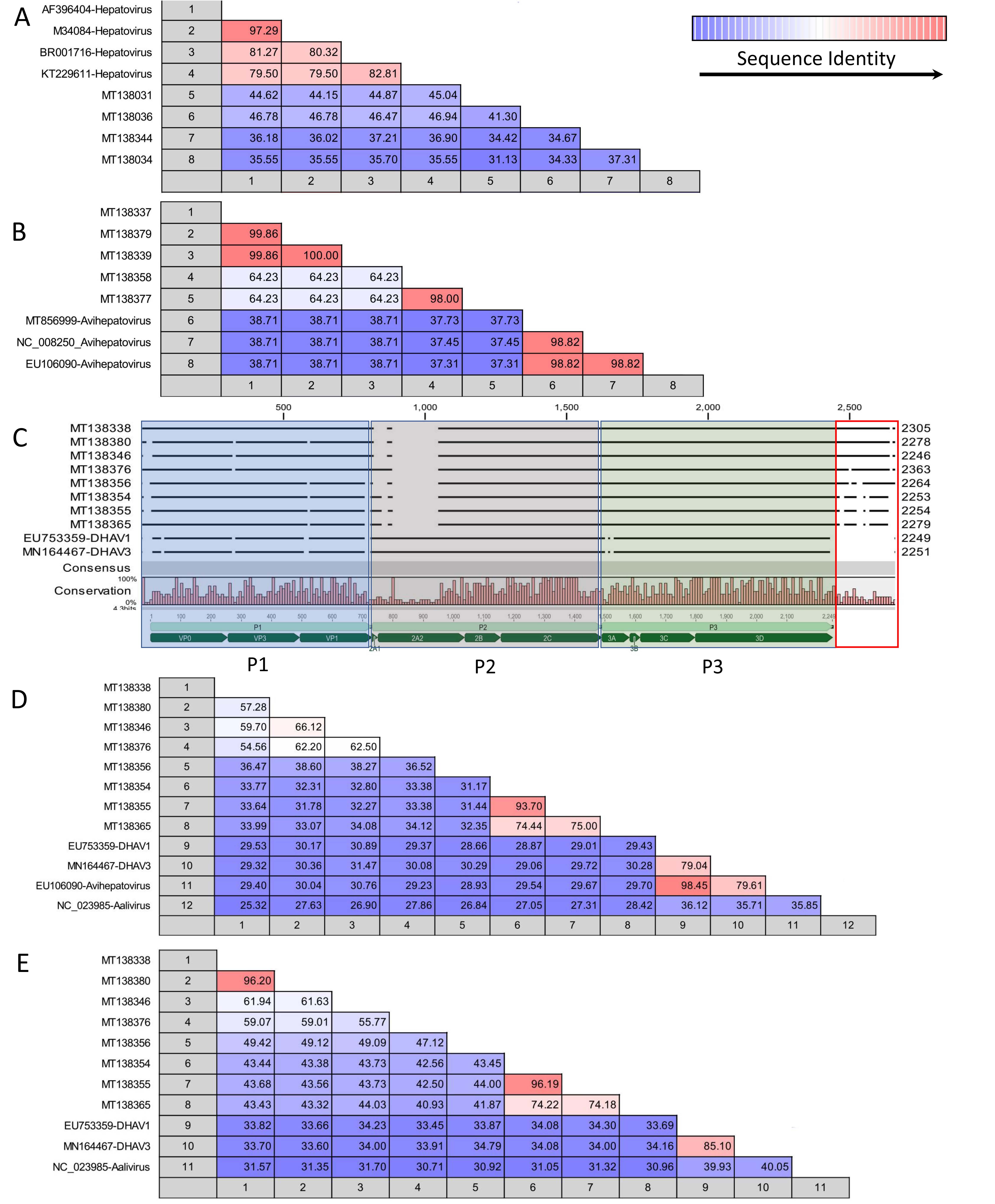

Supplement: Supplementary file 9 — Additional file 8: Supplementary Fig. 8 The sequence identity between the newly identified pircornaviruses and their closest relatives (A, B, D, and E) and the genome organization feature for the viruses qualified to be a putative new genus (C). [file 40168_2022_1246_MOESM8_ESM.jpg]

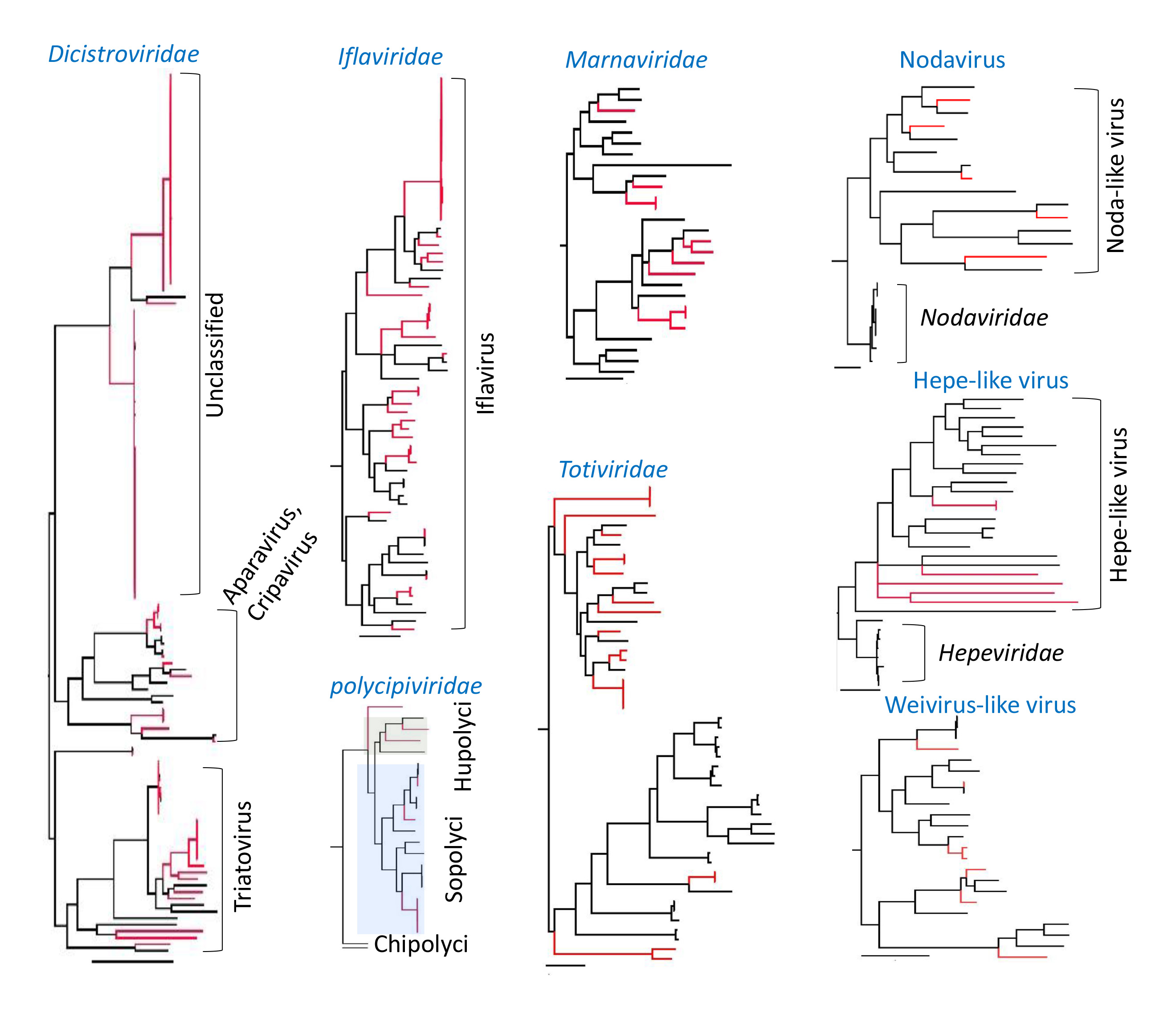

Supplement: Supplementary file 10 — Additional file 9: Supplementary Fig. 9 The phylogenies of other RNA viruses possibly being from the diet of birds. Eight Bayesian inference trees were established using MrBayes v3.2 based on RdRp proteins, within each tree, the viruses found in this study are marked with red line. The names of the virus family or type are shown on the top of each tree. Each scale bar indicates 0.5 amino acid substitutions per site. [file 40168_2022_1246_MOESM9_ESM.jpg]

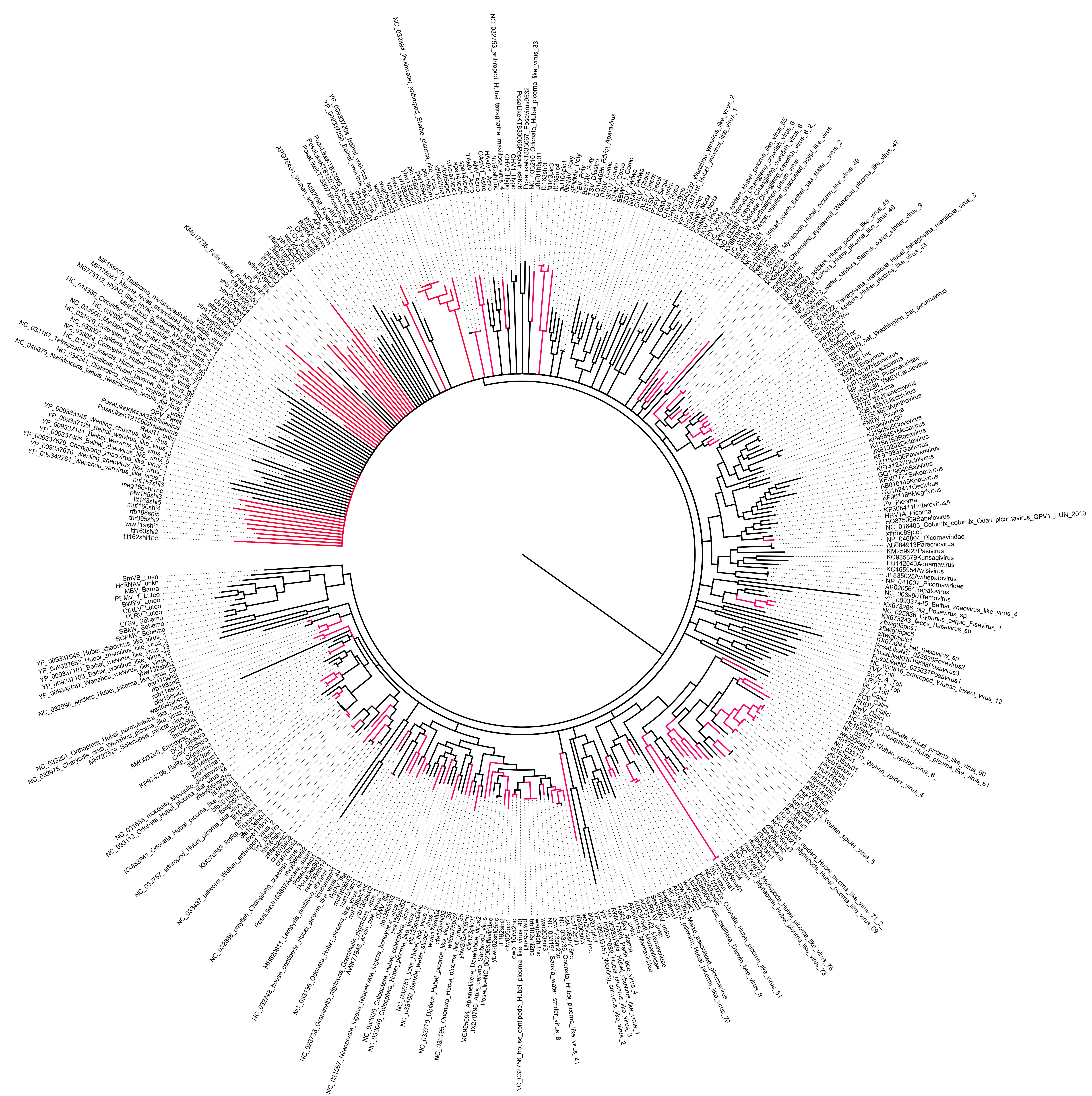

Supplement: Supplementary file 11 — Additional file 10: Supplementary Fig. 10 Bayesian inference tree based on amino acid sequences of RdRp of viruses belonging to unclassified Picornavirales and Riboviria identified here. Within trees the viruses found in this study are marked with red line. (For clear figure with high resolution please see the separately Fig. S9 uploaded in the submission system) [file 40168_2022_1246_MOESM10_ESM.jpg]
