# Supplementary material for: Virome in the cloaca of wild and breeding birds revealed a diversity of significant viruses
Source: Microbiome. 2022 Apr 12;10:60. doi: 10.1186/s40168-022-01246-7 (PMC9001828; doi:10.1186/s40168-022-01246-7)
Supplement: Supplementary file 13 — Additional file 12: Supplementary Table 1. Information of bird species and library included in the present study. [file 40168_2022_1246_MOESM12_ESM.docx]

**Supplementary Table 1. Information of bird species and library included in the present study.**

| **SRA no.** | **Location of Sampling (Sampling site, Province)** | **Habitat of bird** | **Number of sample** | **Bird English name** | **Order name** | **Family name** | **Genus name** | **Species name** | **Library total reads** | **Uniue reads** |
| --- | --- | --- | --- | --- | --- | --- | --- | --- | --- | --- |
| SRX7543495 | QingHai Lake, Qinghai | Wild | 10 | Bar-headed Goose | *Anseriformes* | *Anatidae* | *Anser* | *Anser indicus* | 4596860 | 3136634 |
| SRX7543508 | QingHai Lake, Qinghai | Wild | 10 | Bar-headed Goose | *Anseriformes* | *Anatidae* | *Anser* | *Anser indicus* | 467260 | 301035 |
| SRX7543513 | QingHai Lake, Qinghai | Wild | 10 | Bar-headed Goose | *Anseriformes* | *Anatidae* | *Anser* | *Anser indicus* | 955624 | 665324 |
| SRX7543521 | QingHai Lake, Qinghai | Wild | 10 | Bar-headed Goose | *Anseriformes* | *Anatidae* | *Anser* | *Anser indicus* | 303448 | 235462 |
| SRX7543524 | QingHai Lake, Qinghai | Wild | 10 | Bar-headed Goose | *Anseriformes* | *Anatidae* | *Anser* | *Anser indicus* | 34772 | 23254 |
| SRX7543526 | QingHai Lake, Qinghai | Wild | 10 | Bar-headed Goose | *Anseriformes* | *Anatidae* | *Anser* | *Anser indicus* | 242400 | 150213 |
| SRX7543568 | Xingkai Lake, Heilongjiang | Wild | 20 | White-fronted Goose | *Anseriformes* | *Anatidae* | *Anser* | *Anser albifrons* | 622756 | 419854 |
| SRX7543620 | Xingkai Lake, Heilongjiang | Wild | 20 | White-fronted Goose | *Anseriformes* | *Anatidae* | *Anser* | *Anser albifrons* | 760200 | 565421 |
| SRX7543645 | Xingkai Lake, Heilongjiang | Wild | 20 | White-fronted Goose | *Anseriformes* | *Anatidae* | *Anser* | *Anser albifrons* | 1052988 | 621547 |
| SRX7543668 | Xingkai Lake, Heilongjiang | Wild | 20 | White-fronted Goose | *Anseriformes* | *Anatidae* | *Anser* | *Anser albifrons* | 477708 | 356879 |
| SRX7543670 | Xingkai Lake, Heilongjiang | Wild | 20 | White-fronted Goose | *Anseriformes* | *Anatidae* | *Anser* | *Anser albifrons* | 679888 | 412654 |
| SRX7543681 | Xingkai Lake, Heilongjiang | Wild | 20 | White-fronted Goose | *Anseriformes* | *Anatidae* | *Anser* | *Anser albifrons* | 476508 | 356241 |
| SRX7543511 | HaZ Zoo, Zhejiang | Breeding | 20 | Tundra Swan | *Anseriformes* | *Anatidae* | *Cygnus* | *Cygnus columbianus* | 2746084 | 1898754 |
| SRX7543515 | HEB Zoo, Heilongjiang | Breeding | 13 | Ruddy Shelduck | *Anseriformes* | *Anatidae* | *Tadorna* | *Tadorna ferruginea* | 379202 | 290254 |
| SRX7543510 | HEB Zoo, Heilongjiang | Breeding | 13 | Ruddy Shelduck | *Anseriformes* | *Anatidae* | *Tadorna* | *Tadorna ferruginea* | 667406 | 485645 |
| SRX7551871 | Xingkai Lake, Heilongjiang | Wild | 19 | Greater White-fronted Goose | *Anseriformes* | *Anatidae* | *Anser* | *Anser albifrons* | 900100 | 532154 |
| SRX7551869 | Xingkai Lake, Heilongjiang | Wild | 19 | Greater White-fronted Goose | *Anseriformes* | *Anatidae* | *Anser* | *Anser albifrons* | 1185888 | 852136 |
| SRX7551861 | Xingkai Lake, Heilongjiang | Wild | 20 | Greater White-fronted Goose | *Anseriformes* | *Anatidae* | *Anser* | *Anser albifrons* | 169698 | 125263 |
| SRX7551691 | Xingkai Lake, Heilongjiang | Wild | 20 | Greater White-fronted Goose | *Anseriformes* | *Anatidae* | *Anser* | *Anser albifrons* | 127468 | 91235 |
| SRX7551688 | Xingkai Lake, Heilongjiang | Wild | 20 | Greater White-fronted Goose | *Anseriformes* | *Anatidae* | *Anser* | *Anser albifrons* | 112556 | 84569 |
| SRX7551687 | Xingkai Lake, Heilongjiang | Wild | 19 | Greater White-fronted Goose | *Anseriformes* | *Anatidae* | *Anser* | *Anser albifrons* | 142152 | 76324 |
| SRX7551682 | Xingkai Lake, Heilongjiang | Wild | 20 | Greater White-fronted Goose | *Anseriformes* | *Anatidae* | *Anser* | *Anser albifrons* | 224402 | 138547 |
| SRX7551645 | Xingkai Lake, Heilongjiang | Wild | 21 | Greater White-fronted Goose | *Anseriformes* | *Anatidae* | *Anser* | *Anser albifrons* | 78288 | 55423 |
| SRX7545081 | Xingkai Lake, Heilongjiang | Wild | 21 | Greater White-fronted Goose | *Anseriformes* | *Anatidae* | *Anser* | *Anser albifrons* | 664178 | 452364 |
| SRX7544648 | Xingkai Lake, Heilongjiang | Wild | 21 | Greater White-fronted Goose | *Anseriformes* | *Anatidae* | *Anser* | *Anser albifrons* | 1543262 | 1156875 |
| SRX7544567 | Xingkai Lake, Heilongjiang | Wild | 21 | Greater White-fronted Goose | *Anseriformes* | *Anatidae* | *Anser* | *Anser albifrons* | 124000 | 89564 |
| SRX7544561 | Xingkai Lake, Heilongjiang | Wild | 21 | Greater White-fronted Goose | *Anseriformes* | *Anatidae* | *Anser* | *Anser albifrons* | 242708 | 198756 |
| SRX7544557 | Xingkai Lake, Heilongjiang | Wild | 21 | Greater White-fronted Goose | *Anseriformes* | *Anatidae* | *Anser* | *Anser albifrons* | 1053680 | 796542 |
| SRX7543532 | HaZ Bay Wetland Park, Zhejiang | Wild | 17 | White Stork | *Ciconiiformes* | *Ciconiidae* | *Ciconia* | *Ciconia boyciana* | 254662 | 198756 |
| SRX7543718 | HaZ Zoo, Zhejiang | Breeding | 5 | Lesser Flamingo | *Phoenicopteriformes* | *Phoenicopteridae* | *Phoeniconaias* | *Phoeniconaias minor* | 5765968 | 3901354 |
| SRX7544108 | SHH Wildlife Park, Shanghai | Breeding | 10 | Flamingo | *Phoenicopteriformes* | *Phoenicopteridae* | *Phoenicopterus* | *Phoenicopterus roseus* | 4138072 | 2651243 |
| SRX7544114 | SHH Wildlife Park, Shanghai | Breeding | 10 | Flamingo | *Phoenicopteriformes* | *Phoenicopteridae* | *Phoenicopterus* | *Phoenicopterus roseus* | 4680636 | 4013265 |
| SRX7544115 | SHH Wildlife Park, Shanghai | Breeding | 10 | Flamingo | *Phoenicopteriformes* | *Phoenicopteridae* | *Phoenicopterus* | *Phoenicopterus roseus* | 2960364 | 1654325 |
| SRX7544116 | SHH Wildlife Park, Shanghai | Breeding | 10 | Flamingo | *Phoenicopteriformes* | *Phoenicopteridae* | *Phoenicopterus* | *Phoenicopterus roseus* | 2188332 | 1756798 |
| SRX7544124 | Chongming island, Shanghai | Wild | 10 | Egret | *Ciconiiformes* | *Ardeidae* | *Egretta* | *Egretta garzetta* | 1702592 | 1321548 |
| SRX7544125 | Chongming island, Shanghai | Wild | 10 | Egret | *Ciconiiformes* | *Ardeidae* | *Egretta* | *Egretta garzetta* | 3076148 | 2325984 |
| SRX7543638 | Nanjing, Jiangsu | Wild | 10 | Turtle Dove | *Columbiformes* | *Columbidae* | *Streptopelia* | *Streptopelia turtur* | 281004 | 201654 |
| SRX7543644 | Nanjing, Jiangsu | Wild | 10 | Turtle Dove | *Columbiformes* | *Columbidae* | *Streptopelia* | *Streptopelia turtur* | 545112 | 400211 |
| SRX7543652 | Nanjing, Jiangsu | Wild | 10 | Turtle Dove | *Columbiformes* | *Columbidae* | *Streptopelia* | *Streptopelia turtur* | 482224 | 321365 |
| SRX7543677 | Nanjing, Jiangsu | Wild | 10 | Turtle Dove | *Columbiformes* | *Columbidae* | *Streptopelia* | *Streptopelia turtur* | 188622 | 124491 |
| SRX7544119 | MES Mountain, Heilongjiang | Wild | 10 | Japanese Sparrowhawk | *Falconiformes* | *Accipitridae* | *Accipiter* | *Accipiter gularis* | 217780 | 154687 |
| SRX7544544 | Wild Animal First Aid Center of Sangzhi County, Hunan | Wild | 15 | Whitebellygoldenpheasant | *Galliformes* | *Phasianidae* | *Chrysolophus* | *Chrysolophus amherstiae* | 8530740 | 5775896 |
| SRX7544810 | Farms in Xupu County, Hunan | Wild | 15 | Silver Pheasant | *Galliformes* | *Phasianidae* | *Lophura* | *Lophura nycthemera* | 12,583,524 | 9,925,386 |
| SRX7543531 | HaZ Zoo, Zhejiang | Breeding | 17 | Reeves's Pheasant | *galliformes* | *Phasianidae* | *Syrmaticus* | *Syrmaticus reevesii* | 903294 | 556174 |
| SRX7545232 | Farm in Sangzhi County, Hunan | Breeding | 16 | Silver pheasant | *Galliformes* | *Phasianidae* | *Lophura* | *Lophura nycthemera* | 13909252 | 9996548 |
| SRX7544547 | Wild Animal First Aid Center of Sangzhi County, Hunan | Wild | 15 | Golden Pheasant | *Galliformes* | *Phasianidae* | *Chrysolophus* | *Chrysolophus pictus* | 17679640 | 14668562 |
| SRX7544549 | Farm in Sangzhi County, Hunan | Breeding | 17 | Golden Pheasant | *Galliformes* | *Phasianidae* | *Chrysolophus* | *Chrysolophus pictus* | 1512304 | 886542 |
| SRX7543504 | HEB Zoo, Heilongjiang | Breeding | 12 | Guinea fowl | *Galliformes* | *Numididae* | *Numida* | *Numida meleagris* | 1992466 | 1654233 |
| SRX7543519 | HEB Zoo, Heilongjiang | Breeding | 11 | Guinea fowl | *Galliformes* | *Numididae* | *Numida* | *Numida meleagris* | 391740 | 298652 |
| SRX7543663 | Farm, Hunan | Breeding | 15 | Yellow-bellied Tragopan | *Galliformes* | *Phasianidae* | *Tragopan* | *Tragopan caboti* | 6654698 | 4492101 |
| SRX7543630 | HaZ Zoo, Zhejiang | Breeding | 21 | Blue Peacock | *Galliformes* | *Phasianidae* | *Pavo* | *Pavo cristatus* | 4027326 | 3065423 |
| SRX7543814 | Farms in Xupu County, Hunan | Breeding | 20 | Blue peacock | *Galliformes* | *Phasianidae* | *Pavo* | *Pavo cristatus* | 4982618 | 3126354 |
| SRX7543674 | Farms in Xupu County, Hunan | Breeding | 20 | Blue peacock | *Galliformes* | *Phasianidae* | *Pavo* | *Pavo cristatus* | 7475376 | 4652324 |
| SRX7544864 | Farms in Xupu County, Hunan | Breeding | 15 | Blue Peacock | *Galliformes* | *Phasianidae* | *Pavo* | *Pavo cristatus* | 16916018 | 11662354 |
| SRX7544539 | MES Mountain, Heilongjiang | Wild | 18 | Reeves's Pheasant | *Galliforms* | *Phasianidae* | *Syrmaticus* | *Syrmaticus reevesii* | 1692230 | 1336541 |
| SRX7544127 | HEB Zoo, Heilongjiang | Breeding | 5 | Blue peacock | *Galliforms* | *Phasianidae* | *Pavo* | *Pavo cristatus* | 1087916 | 884210 |
| SRX7543507 | HEB Zoo, Heilongjiang | Breeding | 13 | Crane | *Gruiformes* | *Gruidae* | *NA* | *NA* | 213436 | 165432 |
| SRX7543503 | HEB Zoo, Heilongjiang | Breeding | 13 | Crane | *Gruiformes* | *Gruidae* | *NA* | *NA* | 578870 | 442136 |
| SRX7543498 | HEB Zoo, Heilongjiang | Breeding | 13 | Great Bustard | *Gruiformes* | *Otididae* | *Otis* | *Otis tarda* | 49682 | 32790 |
| SRX7543629 | HEB Zoo, Heilongjiang | Breeding | 5 | White-naped Crane | *Gruiformes* | *Gruidae* | *Grus* | *Grus vipio* | 58382 | 46128 |
| SRX7543643 | DaL Zoo, Liaoning | Breeding | 9 | White-naped Crane | *Gruiformes* | *Gruidae* | *Grus* | *Grus vipio* | 441220 | 352458 |
| SRX7543777 | DaL Zoo, Liaoning | Breeding | 10 | White-naped Crane | *Gruiformes* | *Gruidae* | *Grus* | *Grus vipio* | 10276032 | 7213652 |
| SRX7543782 | DaL Zoo, Liaoning | Breeding | 9 | Black Crowned-crane | *Gruiformes* | *Gruidae* | *Grus* | *Balearica pavonina* | 226836 | 167825 |
| SRX7543788 | DaL Zoo, Liaoning | Breeding | 10 | Common Crane | *Gruiformes* | *Gruidae* | *Grus* | *Grus grus* | 5889320 | 4321658 |
| SRX7543887 | DaL Zoo, Liaoning | Breeding | 7 | Red-crowned Crane | *Gruiformes* | *Gruidae* | *Grus* | *Grus japonensis* | 14929364 | 9032158 |
| SRX7543973 | DaL Zoo, Liaoning | Breeding | 13 | Hooded Crane | *Gruiformes* | *Gruidae* | *Grus* | *Grus monacha* | 11166388 | 7905487 |
| SRX7544012 | HaZ Zoo, Zhejiang | Breeding | 15 | Red-crowned Crane | *Gruiformes* | *Gruidae* | *Grus* | *Grus japonensis* | 2402892 | 1773265 |
| SRX7543785 | Doumuhu Lake, Hunan | Wild | 16 | Black Swan | *Anseriformes* | *Anatidae* | *Cygnus* | *Cygnus atratus* | 6541322 | 4632512 |
| SRX7544933 | MES Mountain, Heilongjiang | Wild | 19 | Yellow-throated Bunting | *Passeriformes* | *Emberizidae* | *Emberiza* | *Emberiza elegans* | 170252 | 103651 |
| SRX7544934 | MES Mountain, Heilongjiang | Wild | 18 | Yellow-throated Bunting | *Passeriformes* | *Emberizidae* | *Emberiza* | *Emberiza elegans* | 160568 | 112358 |
| SRX7544598 | Xinqing bird banding station, Heilongjiang | Wild | 10 | Yellow-throated Bunting | *Passeriformes* | *Emberizidae* | *Emberiza* | *Emberiza elegans* | 227460 | 157469 |
| SRX7544600 | Changbaishan Mountain, Jilin | Wild | 13 | Yellow-throated Bunting | *Passeriformes* | *Emberizidae* | *Emberiza* | *Emberiza elegans* | 2428876 | 1803058 |
| SRX7544596 | MES Mountain, Heilongjiang | Wild | 14 | Yellow-rumped Flycatcher | *Passeriformes* | *Muscicapidae* | *Ficedula* | *Ficedula zanthopygia* | 326512 | 236548 |
| SRX7544595 | Changbaishan Mountain, Jilin | Wild | 16 | Yellow-browed Willow Warbler | *Passeriformes* | *Sylviidae* | *Phylloscopus* | *Phylloscopus inornatus* | 2130046 | 1633254 |
| SRX7544592 | Changbaishan Mountain, Jilin | Wild | 13 | Yellow-browed Willow Warbler | *Passeriformes* | *Sylviidae* | *Phylloscopus* | *Phylloscopus inornatus* | 1406024 | 1025648 |
| SRX7544584 | MES Mountain, Heilongjiang | Wild | 20 | Yellow-browed Willow Warbler | *Passeriformes* | *Sylviidae* | *Phylloscopus* | *Phylloscopus inornatus* | 893930 | 632547 |
| SRX7544581 | MES Mountain, Heilongjiang | Wild | 20 | Yellow-browed Willow Warbler | *Passeriformes* | *Sylviidae* | *Phylloscopus* | *Phylloscopus inornatus* | 1950258 | 1325487 |
| SRX7544566 | Xinqing bird banding station, Heilongjiang | Wild | 20 | Yellow-browed Bunting | *Passeriformes* | *Emberizidae* | *Emberiza* | *Emberiza chrysophrys* | 156942 | 121654 |
| SRX7544579 | MES Mountain, Heilongjiang | Wild | 12 | Yellow-browed Bunting | *Passeriformes* | *Emberizidae* | *Emberiza* | *Emberiza chrysophrys* | 956508 | 671295 |
| SRX7544570 | MES Mountain, Heilongjiang | Wild | 10 | Yellow-browed Bunting | *Passeriformes* | *Emberizidae* | *Emberiza* | *Emberiza chrysophrys* | 1038822 | 675623 |
| SRX7543749 | MES Mountain, Heilongjiang | Wild | 19 | Trick-billed Willow Warbler | *Passeriformes* | *Sylviidae* | *Phylloscopus* | *Phylloscopus schwarzi* | 2779962 | 1734775 |
| SRX7543231 | MES Mountain, Heilongjiang | Wild | 9 | Trick-billed Willow Warbler | *Passeriformes* | *Sylviidae* | *Phylloscopus* | *Phylloscopus schwarzi* | 352912 | 202922 |
| SRX7543489 | MES Mountain, Heilongjiang | Wild | 11 | Pale-legged Leaf-warbler | *Passeriformes* | *Muscicapidae* | *Phylloscopus* | *Phylloscopus tenellipes* | 143018 | 90392 |
| SRX7544548 | MES Mountain, Heilongjiang | Wild | 11 | Yellow-browed Willow Warbler | *Passeriformes* | *Sylviidae* | *Phylloscopus* | *Phylloscopus inornatus* | 8009638 | 5986361 |
| SRX7551021 | MES Mountain, Heilongjiang | Wild | 16 | Yellow-browed Willow Warbler | *Passeriformes* | *Sylviidae* | *Phylloscopus* | *Phylloscopus inornatus* | 5344726 | 4127519 |
| SRX7545202 | MES Mountain, Heilongjiang | Wild | 11 | Grey Wagtail | *Passeriformes* | *Motacillidae* | *Motacilla* | *Motacilla cinerea* | 929148 | 673238 |
| SRX7545082 | Xinqing bird banding station, Heilongjiang | Wild | 9 | white wagtail | *Passeriformes* | *Motacillidae* | *Motacilla* | *Motacilla alba* | 1188330 | 704298 |
| SRX7545410 | MES Mountain, Heilongjiang | Wild | 6 | Grey Wagtail | *Passeriformes* | *Motacillidae* | *Motacilla* | *Motacilla cinerea* | 4910812 | 3941136 |
| SRX7545066 | MES Mountain, Heilongjiang | Wild | 16 | Tristrams Bunting | *Passeriformes* | *Emberizidae* | *Emberiza* | *Emberiza tristrami* | 837958 | 653052 |
| SRX7544865 | MES Mountain, Heilongjiang | Wild | 19 | Great Tit | *Passeriformes* | *Paridae* | *Parus* | *Parus major* | 2163200 | 1727712 |
| SRX7545034 | MES Mountain, Heilongjiang | Wild | 6 | tomtit | *Passeriformes* | *Paridae* | *Parus spilonotus* | *Parus major* | 12308642 | 7123704 |
| SRX7544823 | MES Mountain, Heilongjiang | Wild | 20 | Marsh Tit | *Passeriformes* | *Paridae* | *Parus* | *Poecile palustris* | 1093620 | 821789 |
| SRX7544809 | MES Mountain, Heilongjiang | Wild | 19 | Long-tailed Tit | *Passeriformes* | *Paridae* | *Aegithalos* | *Aegithalos caudatus* | 156902 | 113555 |
| SRX7544556 | MES Mountain, Heilongjiang | Wild | 14 | Pale Thrush | *Passeriformes* | *Turdidae* | *Turdus* | *Turdus pallidus* | 438694 | 309538 |
| SRX7544554 | MES Mountain, Heilongjiang | Wild | 14 | Pale Thrush | *Passeriformes* | *Turdidae* | *Turdus* | *Turdus pallidus* | 1214930 | 881854 |
| SRX7544800 | MES Mountain, Heilongjiang | Wild | 10 | Pale Thrush | *Passeriformes* | *Turdidae* | *Turdus* | *Turdus pallidus* | 288830 | 210628 |
| SRX7544797 | MES Mountain, Heilongjiang | Wild | 14 | Red tailed thrush | *Passeriformes* | *Turdidae* | *Turdus* | *Turdus naumanni* | 2337142 | 1742514 |
| SRX7544645 | MES Mountain, Heilongjiang | Wild | 12 | Red tailed thrush | *Passeriformes* | *Turdidae* | *Turdus* | *Turdus naumanni* | 529608 | 369541 |
| SRX7544607 | MES Mountain, Heilongjiang | Wild | 13 | Grey-backed Thrush | *Passeriformes* | *Turdidae* | *Turdus* | *Turdus hortulorum* | 2960770 | 2254108 |
| SRX7543520 | Changbaishan Mountain, Jilin | Wild | 18 | Red-rumped Swallow | *Passeriformes* | *Hirundinidae* | *Hirundo* | *Cecropis daurica* | 617370 | 477464 |
| SRX7544109 | MES Mountain, Heilongjiang | Wild | 13 | Common Stonechat | *Passeriformes* | *Muscicapidae* | *Saxicola* | *Saxicola torquata* | 668594 | 461272 |
| SRX7544447 | MES Mountain, Heilongjiang | Wild | 17 | Sparrow | *Passeriformes* | *Passeridae* | *Passer* | *Passer montanus* | 3745450 | 2421997 |
| SRX7544542 | Xinqing bird banding station, Heilongjiang | Wild | 13 | Siskin | *Passeriformes* | *Fringillidae* | *Carduelis* | *Carduelis spinus* | 7013862 | 4729149 |
| SRX7544571 | Xinqing bird banding station, Heilongjiang | Wild | 14 | Siskin | *Passeriformes* | *Fringillidae* | *Carduelis* | *Carduelis spinus* | 10193188 | 6027504 |
| SRX7544582 | MES Mountain, Heilongjiang | Wild | 10 | Siskin | *Passeriformes* | *Fringillidae* | *Carduelis* | *Carduelis spinus* | 144568 | 99415 |
| SRX7544646 | Xinqing bird banding station, Heilongjiang | Wild | 18 | Siberian Rubythroat | *Passeriformes* | *Turdidae* | *Luscinia* | *Luscinia calliope* | 4514600 | 3079636 |
| SRX7544625 | Xinqing bird banding station, Heilongjiang | Wild | 19 | Siberian Rubythroat | *Passeriformes* | *Turdidae* | *Luscinia* | *Luscinia calliope* | 3953168 | 2909091 |
| SRX7544811 | Xinqing bird banding station, Heilongjiang | Wild | 20 | Siberian Rubythroat | *Passeriformes* | *Turdidae* | *Luscinia* | *Luscinia calliope* | 125550 | 88863 |
| SRX7544822 | MES Mountain, Heilongjiang | Wild | 19 | Siberian Rubythroat | *Passeriformes* | *Turdidae* | *Luscinia* | *Luscinia calliope* | 276474 | 192473 |
| SRX7544824 | MES Mountain, Heilongjiang | Wild | 15 | Siberian Blue Robin | *Passeriformes* | *turdidae* | *Luscinia* | *Luscinia cyane* | 274046 | 160870 |
| SRX7544825 | Xinqing bird banding station, Heilongjiang | Wild | 19 | Rustic Bunting | *Passeriformes* | *Emberizidae* | *Emberiza* | *Emberiza rustica* | 136408 | 99029 |
| SRX7544826 | Xinqing bird banding station, Heilongjiang | Wild | 18 | Rustic Bunting | *Passeriformes* | *Emberizidae* | *Emberiza* | *Emberiza rustica* | 171668 | 103301 |
| SRX7544560 | Xinqing bird banding station, Heilongjiang | Wild | 15 | Rustic Bunting | *Passeriformes* | *Emberizidae* | *Emberiza* | *Emberiza rustica* | 141502 | 98391 |
| SRX7544565 | Xinqing bird banding station, Heilongjiang | Wild | 19 | Rustic Bunting | *Passeriformes* | *Emberizidae* | *Emberiza* | *Emberiza rustica* | 97858 | 74586 |
| SRX7544569 | Xinqing bird banding station, Heilongjiang | Wild | 20 | Rustic Bunting | *Passeriformes* | *Emberizidae* | *Emberiza* | *Emberiza rustica* | 210856 | 159165 |
| SRX7544578 | Xinqing bird banding station, Heilongjiang | Wild | 20 | Rustic Bunting | *Passeriformes* | *Emberizidae* | *Emberiza* | *Emberiza rustica* | 273402 | 150445 |
| SRX7544580 | MES Mountain, Heilongjiang | Wild | 12 | Rufous-tailed Robin | *Passeriformes* | *Turdidae* | *Luscinia* | *Luscinia sibilans* | 1524140 | 1085932 |
| SRX7544583 | MES Mountain, Heilongjiang | Wild | 12 | Rufous-tailed Robin | *Passeriformes* | *Turdidae* | *Luscinia* | *Luscinia sibilans* | 2891224 | 1988208 |
| SRX7544591 | Xinqing bird banding station, Heilongjiang | Wild | 13 | Common Rosefinch | *Passeriformes* | *Fringillidae* | *Carpodacus* | *Carpodacus erythrinus* | 402260 | 285492 |
| SRX7544593 | MES Mountain, Heilongjiang | Wild | 15 | Red-flanked Bush Robin | *Passeriformes* | *Muscicapidae* | *Tarsiger* | *Tarsiger cyanurus* | 1961982 | 1594908 |
| SRX7544594 | MES Mountain, Heilongjiang | Wild | 14 | Red-flanked Bush Robin | *Passeriformes* | *Muscicapidae* | *Tarsiger* | *Tarsiger cyanurus* | 1692686 | 1317173 |
| SRX7544597 | MES Mountain, Heilongjiang | Wild | 16 | Rufous-tailed Robin | *Passeriformes* | *Turdidae* | *Luscinia* | *Luscinia sibilans* | 5304232 | 3200793 |
| SRX7544599 | Changbaishan Mountain, Jilin | Wild | 9 | Black-Browed Reed Warbler | *Passeriformes* | *Sylviidae* | *Acrocephalus* | *Acrocephalus bistrigiceps* | 132536 | 88474 |
| SRX7544601 | MES Mountain, Heilongjiang | Wild | 20 | Daurian Redstart | *Passeriformes* | *Turdidae* | *Phoenicurus* | *Phoenicurus auroreus* | 4783668 | 3457221 |
| SRX7544624 | Xinqing bird banding station, Heilongjiang | Wild | 13 | Common redpoll | *Passeriformes* | *Fringillidae* | *Carduelis* | *Carduelis flammea* | 716678 | 493007 |
| SRX7544638 | Changbaishan Mountain, Jilin | Wild | 19 | Red-flanked Bluetail | *Passeriformes* | *Muscicapidae* | *Tarsiger* | *Tarsiger cyanurus* | 1347914 | 849623 |
| SRX7544647 | MES Mountain, Heilongjiang | Wild | 15 | Red-flanked Bluetail | *Passeriformes* | *Muscicapidae* | *Tarsiger* | *Tarsiger cyanurus* | 1867982 | 1532868 |
| SRX7544799 | MES Mountain, Heilongjiang | Wild | 15 | Red-flanked Bluetail | *Passeriformes* | *Muscicapidae* | *Tarsiger* | *Tarsiger cyanurus* | 995062 | 686741 |
| SRX7543230 | Changbaishan Mountain, Jilin | Wild | 20 | Red-flanked Bluetail | *Passeriformes* | *Muscicapidae* | *Tarsiger* | *Tarsiger cyanurus* | 2943832 | 1742929 |
| SRX7543490 | Changbaishan Mountain, Jilin | Wild | 18 | Red-flanked Bluetail | *Passeriformes* | *Muscicapidae* | *Tarsiger* | *Tarsiger cyanurus* | 2430946 | 1804424 |
| SRX7543514 | Changbaishan Mountain, Jilin | Wild | 19 | Red-flanked Bluetail | *Passeriformes* | *Muscicapidae* | *Tarsiger* | *Tarsiger cyanurus* | 2575656 | 1799933 |
| SRX7543528 | Changbaishan Mountain, Jilin | Wild | 7 | Red-breasted Flycatcher | *Passeriformes* | *Muscicapidae* | *Ficedula* | *Ficedula parva* | 12377208 | 7168957 |
| SRX7543567 | Xinqing bird banding station, Heilongjiang | Wild | 18 | Pallas's Rosefinch | *Passeriformes* | *Passeridae* | *Carpodacus* | *Carpodacus roseus* | 1003386 | 692235 |
| SRX7543537 | Xinqing bird banding station, Heilongjiang | Wild | 16 | Pallas's Rosefinch | *Passeriformes* | *Passeridae* | *Carpodacus* | *Carpodacus roseus* | 767926 | 576831 |
| SRX7543533 | MES Mountain, Heilongjiang | Wild | 12 | Pallas's Leaf Warbler | *Passeriformes* | *Sylviidae* | *Phylloscopus* | *Phylloscopus proregulus* | 1772564 | 1369892 |
| SRX7543523 | MES Mountain, Heilongjiang | Wild | 13 | Pallas's Leaf Warbler | *Passeriformes* | *Sylviidae* | *Phylloscopus* | *Phylloscopus proregulus* | 2805252 | 1451466 |
| SRX7543517 | MES Mountain, Heilongjiang | Wild | 11 | Pallas's Leaf Warbler | *Passeriformes* | *Sylviidae* | *Phylloscopus* | *Phylloscopus proregulus* | 422952 | 299148 |
| SRX7543493 | MES Mountain, Heilongjiang | Wild | 13 | Wood Nuthatch | *Passeriformes* | *Sittidae* | *Sitta* | *Sitta europaea* | 2871544 | 1995219 |
| SRX7543486 | MES Mountain, Heilongjiang | Wild | 14 | Wood Nuthatch | *Passeriformes* | *Sittidae* | *Sitta* | *Sitta europaea* | 3713202 | 2850713 |
| SRX7543204 | MES Mountain, Heilongjiang | Wild | 17 | Mugimaki Flycatcher | *Passeriformes* | *Muscicapidae* | *Ficedula* | *Ficedula mugimaki* | 2438264 | 1509254 |
| SRX7543203 | MES Mountain, Heilongjiang | Wild | 18 | Mugimaki Flycatcher | *Passeriformes* | *Muscicapidae* | *Ficedula* | *Ficedula mugimaki* | 2596466 | 1613668 |
| SRX7543201 | MES Mountain, Heilongjiang | Wild | 11 | Azure-winged Magpie | *Passeriformes* | *Corvidae* | *Cyanopica* | *Cyanopica cyanus* | 940022 | 690415 |
| SRX7543632 | Changbaishan Mountain, Jilin | Wild | 18 | Long-tailed Tit | *Passeriformes* | *Paridae* | *Aegithalos* | *Aegithalos caudatus* | 561166 | 350370 |
| SRX7543661 | MES Mountain, Heilongjiang | Wild | 13 | Long-tailed Tit | *Passeriformes* | *Paridae* | *Aegithalos* | *Aegithalos caudatus* | 778718 | 563954 |
| SRX7543673 | MES Mountain, Heilongjiang | Wild | 13 | Long-tailed Tit | *Passeriformes* | *Paridae* | *Aegithalos* | *Aegithalos caudatus* | 2599684 | 1915791 |
| SRX7543685 | Changbaishan Mountain, Jilin | Wild | 13 | Long-tailed Rosefinch | *Passeriformes* | *Passeridae* | *Carpodacus* | *Carpodacus sibiricus* | 108008 | 77285 |
| SRX7543781 | Xinqing bird banding station, Heilongjiang | Wild | 11 | Little Bunting | *Passeriformes* | *Emberizidae* | *Emberiza* | *Emberiza pusilla* | 78098 | 55545 |
| SRX7543783 | Xinqing bird banding station, Heilongjiang | Wild | 20 | Little Bunting | *Passeriformes* | *Emberizidae* | *Emberiza* | *Emberiza pusilla* | 75328 | 49916 |
| SRX7543810 | MES Mountain, Heilongjiang | Wild | 6 | Jay | *Passeriformes* | *Corvidae* | *Garrulu* | *Garrulus glandarius* | 589266 | 358916 |
| SRX7544555 | HaZ Zoo, Zhejiang | Breeding | 14 | Daurian Redstart | *Passeriformes* | *Muscicapidae* | *Phoenicurus* | *Phoenicurus auroreus* | 3840972 | 2735042 |
| SRX7543815 | Xinqing bird banding station, Heilongjiang | Wild | 17 | Hawfinch | *Passeriformes* | *Paridae* | *Coccothraustes* | *Coccothraustes coccothraustes* | 256736 | 199446 |
| SRX7543816 | Xinqing bird banding station, Heilongjiang | Wild | 18 | Hawfinch | *Passeriformes* | *Paridae* | *Coccothraustes* | *Coccothraustes coccothraustes* | 196052 | 119394 |
| SRX7543827 | MES Mountain, Heilongjiang | Wild | 10 | Hawfinch | *Passeriformes* | *Paridae* | *Coccothraustes* | *Coccothraustes coccothraustes* | 270432 | 198485 |
| SRX7544126 | Xinqing bird banding station, Heilongjiang | Wild | 18 | Grosbeak | *Passeriformes* | *Fringilidae* | *Pinicola* | *Pinicola enucleator* | 267836 | 196772 |
| SRX7544446 | MES Mountain, Heilongjiang | Wild | 15 | Grey-backed Thrush | *Passeriformes* | *Muscicapidae* | *Turdus* | *Turdus hortulorum* | 2020508 | 1633535 |
| SRX7544520 | MES Mountain, Heilongjiang | Wild | 15 | Grey-backed Thrush | *Passeriformes* | *Muscicapidae* | *Turdus* | *Turdus hortulorum* | 778236 | 573636 |
| SRX7543635 | Xinqing bird banding station, Heilongjiang | Wild | 5 | Goldcrest | *Passeriformes* | *Muscicapidae* | *Regulus* | *Regulus Goldcrest* | 1146432 | 766645 |
| SRX7543680 | MES Mountain, Heilongjiang | Wild | 16 | Red-breasted Flycatcher | *Passeriformes* | *Muscicapidae* | *Ficedula* | *Ficedula parva* | 7073328 | 4868396 |
| SRX7544128 | MES Mountain, Heilongjiang | Wild | 16 | Eastern Crowned Warble | *Passeriformes* | *Muscicapidae* | *Phylloscopus* | *Phylloscopus coronatus* | 6530588 | 4910188 |
| SRX7543233 | Changbaishan Mountain, Jilin | Wild | 7 | Dusky Warbler | *Passeriformes* | *Sylviidae* | *Phylloscopus* | *Phylloscopus fuscatus* | 769728 | 578020 |
| SRX7543502 | MES Mountain, Heilongjiang | Wild | 8 | Dusky Warbler | *Passeriformes* | *Sylviidae* | *Phylloscopus* | *Phylloscopus fuscatus* | 1575924 | 1340110 |
| SRX7543509 | MES Mountain, Heilongjiang | Wild | 11 | Dusky Warbler | *Passeriformes* | *Sylviidae* | *Phylloscopus* | *Phylloscopus fuscatus* | 366996 | 202217 |
| SRX7543516 | MES Mountain, Heilongjiang | Wild | 12 | Dusky Thrush | *Passeriformes* | *Turdidae* | *Turdus* | *Turdus naumanni* | 523244 | 395341 |
| SRX7543527 | MES Mountain, Heilongjiang | Wild | 12 | Dusky Thrush | *Passeriformes* | *Turdidae* | *Turdus* | *Turdus naumanni* | 2732152 | 1503220 |
| SRX7543586 | Changbaishan Mountain, Jilin | Wild | 16 | Daurian Redstart | *Passeriformes* | *Turdidae* | *Phoenicurus* | *Phoenicurus auroreus* | 1831030 | 1508480 |
| SRX7544016 | Changbaishan Mountain, Jilin | Wild | 16 | Coal Tit | *Passeriformes* | *Paridae* | *Parus spilonotus* | *Periparus ater* | 1595668 | 1153141 |
| SRX7544017 | Changbaishan Mountain, Jilin | Wild | 18 | Coal Tit | *Passeriformes* | *Paridae* | *Parus spilonotus* | *Periparus ater* | 1438222 | 979227 |
| SRX7544018 | Changbaishan Mountain, Jilin | Wild | 17 | Coal Tit | *Passeriformes* | *Paridae* | *Parus spilonotus* | *Periparus ater* | 1059258 | 799110 |
| SRX7543227 | Changbaishan Mountain, Jilin | Wild | 17 | Coal Tit | *Passeriformes* | *Paridae* | *Parus spilonotus* | *Periparus ater* | 134838 | 83993 |
| SRX7543488 | Changbaishan Mountain, Jilin | Wild | 15 | Coal Tit | *Passeriformes* | *Paridae* | *Parus spilonotus* | *Periparus ater* | 189708 | 155207 |
| SRX7543522 | Xinqing bird banding station, Heilongjiang | Wild | 12 | Chestnut-flanked White-eye | *Passeriformes* | *Zosteropidae* | *Zosterops* | *Zosterops erythropleurus* | 672224 | 473668 |
| SRX7543633 | MES Mountain, Heilongjiang | Wild | 17 | Chestnut-flanked White-eye | *Passeriformes* | *Zosteropidae* | *Zosterops* | *Zosterops erythropleurus* | 16887296 | 12145615 |
| SRX7543664 | MES Mountain, Heilongjiang | Wild | 17 | Bunting | *Passeriformes* | *Emberizidae* | *Emberiza* | *Emberiza pusilla* | 151234 | 109814 |
| SRX7543826 | Changbaishan Mountain, Jilin | Wild | 10 | Brown Shrike | *Passeriformes* | *Laniidae* | *Lanius* | *Lanius cristatus* | 10876038 | 7778185 |
| SRX7543707 | MES Mountain, Heilongjiang | Wild | 15 | Brown Shrike | *Passeriformes* | *Laniidae* | *Lanius* | *Lanius cristatus* | 987502 | 681751 |
| SRX7543731 | Xinqing bird banding station, Heilongjiang | Wild | 17 | Brambling | *Passeriformes* | *Passeridae* | *Fringilla* | *Fringilla montifringilla* | 196810 | 139895 |
| SRX7543734 | Xinqing bird banding station, Heilongjiang | Wild | 20 | Brambling | *Passeriformes* | *Passeridae* | *Fringilla* | *Fringilla montifringilla* | 246528 | 172708 |
| SRX7543738 | Xinqing bird banding station, Heilongjiang | Wild | 20 | Brambling | *Passeriformes* | *Passeridae* | *Fringilla* | *Fringilla montifringilla* | 272140 | 199612 |
| SRX7543752 | Xinqing bird banding station, Heilongjiang | Wild | 20 | Brambling | *Passeriformes* | *Passeridae* | *Fringilla* | *Fringilla montifringilla* | 157254 | 113788 |
| SRX7543755 | Xinqing bird banding station, Heilongjiang | Wild | 19 | Brambling | *Passeriformes* | *Passeridae* | *Fringilla* | *Fringilla montifringilla* | 181218 | 129604 |
| SRX7543232 | MES Mountain, Heilongjiang | Wild | 17 | Brambling | *Passeriformes* | *Passeridae* | *Fringilla* | *Fringilla montifringilla* | 1564678 | 1132687 |
| SRX7543518 | Xinqing bird banding station, Heilongjiang | Wild | 15 | Brambling | *Passeriformes* | *Passeridae* | *Fringilla* | *Fringilla montifringilla* | 77478 | 57135 |
| SRX7543787 | Xinqing bird banding station, Heilongjiang | Wild | 8 | Black-tailed Hawfinch | *Passeriformes* | *Passeridae* | *Eophona* | *Eophona migratoria* | 138344 | 94307 |
| SRX7543778 | MES Mountain, Heilongjiang | Wild | 19 | Black-faced Bunting | *Passeriformes* | *Emberizidae* | *Emberiza* | *Emberiza spodocephala* | 644288 | 465230 |
| SRX7543776 | MES Mountain, Heilongjiang | Wild | 20 | Black-faced Bunting | *Passeriformes* | *Emberizidae* | *Emberiza* | *Emberiza spodocephala* | 102118 | 68398 |
| SRX7543772 | Changbaishan Mountain, Jilin | Wild | 18 | Black-faced Bunting | *Passeriformes* | *Emberizidae* | *Emberiza* | *Emberiza spodocephala* | 6987586 | 4811807 |
| SRX7543754 | Xinqing bird banding station, Heilongjiang | Wild | 15 | Black-faced Bunting | *Passeriformes* | *Emberizidae* | *Emberiza* | *Emberiza spodocephala* | 282862 | 196689 |
| SRX7543732 | Xinqing bird banding station, Heilongjiang | Wild | 20 | Black-faced Bunting | *Passeriformes* | *Emberizidae* | *Emberiza* | *Emberiza spodocephala* | 774574 | 561219 |
| SRX7543750 | Changbaishan Mountain, Jilin | Wild | 22 | Black-faced Bunting | *Passeriformes* | *Emberizidae* | *Emberiza* | *Emberiza spodocephala* | 2343070 | 1946426 |
| SRX7543721 | Xinqing bird banding station, Heilongjiang | Wild | 19 | Black-faced Bunting | *Passeriformes* | *Emberizidae* | *Emberiza* | *Emberiza spodocephala* | 1638642 | 1381504 |
| SRX7543713 | Xinqing bird banding station, Heilongjiang | Wild | 20 | Black-faced Bunting | *Passeriformes* | *Emberizidae* | *Emberiza* | *Emberiza spodocephala* | 3994744 | 2936531 |
| SRX7543709 | Xinqing bird banding station, Heilongjiang | Wild | 20 | Black-faced Bunting | *Passeriformes* | *Emberizidae* | *Emberiza* | *Emberiza spodocephala* | 648058 | 407718 |
| SRX7551888 | Xinqing bird banding station, Heilongjiang | Wild | 19 | Black-faced Bunting | *Passeriformes* | *Emberizidae* | *Emberiza* | *Emberiza spodocephala* | 2430342 | 1504026 |
| SRX7543711 | Xinqing bird banding station, Heilongjiang | Wild | 22 | Brown Brow rockpipit | *Passeriformes* | *Prunellidea* | *Prunella* | *Prunella montanella* | 640824 | 492944 |
| SRX7543723 | MES Mountain, Heilongjiang | Wild | 13 | Brown Brow rockpipit | *Passeriformes* | *Prunellidea* | *Prunella* | *Prunella montanella* | 2462800 | 1925448 |
| SRX7543542 | HaZ Zoo, Zhejiang | Breeding | 16 | Lesser Flamingo | *Phoenicopteriformes* | *Phoenicopteridae* | *Phoeniconaias* | *Phoeniconaias minor* | 4370850 | 2384761 |
| SRX7544117 | SHH Wildlife Park, Shanghai | Breeding | 10 | Flamingo | *Phoenicopteriformes* | *Phoenicopteridae* | *Phoenicopterus* | *Phoenicopterus roseus* | 3917872 | 2985796 |
| SRX7544118 | SHH Wildlife Park, Shanghai | Breeding | 10 | Flamingo | *Phoenicopteriformes* | *Phoenicopteridae* | *Phoenicopterus* | *Phoenicopterus roseus* | 4209156 | 2478043 |
| SRX7544120 | SHH Wildlife Park, Shanghai | Breeding | 10 | Flamingo | *Phoenicopteriformes* | *Phoenicopteridae* | *Phoenicopterus* | *Phoenicopterus roseus* | 4361988 | 2278912 |
| SRX7544558 | MES Mountain, Heilongjiang | Wild | 10 | White-backed Woodpecker | *Piciformes* | *Picidae* | *Dendrocopus* | *Dendrocopos leucotos* | 3926222 | 2991307 |
| SRX7544562 | MES Mountain, Heilongjiang | Wild | 13 | Lesser Spotted Woodpecker | *Piciformes* | *Picidae* | *Dendrocopus* | *Dendrocopos minor* | 299312 | 207546 |
| SRX7544559 | MES Mountain, Heilongjiang | Wild | 13 | White-backed Woodpecker | *Piciformes* | *Picidae* | *Dendrocopus* | *Dendrocopos leucotos* | 2356030 | 1854980 |
| SRX7545320 | LZM Zoo, Zhejiang | Breeding | 16 | toucan | *Piciformes* | *Ramphastidae* | *Ramphastos* | *Ramphastos toco* | 2226120 | 1569239 |
| SRX7545231 | LZM Zoo, Zhejiang | Breeding | 19 | toucan | *Piciformes* | *Ramphastidae* | *Ramphastos* | *Ramphastos toco* | 2573710 | 1798649 |
| SRX7543807 | Xinqing bird banding station, Heilongjiang | Wild | 9 | Eurasian wryneck | *Piciformes* | *Turdidae* | *Jynx* | *Jynx torquilla* | 1947648 | 1485448 |
| SRX7544540 | DaL Zoo, Liaoning | Breeding | 10 | Red-breasted Parakeet | *Psittaciformes* | *Psittacidae* | *Psittacula* | *Psittacula alexandri* | 1329670 | 837582 |
| SRX7544541 | LZM Zoo, Zhejiang | Breeding | 12 | Hawk-headed Parrot | *Psittaciformes* | *Psittacidae* | *Eagle head parrot genus* | *Deroptyus accipitrinus* | 67426 | 54501 |
| SRX7544543 | LZM Zoo, Zhejiang | Breeding | 15 | Blue-and-Yellow Macaw | *Psittaciformes* | *Psittacidae* | *Ara* | *Ara ararauna* | 70572 | 56578 |
| SRX7544545 | LZM Zoo, Zhejiang | Breeding | 15 | Blue-throated Macaw | *Psittaciformes* | *Psittacidae* | *Ara* | *Ara glaucogularis* | 106662 | 80397 |
| SRX7544546 | LZM Zoo, Zhejiang | Breeding | 15 | Blue-throated Macaw | *Psittaciformes* | *Psittacidae* | *Ara* | *Ara glaucogularis* | 1542790 | 1218241 |
| SRX7544564 | LZM Zoo, Zhejiang | Breeding | 14 | Mealy Amazon | *Psittaciformes* | *Psittacidae* | *Amazona* | *Amazona farinosa* | 198886 | 141265 |
| SRX7544563 | Suzhou Zoo, Jiangsu | Breeding | 15 | Blue-throated Macaw | *Psittaciformes* | *Psittacidae* | *Ara* | *Ara glaucogularis* | 2914410 | 1993511 |
| SRX7544568 | Suzhou Zoo, Jiangsu | Breeding | 16 | rainbow lorikeet | *Psittaciformes* | *Psittacidae* | *Trichoglossus* | *Trichoglossus moluccanus* | 1728296 | 1340675 |
| SRX7543628 | Suzhou Zoo, Jiangsu | Breeding | 16 | Nanday Conure | *Psittaciformes* | *Psittacidae* | *Aratinga* | *Nandayus nenday* | 3343096 | 2706443 |
| SRX7543571 | HaZ Zoo, Zhejiang | Breeding | 20 | Blue-throated Macaw | *Psittaciformes* | *Psittacidae* | *Ara* | *Ara glaucogularis* | 656390 | 493217 |
