# Supplementary material for: Virome in the cloaca of wild and breeding birds revealed a diversity of significant viruses
Source: Microbiome. 2022 Apr 12;10:60. doi: 10.1186/s40168-022-01246-7 (PMC9001828; doi:10.1186/s40168-022-01246-7)
Supplement: Supplementary file 14 — Additional file 13: Supplementary Table 2. Information of viruses with complete or nearly complete genome identified in cloaca of birds. [file 40168_2022_1246_MOESM13_ESM.docx]

**Supplementary Table 2. Information of viruses with complete or nearly complete genome identified in cloaca of birds.**

| **GenBank No.** | **Virus strain name** | **Library ID** | **Classification: Family/Genus/Species** | **Virus name of the closest relative based on conserved protein** | **Accession numbers of best match** | **Genome Length** | **Virus Reads** | **Median coverage in mapping** | **Identity to known virus based on conserved protein** |
| --- | --- | --- | --- | --- | --- | --- | --- | --- | --- |
| MT138097 | gbk055ade1nc | grosbeak55 | Adenoviridae | Amniota adenovirus 1 | QEJ80751 | 20881 | 18980 | 191.8 | 64.82% |
| MT138101 | war203ade1 | warbler203 | Adenoviridae | Amniota adenovirus 1 | QEJ80751 | 32350 | 3654 | 23.8 | 63.81% |
| MT138103 | wwb174ade01 | willowwarbler174 | Adenoviridae | Amniota adenovirus 1 | QEJ80751 | 32592 | 2672 | 21 | 67.60% |
| MT138098 | par083ade1 | parrot83 | Adenoviridae/Atadenovirus | Psittacine adenovirus 3 | QEJ80726 | 30223 | 11930 | 85 | 74.63% |
| MT138099 | sis058ade1 | siskin58 | Adenoviridae/Aviadenovirus | Southern Psittacara leucophthalmus aviadenovirus | QEJ80767 | 38422 | 87650 | 481.3 | 82.46% |
| MT138100 | thr146ade1nc | thrush146 | Adenoviridae/Aviadenovirus | Southern Psittacara leucophthalmus aviadenovirus | QEJ80767 | 39409 | 4466 | 26 | 78.42% |
| MT138102 | wpk049ade02nc | woodpecker49 | Adenoviridae/Aviadenovirus | Fowl aviadenovirus C | CCE39359 | 19446 | 5994 | 65 | 58.84% |
| MN920663 | spa143ast1 | sparrow143 | Astroviridae/Avastrovirus | Passerine astrovirus 1 | QCO31423 | 6570 | 3428 | 110.1 | 54.10% |
| MN920664 | sru051ast1 | SiberianRubythroat51 | Astroviridae/Avastrovirus | Chicken astrovirus | AFK92936 | 6574 | 2326 | 88 | 37.72% |
| MN920665 | stc111ast1 | stonechat111 | Astroviridae/Avastrovirus | Chicken astrovirus | AEE88305 | 6874 | 8481 | 260.3 | 36.84% |
| MN920666 | tom152ast1 | tomtit152 | Astroviridae/Avastrovirus | Red-necked stint avastrovirus | QDY92319 | 7278 | 6362 | 184.4 | 42.64% |
| MN920667 | tom152ast2 | tomtit152 | Astroviridae/Avastrovirus/Avastrovirus 2 | Wood pigeon astrovirus | CBY02492 | 6510 | 9478 | 310.2 | 48.17% |
| MN920668 | war204ast1 | warbler204 | Astroviridae/Avastrovirus | Chicken astrovirus | AFK92941 | 6848 | 4304 | 132.6 | 47.09% |
| MN920669 | ybb150ast01 | Yellow-browedBunting150 | Astroviridae/Avastrovirus | Passerine astrovirus 4 | QCO31432 | 6816 | 12664 | 392 | 38.40% |
| MN920670 | ybw202ast01 | Yellow-browedWarbler202 | Astroviridae/Avastrovirus | Passerine astrovirus 2 | QCO31426 | 6701 | 5918 | 186.3 | 34.10% |
| MN920671 | ytb089ast01 | Yellow-throatedBunting89 | Astroviridae/Avastrovirus | Passerine astrovirus 1 | QCO31423 | 7172 | 36432 | 1071.8 | 44.72% |
| MN920672 | ytb090ast01 | Yellow-throatedBunting90 | Astroviridae/Avastrovirus | Passerine astrovirus 1 | QCO31423 | 7420 | 1134 | 32.2 | 44.79% |
| MT137991 | bbr144ast1 | BrownBrowrockpipit144 | Astroviridae/Avastrovirus | Passerine astrovirus 3 | QCO31429 | 7060 | 4098 | 122.5 | 39.22% |
| MT137992 | bfb201ast01 | BlackfaceBunting201 | Astroviridae/Avastrovirus | Passerine astrovirus 1 | QCO31423 | 7384 | 96898 | 2768.9 | 44.59% |
| MT137994 | bsk136ast01 | BrownShrike136 | Astroviridae/Avastrovirus | Passerine astrovirus 2 | QCO31426 | 6442 | 3826 | 125.3 | 35.65% |
| MT137995 | bun145astnc1 | Bunting145 | Astroviridae/Avastrovirus | Passerine astrovirus 3 | QCO31429 | 5530 | 2250 | 85.8 | 45.40% |
| MT137996 | cfe153ast01 | Chestnut-flankedWhite-eye153 | Astroviridae/Avastrovirus/Avastrovirus 2 | Wood pigeon astrovirus | CBY02492 | 6963 | 4162 | 126.1 | 52.07% |
| MT137997 | cfe153ast02 | Chestnut-flankedWhite-eye153 | Astroviridae/Avastrovirus | Passerine astrovirus 2 | QCO31426 | 6893 | 20168 | 652.3 | 35.54% |
| MT137999 | coa196ast1 | coaltit196 | Astroviridae/Avastrovirus/Avastrovirus 2 | Wood pigeon astrovirus | CBY02492 | 6801 | 10786 | 334.6 | 48.24% |
| MT138000 | coa196ast2nc | coaltit196 | Astroviridae/Avastrovirus | Red-necked stint avastrovirus | QDY92319 | 7464 | 3204 | 90.6 | 42.18% |
| MT138001 | coa197ast1 | coaltit197 | Astroviridae/Avastrovirus | Red-necked stint avastrovirus | QDY92319 | 7403 | 235790 | 6720.5 | 43.37% |
| MT138002 | cra070ast1 | Crane70 | Astroviridae/Avastrovirus/Avastrovirus 2 | Wood pigeon astrovirus | CBY02492 | 6755 | 834 | 33.6 | 49.21% |
| MT138003 | ecw123ast1 | EasternCrownedWarbler123 | Astroviridae/Avastrovirus | Passerine astrovirus 1 | QCO31423 | 6579 | 4674 | 149.9 | 39.79% |
| MT138005 | hwf041ast1 | hawfinch41 | Astroviridae/Avastrovirus/Avastrovirus 2 | Avastrovirus 2 | AFW05403 | 6943 | 3928 | 119.4 | 49.51% |
| MT138006 | hwf182ast2 | hawfinch182 | Astroviridae/Avastrovirus | Goose astrovirus | YP_009362295 | 7154 | 68156 | 2010.2 | 34.52% |
| MT138007 | ltr178ast1 | long-tailedRosefinch178 | Astroviridae/Avastrovirus/Avastrovirus 2 | Avastrovirus 2 | AFW05403 | 7141 | 5211 | 154 | 49.33% |
| MT138008 | ltt163ast1 | long-tailedtit163 | Astroviridae/Avastrovirus | Chicken astrovirus | AXL64621 | 6760 | 9732 | 303.8 | 34.98% |
| MT138010 | prf037ast2 | PallassRosefinch37 | Astroviridae/Avastrovirus/Avastrovirus 2 | Avastrovirus 2 | AFW05403 | 7468 | 569970 | 16103.9 | 50.32% |
| MT138011 | prf038ast1 | PallassRosefinch38 | Astroviridae/Avastrovirus/Avastrovirus 2 | Avastrovirus 2 | AFW05403 | 6981 | 718270 | 21709.6 | 50.32% |
| MT138012 | rob180ast1nc | robin180 | Astroviridae/Avastrovirus | Chicken astrovirus | AXL64621 | 4277 | 2520 | 124.3 | 34.98% |
| MT138013 | rob181ast1 | robin181 | Astroviridae/Avastrovirus | Chicken astrovirus | AFK92942 | 6294 | 3944 | 142.6 | 36.06% |
| MT138014 | rob181ast2 | robin181 | Astroviridae/Avastrovirus | Chicken astrovirus | AXL64621 | 6330 | 24040 | 801.3 | 34.98% |
| MT138015 | rtr167ast1nc | Rufous-tailedRobin167 | Astroviridae/Avastrovirus | Passerine astrovirus 4 | QCO31432 | 4221 | 2696 | 134.8 | 36.12% |
| MT138016 | rtr168ast1 | Rufous-tailedRobin168 | Astroviridae/Avastrovirus | Astrovirus CDB-2012 | AFF57946 | 6762 | 20786 | 648.6 | 38.88% |
| MT137993 | brb026ast1 | Brambling26 | Astroviridae/Avastrovirus | Red-necked stint avastrovirus | QDY92319 | 6237 | 11544 | 390.5 | 44.11% |
| MT138004 | hftbif16ast1nc | BirdFe016 | Astroviridae/Avastrovirus/Avastrovirus1 | Duck astrovirus CPH | AID55207 | 7044 | 992 | 29.7 | 38.50% |
| MT138009 | prf037ast1nc | PallassRosefinch37 | Astroviridae/Avastrovirus | Red-necked stint avastrovirus | QDY92319 | 6371 | 2038 | 67.5 | 41.18% |
| MT138019 | zftwig05sapo1nc | WildGoose05 | Caliciviridae/Circovirus | Ruddy turnstone calicivirus | QCP68858 | 7669 | 972 | 26.7 | 43.73% |
| MT138020 | hwf182cal1 | hawfinch182 | Caliciviridae/Circovirus | Duck calicivirus | AXF38657 | 8943 | 83290 | 2014.6 | 48.18% |
| MT138021 | mag166cal1 | magpie166 | Caliciviridae/Circovirus | Ruddy turnstone calicivirus | QCP68858 | 8038 | 5349 | 140.4 | 43.37% |
| MT138022 | ybb044cal01 | Yellow-browedBunting44 | Caliciviridae/Circovirus | Ruddy turnstone calicivirus | QCP68858 | 6934 | 1718 | 55.8 | 45.27% |
| MT138023 | fmg067cal1nc | flamingo67 | Caliciviridae/Circovirus | Ruddy turnstone calicivirus | QCP68858 | 7590 | 114156 | 3173.5 | 51.07% |
| MT138024 | res169cal1nc | redstart169 | Caliciviridae/Circovirus | Ruddy turnstone calicivirus B | AXF38728 | 5564 | 6582 | 249.6 | 45.93% |
| MT138025 | bbr034cal1 | BrownBrowrockpipit34 | Caliciviridae/Circovirus | Ruddy turnstone calicivirus | QCP68858 | 8763 | 65122 | 1568 | 42.76% |
| MT138026 | bbr034cal2 | BrownBrowrockpipit34 | Caliciviridae/Circovirus | Ruddy turnstone calicivirus | QCP68858 | 8094 | 72056 | 1878.4 | 42.76% |
| MT138027 | zfwcb08sapo1nc | ftyang08 | Caliciviridae/Circovirus | Ruddy turnstone calicivirus | QCP68858 | 7899 | 2915 | 77.9 | 47.24% |
| MT138028 | xftoti59cal1 | OtistardaFe059 | Caliciviridae/Nacovirus/Duck calicivirus 2 | Duck calicivirus 2 | QEG79148 | 8735 | 1332 | 32.2 | 97.40% |
| MT138017 | cftwhg06cal1 | WhitegeeseFe006 | Caliciviridae/Sanovirus/Duck calicivirus | Duck calicivirus | AXF38657 | 8995 | 469 | 16.2 | 75.19% |
| MT138018 | cftwhg07cal1 | WhitegeeseFe007 | Caliciviridae/Sanovirus/Goose calicivirus | Goose calicivirus | ARM65436 | 7186 | 475 | 13.9 | 65.13% |
| MN928903 | cftbif21cir1 | BirdFe021 | Circoviridae/Circovirus | Goose circovirus | AOS89467 | 1714 | 2915 | 358.8 | 65.28% |
| MN928904 | cftbif23cir1 | BirdFe023 | Circoviridae/Circovirus | Goose circovirus | AOS89467 | 1714 | 7566 | 931.4 | 65.28% |
| MN928905 | dth148cir2 | duskythrush148 | Circoviridae/Circovirus | Bat associated circovirus 1 | YP_009506273 | 2027 | 2178 | 226.7 | 54.04% |
| MN928907 | hftbif16cir2 | BirdFe016 | Circoviridae/Circovirus | Goose circovirus | AOS89467 | 1714 | 5296 | 66.5 | 65.28% |
| MN928908 | hftcra98cir1 | CraneFe098 | Circoviridae/Circovirus | Werosea circovirus | QGR26089 | 1967 | 3610 | 387.2 | 63.18% |
| MN928909 | hftoti50cir1 | OtistardaFe050 | Circoviridae/Circovirus | Werosea circovirus | QGR26089 | 2318 | 3049 | 277.5 | 63.18% |
| MN928910 | par078cir1 | parrot78 | Circoviridae/Circovirus | Beak and feather disease virus | AUL80733 | 2011 | 4068 | 426.8 | 78.84% |
| MN928920 | hftoti46cir1nc | OtistardaFe046 | Circoviridae/Circovirus | Werosea circovirus | QGR26089 | 1712 | 65 | 9.5 | 62.62% |
| MT138041 | wpk049cir02nc | woodpecker49 | Circoviridae/Circovirus | Bat associated circovirus 1 | YP_009506273.1 | 2269 | 120 | 11.2 | 53.33% |
| MT138042 | wpk139cir01 | woodpecker139 | Circoviridae/Circovirus | Bat associated circovirus 1 | YP_009506273.1 | 2025 | 12792 | 1425.8 | 54.04% |
| MT138043 | wpk140cir01nc | woodpecker140 | Circoviridae/Circovirus | Bat associated circovirus 1 | YP_009506273.1 | 1809 | 498 | 58.1 | 53.68% |
| MT138046 | xftoti59cir2 | OtistardaFe059 | Circoviridae/Circovirus | Werosea circovirus | QGR26089.1 | 1967 | 440 | 47.2 | 63.18% |
| MT138048 | zftegr04cir1 | Egret04 | Circoviridae/Circovirus | Werosea circovirus | QGR26089.1 | 1866 | 8230 | 930.6 | 84.92% |
| MT138091 | wftcra58cir1 | CraneFe058 | Circoviridae/Circovirus | Werosea circovirus | QGR26089 | 1967 | 1376 | 147.6 | 63.18% |
| MT138092 | wftcra73cir1 | CraneFe073 | Circoviridae/Circovirus | Werosea circovirus | QGR26089 | 2490 | 59854 | 5643.2 | 63.18% |
| MN928906 | fla07cir3 | Flamingo07 | Circoviridae/Cyclovirus | Circoviridae sp. | AXH74046 | 2332 | 1060 | 95.9 | 54.49% |
| MT138044 | wwb174cir01 | willowwarbler174 | Circoviridae/Cyclovirus | Capybara associated cyclovirus 1 | QFR58251.1 | 1825 | 824 | 95.3 | 52.38% |
| MT138063 | zftwig05cir1 | WildGoose05 | Circoviridae/Cyclovirus | Cyclovirus ZM62 | BAP81883 | 1880 | 120 | 13.5 | 55.09% |
| MT138066 | zfwcb09cir1 | ftyang09 | Circoviridae/Cyclovirus | Bat circovirus | AIF76272 | 1760 | 703 | 84.3 | 86.02% |
| MT138068 | rbu021cir1 | RusticBunting21 | Circoviridae/Cyclovirus | Chicken cyclovirus mg4_1122 | QIR82221 | 1625 | 322 | 41.8 | 57.74% |
| MT138104 | brb027cor1 | Brambling27 | Coronaviridae/Deltacoronavirus | Magpie-robin coronavirus HKU18 | YP_005352853 | 26463 | 127325 | 1015.2 | 81.05% |
| MT138105 | brb028cor1 | Brambling28 | Coronaviridae/Deltacoronavirus | Magpie-robin coronavirus HKU18 | YP_005352853 | 26641 | 93972 | 744.3 | 81.07% |
| MT138106 | dut148cor1 | duskythrush148 | Coronaviridae/Deltacoronavirus/Thrush coronavirus | Thrush coronavirus HKU12-600 | YP_002308496 | 26310 | 8507 | 77.2 | 99.20% |
| MT138107 | lrf178cor1 | long-tailedRosefinch178 | Coronaviridae/Deltacoronavirus | Magpie-robin coronavirus HKU18 | YP_005352853 | 26315 | 1537 | 12.3 | 79.46% |
| MT138108 | rub035cor1 | RusticBunting35 | Coronaviridae/Deltacoronavirus | Magpie-robin coronavirus HKU18 | YP_005352853 | 26528 | 2388 | 19 | 80.79% |
| MT138109 | thr147cor1 | thrush147 | Coronaviridae/Deltacoronavirus | Thrush coronavirus HKU12-600 | YP_002308496 | 26027 | 38348 | 310.9 | 99.11% |
| MN928951 | cftwhg09cir2 | WhitegeeseFe009 | CRESS virus sp. | Thrips-associated genomovirus 2 | YP_009345091 | 1687 | 1773 | 221.8 | 81.90% |
| MN928925 | gps222cir1 | goldenpheasant222 | CRESS virus sp. | Circoviridae sp. | AYP28964 | 2887 | 1220 | 89.2 | 96.25% |
| MN928926 | blp211cre1 | Bluepeacock211 | CRESS virus sp. | Circovirus sp. | QBA83725 | 2620 | 21402 | 1723.6 | 83.50% |
| MN905950 | bbr144dic1nc | BrownBrowrockpipit144 | Dicistroviridae | Aphid lethal paralysis virus | AMH41169 | 9047 | 8666 | 202.1 | 97.66% |
| MN905951 | bfb129dic01 | BlackfaceBunting129 | Dicistroviridae | Rhopalosiphum padi virus | AWK77929 | 9177 | 9818 | 225.7 | 99.14% |
| MN905952 | bfb201dic01nc | BlackfaceBunting201 | Dicistroviridae | Aphid lethal paralysis virus | AUG68733 | 8839 | 56770 | 1425.6 | 97.50% |
| MN905953 | brb030dic1 | Brambling30 | Dicistroviridae | Aphid lethal paralysis virus | APG77969 | 7774 | 1632 | 44.3 | 98.12% |
| MN905954 | brb141dic1 | Brambling141 | Dicistroviridae | Aphid lethal paralysis virus | APG77969 | 9305 | 957804 | 21719.1 | 98.12% |
| MN905955 | brb141dic2 | Brambling141 | Dicistroviridae | Aphid lethal paralysis virus | APG77969 | 8118 | 863958 | 22455.7 | 98.12% |
| MN905956 | brs113dic1 | BrownShrike113 | Dicistroviridae | Bat dicistrovirus | AWU67495 | 9325 | 17552 | 397.2 | 40.28% |
| MN905957 | bsk136dic03 | BrownShrike136 | Dicistroviridae | Kashmir bee virus | QGN03907 | 9118 | 2532 | 66.3 | 83.71% |
| MN905958 | cfe153dic01 | Chestnut-flankedWhite-eye153 | Dicistroviridae | Rhopalosiphum padi virus | AWM98376 | 9557 | 24564 | 542.3 | 99.71% |
| MN905959 | coa130dic1 | coaltit130 | Dicistroviridae | Rhopalosiphum padi virus | AWK77929 | 9127 | 3372 | 78 | 99.14% |
| MN905960 | coa130dic2 | coaltit130 | Dicistroviridae | Aphid lethal paralysis virus | APG77969 | 8499 | 4092 | 101.6 | 98.12% |
| MN905961 | coa196dic1 | coaltit196 | Dicistroviridae | Rhopalosiphum padi virus | AWM98376 | 9333 | 3614 | 81.7 | 99.71% |
| MN905962 | dar170dic1 | DaurianRedstart170 | Dicistroviridae | Aphid lethal paralysis virus | APG77969 | 9345 | 15288 | 345.2 | 98.12% |
| MN905963 | dwb184dic1nc | duskywarbler184 | Dicistroviridae | Aphid lethal paralysis virus | AOZ60528 | 8567 | 14180 | 354.2 | 97.98% |
| MN905964 | fcc172dic4 | Flycatcher172 | Dicistroviridae | Bat dicistrovirus | AWU67495 | 8085 | 15242 | 397.8 | 39.80% |
| MN905965 | jay154dic1 | jay154 | Dicistroviridae | Rhopalosiphum padi virus | AWK77929 | 9418 | 7394 | 165.7 | 99.14% |
| MN905966 | ltt163dic4nc | long-tailedtit163 | Dicistroviridae | Aphid lethal paralysis virus | APG77969 | 8731 | 735819 | 17782.4 | 97.14% |
| MN905967 | mag166dic1nc | magpie166 | Dicistroviridae | Rhopalosiphum padi virus | AWM98376 | 9067 | 1442 | 33.6 | 99.64% |
| MN905968 | muf159dic1 | MugimakiFlycatcher159 | Dicistroviridae | Rhopalosiphum padi virus | AWK77929 | 9433 | 6108 | 136.6 | 99.14% |
| MN905969 | muf160dic3 | MugimakiFlycatcher160 | Dicistroviridae | Bat dicistrovirus | AWU67495 | 8925 | 6780 | 160.3 | 39.07% |
| MN905970 | par076dic1 | parrot76 | Dicistroviridae | Bemisia-associated dicistrovirus 2 | QKY88530 | 9137 | 32598 | 752.8 | 48.29% |
| MN905971 | par078dic1 | parrot78 | Dicistroviridae | Dicistroviridae sp. | AVA30706 | 8216 | 14520 | 372.9 | 45.49% |
| MN905972 | plw155dic6 | PallassLeafWarbler155 | Dicistroviridae | Big Sioux River virus | QGX47956 | 9429 | 51711 | 1157.2 | 42.75% |
| MN905973 | plw155dic7 | PallassLeafWarbler155 | Dicistroviridae | Bat dicistrovirus | AWU67495 | 8833 | 85471 | 2510.6 | 41.82% |
| MN905974 | res169dic1 | redstart169 | Dicistroviridae | Aphid lethal paralysis virus | APG77969 | 9534 | 17006 | 376.4 | 98.50% |
| MN905975 | res169dic2 | redstart169 | Dicistroviridae | Bat dicistrovirus | AWU67495 | 8732 | 31390 | 758.5 | 40.02% |
| MN905976 | rfb200dic2 | Red-flankedBluetail200 | Dicistroviridae | Rhopalosiphum padi virus | AWM98376 | 9165 | 71960 | 1656.7 | 99.66% |
| MN905977 | rob180dic1 | robin180 | Dicistroviridae | Rhopalosiphum padi virus | AWK77929 | 9020 | 3320 | 77.7 | 99.14% |
| MN905978 | rtr167dic2 | Rufous-tailedRobin167 | Dicistroviridae | Rhopalosiphum padi virus | AWK77929 | 9362 | 3796 | 85.6 | 99.14% |
| MN905979 | rtr167dic4 | Rufous-tailedRobin167 | Dicistroviridae | Bat cripavirus | AQP31139 | 9257 | 5566 | 126.9 | 54.77% |
| MN905980 | rtr167dic5 | Rufous-tailedRobin167 | Dicistroviridae | Bat dicistrovirus | AWU67495 | 8940 | 662104 | 15626.8 | 40.02% |
| MN905981 | rwa120dic2 | reedwarbler120 | Dicistroviridae | Bat cripavirus | AQP31139 | 8735 | 59808 | 1444.7 | 54.77% |
| MN905982 | sbr121dic1 | SiberianBlueRobin121 | Dicistroviridae | Aphid lethal paralysis virus | APG77969 | 8446 | 4570 | 114.2 | 98.12% |
| MN905983 | spa143dic2nc | sparrow143 | Dicistroviridae | Mosquito dicistrovirus | YP_009315871 | 8079 | 3264 | 85.2 | 93.53% |
| MN905984 | spa143dic3 | sparrow143 | Dicistroviridae | Robinvale bee virus 3 | AWK77879 | 8562 | 12384 | 321.7 | 59.55% |
| MN905985 | sru050dic1 | SiberianRubythroat50 | Dicistroviridae | Bat dicistrovirus | AWU67495 | 9351 | 18770 | 423.5 | 40.02% |
| MN905986 | stc111dic1nc | stonechat111 | Dicistroviridae | Kashmir bee virus | QGN03907 | 9395 | 11001 | 247.1 | 83.79% |
| MN905987 | stc111dic2 | stonechat111 | Dicistroviridae | Bat dicistrovirus | AWU67495 | 9270 | 11737 | 267.2 | 46.54% |
| MN905988 | swa134dic1 | swallow134 | Dicistroviridae | Aphid lethal paralysis virus | APG77969 | 8567 | 28640 | 705.4 | 98.00% |
| MN905989 | thr095dic1 | thrush95 | Dicistroviridae | Aphid lethal paralysis virus | APG77969 | 9204 | 9536 | 218.6 | 98.00% |
| MN905990 | thr095dic2 | thrush95 | Dicistroviridae | Bat dicistrovirus | AWU67495 | 9528 | 18946 | 421.6 | 40.02% |
| MN905991 | tit100dic1 | tit100 | Dicistroviridae | Aphid lethal paralysis virus | APG77969 | 8572 | 4144 | 102 | 98.12% |
| MN905992 | tit162dic1 | tit162 | Dicistroviridae | Rhopalosiphum padi virus | AWM98376 | 9214 | 77834 | 1782.4 | 99.71% |
| MN905993 | tit162dic3 | tit162 | Dicistroviridae | Aphid lethal paralysis virus | AOZ60528 | 9526 | 133982 | 2967.7 | 98.50% |
| MN905994 | tit162dic4 | tit162 | Dicistroviridae | Aphid lethal paralysis virus | AOZ60528 | 9487 | 72980 | 1623.1 | 98.50% |
| MN905995 | tom099dic1 | tomtit99 | Dicistroviridae | Aphid lethal paralysis virus | APG77969 | 9561 | 21710 | 479.1 | 98.12% |
| MN905996 | wag054dic1 | wagtail54 | Dicistroviridae | Drosophila C virus | QEQ50984 | 9236 | 687342 | 15702.6 | 68.54% |
| MN905997 | wag054dic2 | wagtail54 | Dicistroviridae | Rhopalosiphum padi virus | AWK77929 | 9211 | 1442 | 33 | 99.14% |
| MN905998 | wag88dic1 | wagtail88 | Dicistroviridae | Rhopalosiphum padi virus | AWM98376 | 8999 | 18086 | 424.1 | 99.71% |
| MN905999 | wag171dic3 | wagtail171 | Dicistroviridae | Drosophila C virus | QEQ50982 | 8752 | 5714 | 137.8 | 74.51% |
| MN906000 | wpk049dic02 | woodpecker49 | Dicistroviridae | Rhopalosiphum padi virus | AWK77929 | 9224 | 9376 | 214.5 | 99.14% |
| MN906001 | wpk139dic02 | woodpecker139 | Dicistroviridae | Picornavirales sp. | QDH91478 | 8388 | 18964 | 475 | 34.58% |
| MN906002 | ybb044dic01 | Yellow-browedBunting44 | Dicistroviridae | Rhopalosiphum padi virus | AWK77929 | 9426 | 19382 | 433.9 | 99.14% |
| MN906003 | ybb150dic02 | Yellow-browedBunting150 | Dicistroviridae | Formica exsecta virus 1 | YP_008888536 | 9544 | 105142 | 2324.5 | 94.15% |
| MN906004 | ybw115dic02 | Yellow-browedWarbler115 | Dicistroviridae | Rhopalosiphum padi virus | AWK77929 | 8566 | 18064 | 445 | 99.14% |
| MN906005 | ybw116dic01 | Yellow-browedWarbler116 | Dicistroviridae | Rhopalosiphum padi virus | AWK77929 | 9521 | 19642 | 435.3 | 99.14% |
| MN906006 | ybw132dic02 | Yellow-browedWarbler132 | Dicistroviridae | Aphid lethal paralysis virus | APG77969 | 9303 | 1926 | 44.6 | 97.87% |
| MN906007 | yrf108dic02 | Yellow-rumpedFlycatcher108 | Dicistroviridae | Rhopalosiphum padi virus | AWK77929.1 | 9256 | 7336 | 167.2 | 99.00% |
| MN917661 | gbt105shi2 | Grey-backedThrush105 | Dicistroviridae | Hubei orthoptera virus 1 | YP_009336557.1 | 9267 | 30312 | 690.2 | 54.76% |
| MN917662 | thr095shi1 | thrush95 | Dicistroviridae | Hubei orthoptera virus 1 | YP_009336557.1 | 8802 | 21528 | 516.1 | 54.55% |
| MN917663 | jyt032shi3 | jynxtorquilla32 | Dicistroviridae | Bat cripavirus | AQP31138.1 | 8395 | 1366725 | 34351.3 | 71.13% |
| MN917664 | rbu035shi1 | RusticBunting35 | Dicistroviridae | Bat cripavirus | AQP31138.1 | 9609 | 97440 | 2139.6 | 69.07% |
| MN917665 | rfb093shi2 | Red-flankedBluetail93 | Dicistroviridae | Dicistroviridae sp. | QGW36147.1 | 9746 | 17186 | 372.1 | 70.80% |
| MN917666 | rfb094shi1 | Red-flankedBluetail94 | Dicistroviridae | Bat dicistrovirus | AWU67495.1 | 9049 | 10263 | 239.3 | 41.82% |
| MN917667 | rfb200shi5 | Red-flankedBluetail200 | Dicistroviridae | Bat cripavirus | AQP31139.1 | 10100 | 53808 | 1124.1 | 54.25% |
| MN917669 | wag054shi2 | wagtail54 | Dicistroviridae | Warroolaba Creek virus 2 | QIJ25856.1 | 9593 | 694572 | 15277.3 | 68.35% |
| MN917670 | wpk049shi01 | woodpecker49 | Dicistroviridae | Bat cripavirus | AQP31138.1 | 9006 | 18476 | 445.8 | 64.09% |
| MN917671 | wwb174shi03 | willowwarbler174 | Dicistroviridae | Solenopsis invicta virus 9 | QBL75897.1 | 6465 | 9436 | 308 | 69.54% |
| MN918732 | bfb097dic01 | BlackfaceBunting97 | Dicistroviridae | Aphid lethal paralysis virus | AMH41169.1 | 9594 | 12170 | 267.7 | 97.87% |
| MN918733 | coa195dic1 | coaltit195 | Dicistroviridae | Rhopalosiphum padi virus | AWK77929.1 | 9753 | 7912 | 171.2 | 99.14% |
| MN918734 | coa195dic2 | coaltit195 | Dicistroviridae | Aphid lethal paralysis virus | APG77969.1 | 9813 | 23882 | 513.5 | 98.50% |
| MN918735 | coa197dic1 | coaltit197 | Dicistroviridae | Rhopalosiphum padi virus | AWK77929.1 | 10030 | 67236 | 1414.4 | 99.14% |
| MN918736 | ecw123dic1 | EasternCrownedWarbler123 | Dicistroviridae | Aphid lethal paralysis virus | AMH41169.1 | 9767 | 2759012 | 59603.9 | 97.87% |
| MN918737 | fcc172dic1 | Flycatcher172 | Dicistroviridae | Rhopalosiphum padi virus | AWK77929.1 | 10165 | 29308 | 678.5 | 99.14% |
| MN918738 | fcc172dic2 | Flycatcher172 | Dicistroviridae | Aphid lethal paralysis virus | AMH41169.1 | 9614 | 57592 | 1264 | 97.87% |
| MN918739 | gbt104dic1 | Grey-backedThrush104 | Dicistroviridae | Aphid lethal paralysis virus | APG77969.1 | 9584 | 19798 | 435.9 | 98.00% |
| MN918740 | gbt105dic1 | Grey-backedThrush105 | Dicistroviridae | Bat dicistrovirus | AWU67495.1 | 9748 | 118724 | 2569.8 | 40.02% |
| MN918741 | gbt105dic2 | Grey-backedThrush105 | Dicistroviridae | Aphid lethal paralysis virus | AMH41169.1 | 9633 | 33770 | 739.7 | 97.87% |
| MN918742 | jyt032dic1 | jynxtorquilla32 | Dicistroviridae | Kashmir bee virus | QGN03907.1 | 9905 | 22024 | 469.2 | 83.59% |
| MN917678 | jyt032shi5 | jynxtorquilla32 | unclassified Riboviria | Changjiang picorna-like virus 17 | APG79015.1 | 6454 | 3254 | 106.4 | 80.79% |
| MN918743 | ltt163dic1 | long-tailedtit163 | Dicistroviridae | Rhopalosiphum padi virus | AWM98376.1 | 10127 | 323229 | 6734.6 | 99.86% |
| MN918744 | ltt164dic1 | long-tailedtit164 | Dicistroviridae | Rhopalosiphum padi virus | AWM98376.1 | 10183 | 214401 | 4442.6 | 99.73% |
| MN918745 | ltt164dic2 | long-tailedtit164 | Dicistroviridae | Aphid lethal paralysis virus | AUG68733.1 | 9674 | 110267 | 2455 | 98.27% |
| MN918746 | ltt164dic3 | long-tailedtit164 | Dicistroviridae | Rhopalosiphum padi virus | AWK77929 | 9914 | 214401 | 4563.1 | 99.14% |
| MN918747 | ltt192dic1 | long-tailedTit192 | Dicistroviridae | Rhopalosiphum padi virus | AWK77929 | 9782 | 202007 | 4357.3 | 99.14% |
| MN918748 | ltt192dic2 | long-tailedTit192 | Dicistroviridae | Aphid lethal paralysis virus | APG77969 | 9628 | 15105 | 331 | 98.12% |
| MN918749 | muf159dic2 | MugimakiFlycatcher159 | Dicistroviridae | Aphid lethal paralysis virus | APG77969 | 9822 | 241609 | 5190.3 | 98.25% |
| MN918750 | muf160dic1 | MugimakiFlycatcher160 | Dicistroviridae | Rhopalosiphum padi virus | AWK77929 | 10077 | 50469 | 1056.8 | 99.14% |
| MN918751 | muf160dic2 | MugimakiFlycatcher160 | Dicistroviridae | Aphid lethal paralysis virus | APG77969 | 9681 | 439029 | 9568.8 | 98.00% |
| MN918752 | nut157dic1 | nuthatch157 | Dicistroviridae | Rhopalosiphum padi virus | AWK77929 | 9585 | 7097 | 256.9 | 99.28% |
| MN918753 | plw155dic1 | PallassLeafWarbler155 | Dicistroviridae | Rhopalosiphum padi virus | AWK77929 | 9819 | 755352 | 16231.7 | 99.14% |
| MN918754 | plw156dic1 | PallassLeafWarbler156 | Dicistroviridae | Rhopalosiphum padi virus | AWK77929 | 9954 | 341616 | 7241.4 | 99.14% |
| MN918755 | rfb093dic1 | Red-flankedBluetail93 | Dicistroviridae | Bat cripavirus | AQP31139 | 9965 | 18955 | 401.4 | 54.77% |
| MN918756 | rfb198dic1 | Red-flankedBluetail198 | Dicistroviridae | Aphid lethal paralysis virus | APG77969 | 9635 | 257062 | 5629.5 | 98.12% |
| MN918757 | rfb198dic2 | Red-flankedBluetail198 | Dicistroviridae | Rhopalosiphum padi virus | AWK77929 | 9770 | 133168 | 2876 | 99.14% |
| MN918758 | rfb198dic3 | Red-flankedBluetail198 | Dicistroviridae | Bat cripavirus | AQP31139 | 10092 | 6152 | 128.6 | 54.25% |
| MN918759 | rfb199dic1 | Red-flankedBluetail199 | Dicistroviridae | Kashmir bee virus | QGN03907 | 9936 | 336616 | 7148.3 | 84.06% |
| MN918760 | rfb200dic3 | Red-flankedBluetail200 | Dicistroviridae | Aphid lethal paralysis virus | APG77969 | 9632 | 343464 | 7854 | 98.12% |
| MN918761 | rob181dic1 | robin181 | Dicistroviridae | Aphid lethal paralysis virus | APG77969 | 10886 | 114076 | 2211.1 | 98.12% |
| MN918763 | sru103dic1 | SiberianRubythroat103 | Dicistroviridae | Bat cripavirus | AQP31139 | 10201 | 42750 | 884.3 | 54.77% |
| MN918764 | sru103dic2 | SiberianRubythroat103 | Dicistroviridae | Aphid lethal paralysis virus | AMH41169.1 | 9729 | 100652 | 2182.9 | 97.87% |
| MN918765 | trb102dic1 | TristramsBunting102 | Dicistroviridae | Aphid lethal paralysis virus | AMH41169.1 | 9943 | 181972 | 3861.6 | 97.87% |
| MN918766 | wag171dic1 | wagtail171 | Dicistroviridae | Rhopalosiphum padi virus | AWK77929.1 | 10135 | 87678 | 1825.4 | 99.14% |
| MN918767 | wag171dic2 | wagtail171 | Dicistroviridae | Aphid lethal paralysis virus | AUG68733.1 | 10012 | 242188 | 5104 | 98.37% |
| MN918768 | war203dic1 | warbler203 | Dicistroviridae | Aphid lethal paralysis virus | AMH41169.1 | 9681 | 820506 | 17883.1 | 97.87% |
| MN918769 | war204dic1 | warbler204 | Dicistroviridae | Aphid lethal paralysis virus | APG77969.1 | 10257 | 2367882 | 48710.5 | 98.00% |
| MN918770 | war204dic4 | warbler204 | Dicistroviridae | Bat cripavirus | AQP31139.1 | 10055 | 508536 | 10671.4 | 54.25% |
| MN918771 | wiw109dic1 | willowwarbler109 | Dicistroviridae | Aphid lethal paralysis virus | AMH41169.1 | 9668 | 8656 | 198.2 | 97.87% |
| MN918772 | wpk049dic01 | woodpecker49 | Dicistroviridae | Aphid lethal paralysis virus | AMH41169.1 | 9822 | 357588 | 7681.8 | 97.75% |
| MN918773 | wpk049dic03 | woodpecker49 | Dicistroviridae | Bat cripavirus | AQP31139.1 | 10023 | 49400 | 1039.9 | 54.77% |
| MN918774 | wpk139dic01 | woodpecker139 | Dicistroviridae | Bat cripavirus | AWU67495.1 | 9892 | 230388 | 4914.3 | 42.36% |
| MN918775 | wwb174dic01 | willowwarbler174 | Dicistroviridae | Picornavirales Q_sR_OV_023 | ASG92545.1 | 9984 | 10592 | 223.8 | 38.23% |
| MN918776 | ybb150dic01 | Yellow-browedBunting150 | Dicistroviridae | Aphid lethal paralysis virus | AMH41169.1 | 9683 | 29064 | 633.3 | 97.87% |
| MN918777 | ybw115dic01 | Yellow-browedWarbler115 | Dicistroviridae | Aphid lethal paralysis virus | AMH41169.1 | 9587 | 636946 | 14018.5 | 97.87% |
| MN918778 | ybw132dic01 | Yellow-browedWarbler132 | Dicistroviridae | Rhopalosiphum padi virus | AWM98375.1 | 9909 | 7558 | 160.9 | 98.54% |
| MN918779 | ybw202dic01 | Yellow-browedWarbler202 | Dicistroviridae | Rhopalosiphum padi virus | AWM98375.1 | 9738 | 137056 | 2969.7 | 98.54% |
| MN918780 | ybw202dic02 | Yellow-browedWarbler202 | Dicistroviridae | Aphid lethal paralysis virus | APG77440.1 | 9723 | 102484 | 2544 | 98.37% |
| MN918781 | yrf108dic01 | Yellow-rumpedFlycatcher108 | Dicistroviridae | Aphid lethal paralysis virus | AMH41169.1 | 9656 | 188098 | 4110.3 | 97.87% |
| MN928911 | bif24cir1 | BirdFe024 | Genomoviridae | Pacific flying fox faeces associated gemycircularvirus-4 | AMH87679 | 2178 | 926 | 89.7 | 87.76% |
| MN928912 | cftwhg03cir1 | WhitegeeseFe003 | Genomoviridae | Pacific flying fox faeces associated gemycircularvirus-4 | AMH87679 | 2178 | 137 | 13.3 | 87.76% |
| MN928913 | cftwhg07cir1 | WhitegeeseFe007 | Genomoviridae | Pacific flying fox faeces associated gemycircularvirus-4 | AMH87679 | 2178 | 582 | 56.4 | 87.76% |
| MN928914 | cftwhg09cir3 | WhitegeeseFe009 | Genomoviridae | Tortoise genomovirus 23 | QCS37592 | 2180 | 137 | 14.6 | 77.23% |
| MN928915 | hftbif13cir1 | BirdFe013 | Genomoviridae | Pacific flying fox faeces associated gemycircularvirus-4 | AMH87679 | 2178 | 130 | 12.6 | 87.76% |
| MN928916 | hftbif16cir1 | BirdFe016 | Genomoviridae | Pacific flying fox faeces associated gemycircularvirus-4 | AMH87679 | 2178 | 601 | 58.2 | 87.76% |
| MN928917 | hftbif17cir1 | BirdFe017 | Genomoviridae | Pacific flying fox faeces associated gemycircularvirus-4 | AMH87679 | 2178 | 1637 | 158.6 | 87.76% |
| MT138089 | wftbif14cir1 | BirdFe014 | Genomoviridae | Thrips-associated genomovirus 2 | YP_009345091 | 2186 | 2818 | 272 | 87.69% |
| MT138090 | wftbif32cir1 | BirdFe032 | Genomoviridae | Pacific flying fox faeces associated gemycircularvirus-4 | AMH87679 | 2178 | 378 | 36.6 | 87.76% |
| MT138093 | wftcra73cir2 | CraneFe073 | Genomoviridae | Faeces associated gemycircularvirus 19 | YP_009252366 | 2286 | 1308 | 120.7 | 95.26% |
| MT138029 | ltt164hep1 | long-tailedtit164 | Hepe-like virus | Elicom virus 1 | YP_009553584 | 7220 | 17279 | 505 | 51.20% |
| MT138030 | swa134hep1 | swallow134 | Hepe-like virus | Murine feces-associated hepe-like virus | AWB14594 | 7389 | 2157 | 66.2 | 68.42% |
| MT138032 | muf159hep1 | MugimakiFlycatcher159 | Hepe-like virus | Sogatella furcifera hepe-like virus | YP_009553211 | 8524 | 11069 | 274 | 41.10% |
| MT138033 | muf160hep1 | MugimakiFlycatcher160 | Hepe-like virus | Murine feces-associated hepe-like virus | AWB14594 | 8627 | 20058 | 490.6 | 42.60% |
| MT138037 | sbr121hep1 | SiberianBlueRobin121 | Hepe-like virus | Nudaurelia capensis beta virus | NP_048060 | 7082 | 68210 | 2032.2 | 56.47% |
| MT138038 | plw156hep1 | PallassLeafWarbler156 | Hepe-like virus | Bastrovirus Brazil/sewage | ASM79505 | 8438 | 52910 | 1323.1 | 41.13% |
| MT137942 | dwb184ifl1 | duskywarbler184 | Iflaviridae/Iflavirus | Darwin bee virus 2 | AWK77843 | 9838 | 44660 | 957.8 | 35.04% |
| MT137943 | dwb184ifl2 | duskywarbler184 | Iflaviridae/Iflavirus | Darwin bee virus 2 | AWK77843 | 10135 | 51562 | 1073.5 | 37.81% |
| MT137944 | ecw123ifl1 | EasternCrownedWarbler123 | Iflaviridae/Iflavirus | Darwin bee virus 2 | AWK77843 | 9698 | 26636 | 579.5 | 34.23% |
| MT137945 | fcc172ifl1 | Flycatcher172 | Iflaviridae/Iflavirus | Spodoptera exigua iflavirus 2 | YP_009010984.1 | 9308 | 1520054 | 34457.6 | 28.06% |
| MT137946 | gbt104ifl2 | Grey-backedThrush104 | Iflaviridae/Iflavirus | Opsiphanes invirae iflavirus 1 | YP_009165593 | 10136 | 124856 | 2599.1 | 32.63% |
| MT137947 | jay154ifl2 | jay154 | Iflaviridae/Iflavirus | Opsiphanes invirae iflavirus 1 | YP_009165593 | 9507 | 60512 | 1354 | 32.63% |
| MT137948 | ltt163ifl1 | long-tailedtit163 | Iflaviridae/Iflavirus | Opsiphanes invirae iflavirus 1 | YP_009165593 | 9693 | 173094 | 3768 | 32.63% |
| MT137949 | ltt163ifl2 | long-tailedtit163 | Iflaviridae/Iflavirus | Infectious flacherie virus | ACH57393 | 9820 | 4212 | 90.5 | 37.57% |
| MT137950 | mag166ifl1 | magpie166 | Iflaviridae/Iflavirus | Opsiphanes invirae iflavirus 1 | YP_009165593 | 9786 | 541237 | 11669.8 | 32.72% |
| MT137951 | muf159ifl1 | MugimakiFlycatcher159 | Iflaviridae/Iflavirus | Diabrotica virgifera virgifera virus 1 | APF29088 | 8630 | 6703 | 163.9 | 44.40% |
| MT137952 | muf159ifl2 | MugimakiFlycatcher159 | Iflaviridae/Iflavirus | Rolda virus | AOY34458 | 9492 | 193157 | 4293.7 | 27.71% |
| MT137953 | muf159ifl3 | MugimakiFlycatcher159 | Iflaviridae/Iflavirus | Opsiphanes invirae iflavirus 1 | YP_009165593 | 9586 | 38481 | 855 | 32.72% |
| MT137954 | nut158ifl1 | nuthatch158 | Iflaviridae/Iflavirus | Opsiphanes invirae iflavirus 1 | YP_009167345.1 | 9390 | 326132 | 7328.4 | 39.68% |
| MT137956 | plw156ifl1 | PallassLeafWarbler156 | Iflaviridae/Iflavirus | Opsiphanes invirae iflavirus 1 | YP_009165593 | 8950 | 24646 | 581 | 32.67% |
| MT137957 | rfb094ifl1 | Red-flankedBluetail94 | Iflaviridae/Iflavirus | Hubei picorna-like virus | QED42924 | 10183 | 614398 | 12730.8 | 36.88% |
| MT137958 | rfb094ifl2 | Red-flankedBluetail94 | Iflaviridae/Iflavirus | Hubei picorna-like virus | QED42924 | 10519 | 613159 | 12299.3 | 36.50% |
| MT137959 | rfb094ifl3nc | Red-flankedBluetail94 | Iflaviridae/Iflavirus | Opsiphanes invirae iflavirus 1 | YP_009165593 | 9716 | 1582 | 34.4 | 48.82% |
| MT137960 | rfb199ifl1 | Red-flankedBluetail199 | Iflaviridae/Iflavirus | Ectropis obliqua picorna-like virus | NP_919029 | 9183 | 13040 | 299.6 | 87.56% |
| MT137961 | rfb199ifl2nc | Red-flankedBluetail199 | Iflaviridae/Iflavirus | Rolda virus | AOY34458 | 9448 | 361714 | 8078.1 | 27.31% |
| MT137962 | rfb200ifl1 | Red-flankedBluetail200 | Iflaviridae/Iflavirus | Rolda virus | AOY34458 | 10339 | 414454 | 8458.2 | 28.65% |
| MT137963 | rob114Ifl1 | robin114 | Iflaviridae/Iflavirus | Opsiphanes invirae iflavirus 1 | YP_009165593 | 9668 | 38338 | 836.7 | 32.63% |
| MT137964 | rob181Ifl1 | robin181 | Iflaviridae/Iflavirus | Opsiphanes invirae iflavirus 1 | YP_009165593 | 9824 | 226418 | 4898 | 32.63% |
| MT137966 | rtr167Ifl1 | Rufous-tailedRobin167 | Iflaviridae/Iflavirus | Opsiphanes invirae iflavirus 1 | YP_009165593 | 9868 | 322750 | 6901.1 | 32.58% |
| MT137967 | spa143Ifl1 | sparrow143 | Iflaviridae/Iflavirus | Opsiphanes invirae iflavirus 1 | YP_009165593 | 9529 | 10512 | 232.8 | 32.63% |
| MT137968 | spw161Ifl1 | sparrowhawk161 | Iflaviridae/Iflavirus | Opsiphanes invirae iflavirus 1 | YP_009165593 | 9312 | 114778 | 2600.7 | 32.63% |
| MT137969 | sru052Ifl1 | SiberianRubythroat52 | Iflaviridae/Iflavirus | King virus | YP_009315906.1 | 9979 | 8428 | 178.2 | 98.40% |
| MT137970 | thr095Ifl1 | thrush95 | Iflaviridae/Iflavirus | Spodoptera exigua iflavirus 1 | YP_004935363 | 9545 | 3590 | 79.4 | 78.61% |
| MT137971 | thr096Ifl1 | thrush96 | Iflaviridae/Iflavirus | Pityohyphantes rubrofasciatus iflavirus | YP_009351892 | 9850 | 148970 | 3191.1 | 55.43% |
| MT137972 | thr147Ifl1 | thrush147 | Iflaviridae/Iflavirus | Opsiphanes invirae iflavirus 1 | YP_009165593 | 9711 | 21652 | 470.5 | 32.63% |
| MT137973 | thr173Ifl1 | thrush173 | Iflaviridae/Iflavirus | Opsiphanes invirae iflavirus 1 | YP_009167345.1 | 9309 | 21692 | 491.7 | 39.68% |
| MT137974 | tit162Ifl1 | tit162 | Iflaviridae/Iflavirus | Opsiphanes invirae iflavirus 1 | YP_009167345.1 | 9529 | 82572 | 1828.4 | 39.68% |
| MT137975 | tom099Ifl2 | tomtit99 | Iflaviridae/Iflavirus | Opsiphanes invirae iflavirus 1 | YP_009165593 | 8850 | 265440 | 6328.6 | 39.36% |
| MT137976 | tou079ifl1 | toucan79 | Iflaviridae/Iflavirus | King virus | YP_009315906 | 10755 | 24234 | 475.4 | 97.07% |
| MT137977 | wag171ifl1nc | wagtail171 | Iflaviridae/Iflavirus | Laodelphax striatella honeydew virus 1 | YP_009010941 | 10102 | 1882 | 44 | 98.33% |
| MT137978 | wag171ifl2 | wagtail171 | Iflaviridae/Iflavirus | Sacbrood virus | AFQ37841 | 10175 | 165480 | 3431.6 | 32.53% |
| MT137979 | wag171ifl3 | wagtail171 | Iflaviridae/Iflavirus | Hubei picorna-like virus 39 | YP_009336612 | 9695 | 30240 | 658.1 | 32.12% |
| MT137980 | wag171ifl4 | wagtail171 | Iflaviridae/Iflavirus | Opsiphanes invirae iflavirus 1 | YP_009165593 | 9066 | 5318 | 123.8 | 32.63% |
| MT137981 | wag171ifl5 | wagtail171 | Iflaviridae/Iflavirus | Sacbrood virus | AFQ95415 | 9094 | 164714 | 3821.7 | 33.06% |
| MT137982 | war204ifl1 | warbler204 | Iflaviridae/Iflavirus | Opsiphanes invirae iflavirus 1 | YP_009165593 | 10048 | 65222 | 1369.6 | 32.58% |
| MT137983 | war204ifl1nc | warbler204 | Iflaviridae/Iflavirus | Xysticus cristatus iflavirus | APD13905 | 9607 | 1746 | 54 | 37.61% |
| MT137984 | war204ifl2 | warbler204 | Iflaviridae/Iflavirus | Opsiphanes invirae iflavirus 1 | YP_009165593 | 8862 | 89146 | 2122.5 | 30.03% |
| MT137985 | wpk139ifl01 | woodpecker139 | Iflaviridae/Iflavirus | Wuhan coneheads virus 1 | YP_009342053 | 7050 | 7498 | 224.4 | 47.34% |
| MT137986 | wpk139ifl02 | woodpecker139 | Iflaviridae/Iflavirus | Rolda virus | AOY34458 | 9518 | 311062 | 6895.8 | 27.78% |
| MT137988 | ybb117ifl01 | Yellow-browedBunting117 | Iflaviridae/Iflavirus | Darwin bee virus 2 | AWK77843 | 10372 | 450070 | 9155.9 | 39.53% |
| MT137989 | ybb150ifl01 | Yellow-browedBunting150 | Iflaviridae/Iflavirus | Opsiphanes invirae iflavirus 1 | YP_009165593 | 9708 | 4644 | 100.9 | 50.98% |
| MT137990 | ybw202ifl01 | Yellow-browedWarbler202 | Iflaviridae/Iflavirus | Pityohyphantes rubrofasciatus iflavirus | YP_009351892 | 9799 | 34002 | 732.2 | 54.20% |
| MN917672 | fmg067shi1 | flamingo67 | Marnaviridae | Hubei leech virus 4 | APG79016.1 | 8359 | 100280 | 2531.3 | 63.86% |
| MN917673 | fmg067shi2nc | flamingo67 | Marnaviridae | Hubei leech virus 4 | APG79016.1 | 7916 | 8738 | 232.9 | 66.67% |
| MN917674 | swa066shi1 | swan66 | Marnaviridae | Beihai picorna-like virus 15 | YP_009330067.1 | 9213 | 5478 | 125.5 | 56.44% |
| MT138127 | brb141urn1 | Brambling141 | Marnaviridae | Marine RNA virus SF-1 | YP_009666352 | 9008 | 17322 | 405.7 | 82.35% |
| MT138128 | cra070shi4 | Crane70 | Marnaviridae | Ubei picorna-like virus 3 | YP_009345029 | 9591 | 24130 | 530.9 | 80.81% |
| MT138129 | cra070shi5 | Crane70 | Marnaviridae | Ubei picorna-like virus 3 | YP_009345029 | 9159 | 17028 | 392.3 | 67.80% |
| MT138130 | cra070shi6 | Crane70 | Marnaviridae | Hubei leech virus 4 | APG79016 | 8245 | 26214 | 670.8 | 65.25% |
| MT138131 | zftfla01rna3 | Flamingo01 | Marnaviridae | Changjiang crawfish virus 1 | YP_009336771 | 4939 | 1118 | 47.8 | 71.46% |
| MT138132 | zftfla02rna3 | Flamingo02 | Marnaviridae | Robinvale bee virus 9 | AWK77882 | 7707 | 1742 | 47.7 | 62.03% |
| MT138133 | sto073RNA1 | stork73 | Marnaviridae | Marnaviridae sp. | QJI53803 | 9245 | 6762 | 154.3 | 59.81% |
| MT138336 | zftfla02pic4 | Flamingo02 | Marnaviridae | Hubei leech virus 2 | YP_009329961.1 | 5118 | 764 | 33.5 | 93.82% |
| MT138110 | dwb062nod1 | DuskyWarbler62 | Nodaviridae | Lutzomyia nodavirus | AKP18617 | 6084 | 54048 | 1874.4 | 31.20% |
| MT138111 | dwb062nod2 | DuskyWarbler62 | Nodaviridae | Hubei permutotetra-like virus 11 | YP_009337277 | 4970 | 137004 | 5816.5 | 55.22% |
| MT138113 | muf159nod2 | MugimakiFlycatcher159 | Nodaviridae | Nodamura virus | NP_077732 | 3786 | 1175 | 65.5 | 57.14% |
| MT138115 | wpk049nod02 | woodpecker49 | Nodaviridae | Wuhan nodavirus | ABB71128 | 2022 | 70 | 7.3 | 55.59% |
| MT138118 | zftfla02nod1 | Flamingo02 | Nodaviridae | Hubei tombus-like virus 25 | YP_009336611 | 5169 | 1560 | 63.7 | 47.42% |
| MT138223 | bsk136par02 | BrownShrike136 | Parvo-like hybrid virus | Lactuca sativa parvo-like virus | QKK82953 | 3429 | 312 | 21.5 | 64.54% |
| MT138224 | cat223par1 | cabotstragopan223 | Parvo-like hybrid virus | Trichosanthes kirilowii parvo-like virus | QKK82950 | 4117 | 23002 | 1178.9 | 69.74% |
| MT138228 | cftwhg03par1 | WhitegeeseFe003 | Parvoviridae/Aveparvovirus | Red-crowned crane parvovirus | YP_009552126.1 | 4868 | 7782 | 337.3 | 52.54% |
| MT138233 | cra070par1 | Crane70 | Parvo-like hybrid virus | Trichosanthes kirilowii parvo-like virus | QKK82947.1 | 5059 | 70570 | 2943.3 | 77.30% |
| MT138235 | dth149par1 | duskythrush149 | Parvoviridae/Densovirus | Bombus cryptarum densovirus | YP_009552708.1 | 5079 | 173232 | 7196.7 | 34.52% |
| MT138239 | fmg067par1 | flamingo67 | Parvo-like hybrid virus | Trichosanthes kirilowii parvo-like virus | QKK82950.1 | 4684 | 23352 | 1051.9 | 73.08% |
| MT138240 | gct065den1 | goldcrest65 | Parvoviridae/Densovirus | Parus major densovirus | YP_009310053.1 | 5579 | 794488 | 30047.9 | 99.25% |
| MT138244 | hbl169par3 | hornbill69 | Parvo-like hybrid virus | Trichosanthes kirilowii parvo-like virus | QKK82950.1 | 3672 | 12466 | 716.3 | 71.61% |
| MT138245 | hbl169par4 | hornbill69 | Parvo-like hybrid virus | Trichosanthes kirilowii parvo-like virus | QKK82947.1 | 3753 | 2158 | 121.3 | 77.30% |
| MT138250 | hftoti50par1 | OtistardaFe050 | Parvoviridae sp. | Parvoviridae sp. | AUW34317.1 | 4047 | 1622 | 84.6 | 51.49% |
| MT138252 | hwf061par2 | hawfinch61 | Parvoviridae/Densovirus | Parus major densovirus | YP_009310053.1 | 5495 | 2070 | 79.5 | 99.62% |
| MT138256 | ltt163par2 | long-tailedtit163 | Parvoviridae/Densovirus | Culex pipiens densovirus | YP_002887625.1 | 5328 | 3200 | 126.7 | 52.65% |
| MT138262 | muf159pap1 | MugimakiFlycatcher159 | Parvoviridae/Bidnapavovirus | Dog feces bidnaparvovirus | DAC81758.1 | 6576 | 37703 | 1354.2 | 55.81% |
| MT138264 | par076par1 | parrot76 | Parvo-like hybrid virus | Aphanomyces astaci | KAF0703963.1 | 3870 | 43584 | 2376.3 | 58.63% |
| MT138265 | par077par1 | parrot77 | Parvo-like hybrid virus | Parvo-like hybrid virus UC4 | AGV22428.1 | 3824 | 3969 | 219 | 66.67% |
| MT138268 | par081par3 | parrot81 | Parvoviridae/Densovirus | Parus major densovirus | YP_009310053 | 4568 | 311 | 14.4 | 99.25% |
| MT138274 | zftwig01paden2 | WildGoose01 | Parvoviridae/Bidnapavovirus | Dog feces bidnaparvovirus | DAC81758 | 9311 | 113210 | 2565.5 | 70.83% |
| MT138278 | plw118par1 | PallassLeafWarbler118 | Parvoviridae/Monodnaparvovirus | False wolf spider monodnaparvovirus | DAC81438 | 7627 | 357083 | 9878.7 | 43.15% |
| MT138279 | plw156par1 | PallassLeafWarbler156 | Parvoviridae/Monodnaparvovirus | False wolf spider monodnaparvovirus | DAC81438 | 7355 | 93312 | 2676.9 | 43.15% |
| MT138284 | zftwig03adas2nc | WildGoose03 | Parvoviridae/Dependoparvovirus | Adeno-associated virus | QDH44144 | 4229 | 1214 | 60.6 | 39.52% |
| MT138287 | zftfla03par1 | Flamingo03 | Parvoviridae | Abeoforma parvovirus | DAC80331 | 4952 | 1816 | 77.4 | 36.36% |
| MT138289 | rbu019par2 | RusticBunting19 | Parvoviridae/Densovirus | Parus major densovirus | YP_009310053 | 4540 | 488 | 22.7 | 99.62% |
| MT138298 | pst217par01 | pheasant217 | Parvo-like hybrid virus | Forsythia suspensa parvo-like virus | QKK82940 | 3490 | 854 | 51.6 | 89.77% |
| MT138300 | rob180par1 | robin180 | Parvoviridae | False wolf spider monodnaparvovirus-VP | DAC81439 | 7723 | 55268 | 1510 | 32.12% |
| MT138302 | swa134par3 | swallow134 | Parvoviridae/Dependoparvovirus | Muscovy duck parvovirus | ASR91720 | 5081 | 1068 | 45.2 | 51.41% |
| MT138305 | rtr167par2 | Rufous-tailedRobin167 | Parvo-like hybrid virus | Lactuca sativa parvo-like virus | QKK82953 | 3453 | 2908 | 177.7 | 65.69% |
| MT138307 | rtr168par1 | Rufous-tailedRobin168 | Parvoviridae/Densovirus | Bombus cryptarum densovirus | YP_009552708 | 5368 | 242032 | 9513.6 | 34.71% |
| MT138308 | swa066par1 | swan66 | Parvo-like hybrid virus | Parvo-like hybrid virus UC4 | AGV22428 | 3562 | 5162 | 305.8 | 71.43% |
| MT138309 | ybb117par01 | Yellow-browedBunting117 | Parvoviridae/Monodnaparvovirus | False wolf spider monodnaparvovirus | DAC81438 | 6994 | 14224 | 429.1 | 43.15% |
| MT138314 | sis142par1 | siskin142 | Parvoviridae/Dependoparvovirus | Avian adeno-associated virus | QEJ80808 | 5220 | 24962 | 1009 | 70.00% |
| MT138315 | sis142par2 | siskin142 | Parvoviridae/Ambidensovirus | Ambidensovirus sp. | QGJ83204 | 3993 | 482 | 25.5 | 44.69% |
| MT138317 | thr173par1 | thrush173 | Parvoviridae | Desmodus rotundus dependoparvovirus | AVR53758 | 4666 | 3515 | 159 | 37.31% |
| MT138324 | zftwcb10par1 | ftyang10 | Parvoviridae | Abeoforma parvovirus | DAC80331 | 4979 | 1171 | 49.6 | 30.52% |
| MT138327 | zftwig05adas2 | WildGoose05 | Parvoviridae/Dependoparvovirus | Adeno-associated virus | QHD57622 | 4380 | 232 | 11.2 | 61.56% |
| MT138329 | zftwig01par1 | WildGoose01 | Parvoviridae | Ambidensovirus sp. | AWV66983 | 7244 | 1404 | 40.9 | 24.02% |
| MT138330 | zftwig04par3 | WildGoose04 | Parvoviridae/Dependoparvovirus | Adeno-associated virus | QDH44145 | 5039 | 1972 | 82.6 | 39.52% |
| MT138331 | zftwig05par1 | WildGoose05 | Parvoviridae | Mouse kidney parvovirus | YP_009553675 | 4715 | 3552 | 159 | 25.00% |
| MT138332 | zftwig05par2 | WildGoose05 | Parvoviridae | Parvovirus fur seal/ATROP43/BR/2012 | AKI82157 | 4899 | 1688 | 73.5 | 39.00% |
| MT138333 | zftwig05par5 | WildGoose05 | Parvoviridae | Pigeon parvovirus A | AGW95844 | 4947 | 2296 | 97.9 | 27.32% |
| MT138334 | zftwig05par6 | WildGoose05 | Parvoviridae | Human parvovirus 4 | AIC33852 | 7497 | 1296 | 36.5 | 28.12% |
| MT138401 | bth060pas01 | BlackTailedHawFinch60 | Parvoviridae/Chapparvovirus(没树) | Avian chapparvovirus | QKX49056 | 4505 | 1002 | 46.9 | 64.49% |
| MT138402 | bth060pas02 | BlackTailedHawFinch60 | Parvoviridae/Chapparvovirus(没树) | Peafowl parvovirus 1 | QGJ83201 | 4379 | 13588 | 654.7 | 75.73% |
| MT138209 | bfb009ave01 | BlackfaceBunting09 | Parvoviridae/Aveparvovirus | Pileated finch aveparvovirus | AVR53746 | 5208 | 6164 | 249.7 | 65.96% |
| MT138210 | bfb010ave01 | BlackfaceBunting10 | Parvoviridae/Aveparvovirus | Pileated finch aveparvovirus | AVR53746 | 5334 | 280238 | 11085.5 | 65.96% |
| MT138211 | bfb011ave01 | BlackfaceBunting11 | Parvoviridae/Aveparvovirus | Pileated finch aveparvovirus | AVR53746 | 5339 | 9194 | 363.4 | 66.01% |
| MT138212 | bfb012ave01 | BlackfaceBunting12 | Parvoviridae/Aveparvovirus | Pileated finch aveparvovirus | AVR53746 | 6092 | 1137624 | 39402.3 | 66.12% |
| MT138213 | bfb013ave01 | BlackfaceBunting13 | Parvoviridae/Aveparvovirus | Pileated finch aveparvovirus | AVR53746 | 5619 | 439084 | 16488.1 | 65.63% |
| MT138214 | bfb014ave01 | BlackfaceBunting14 | Parvoviridae/Aveparvovirus | Pileated finch aveparvovirus | AVR53746 | 5728 | 7542 | 277.8 | 65.63% |
| MT138215 | bfb098ave01 | BlackfaceBunting98 | Parvoviridae/Aveparvovirus | Pileated finch aveparvovirus | AVR53746 | 5891 | 408524 | 14632.2 | 65.96% |
| MT138216 | bpk075par01 | Bluepeacock75 | Parvoviridae/Aveparvovirus | Pigeon parvovirus A | AGW95844 | 5645 | 12238 | 457.4 | 60.73% |
| MT138241 | gps215par1 | goldenpheasant215 | Parvoviridae/Aveparvovirus | Chicken parvovirus | AYG77878.1 | 5291 | 30206 | 1204.6 | 74.92% |
| MT138263 | par074par1 | parrot74 | Parvoviridae/Aveparvovirus | Pigeon parvovirus A | AGW95844.1 | 5753 | 2309 | 88.5 | 51.02% |
| MT138266 | par081par1 | parrot81 | Parvoviridae/Aveparvovirus | Pigeon parvovirus A | AGW95844.1 | 6015 | 145593 | 5107.3 | 51.58% |
| MT138267 | par081par2 | parrot81 | Parvoviridae/Aveparvovirus | Pigeon parvovirus A | AGW95844 | 5589 | 200 | 7.6 | 51.33% |
| MT138269 | par083par1 | parrot83 | Parvoviridae/Aveparvovirus | Pigeon parvovirus A | AGW95844 | 5916 | 1279111 | 45620.8 | 51.53% |
| MT138271 | par084par1 | parrot84 | Parvoviridae/Aveparvovirus | Pigeon parvovirus A | AGW95844 | 5702 | 14617 | 540.9 | 50.86% |
| MT138272 | par085par2nc | parrot85 | Parvoviridae/Aveparvovirus | Pigeon parvovirus A | AGW95844 | 5375 | 8117 | 318.6 | 50.96% |
| MT138273 | par087par1 | parrot87 | Parvoviridae/Aveparvovirus | Pigeon parvovirus A | AGW95844 | 5684 | 7062 | 262.2 | 50.96% |
| MT138285 | trb102par1nc | TristramsBunting102 | Parvoviridae/Aveparvovirus | Pileated finch aveparvovirus | AVR53746 | 5006 | 488 | 20.6 | 65.79% |
| MT138286 | rbf133par1nc | Red-breastedFlycatcher133 | Parvoviridae/Aveparvovirus | Pileated finch aveparvovirus | AVR53746 | 5093 | 417990 | 17317.1 | 65.79% |
| MT138299 | wwb174par01 | willowwarbler174 | Parvoviridae/Aveparvovirus | Protoparvovirus HK-2014 | AIW53329 | 5056 | 16584 | 692.1 | 53.33% |
| MT138319 | wftcra74par1 | CraneFe074 | Parvoviridae/Aveparvovirus | Red-crowned crane parvovirus | YP_009552825 | 5750 | 5998 | 220.1 | 99.26% |
| MT138253 | lib042par1 | LittleBunting42 | Parvoviridae/Chapparvovirus | Avian chapparvovirus | QKX49056.1 | 4653 | 7497 | 342 | 47.86% |
| MT138254 | lib043par1 | LittleBunting43 | Parvoviridae/Chapparvovirus | Avian chapparvovirus | QKX49056.1 | 4943 | 7098 | 303 | 48.94% |
| MT138257 | ltt163par3 | long-tailedtit163 | Parvoviridae/Chapparvovirus | Avian chapparvovirus | QKX49056.1 | 3389 | 339 | 21.1 | 50.60% |
| MT138270 | par083par2 | parrot83 | Parvoviridae/Chapparvovirus | Peafowl parvovirus 2 | QGJ83204 | 4553 | 2550 | 118.2 | 50.96% |
| MT138281 | rfb199par1 | Red-flankedBluetail199 | Parvoviridae/Chapparvovirus | Peafowl parvovirus 2 | QGJ83204 | 4909 | 5926 | 254.7 | 54.20% |
| MT138290 | rbu018par1 | RusticBunting18 | Parvoviridae/Chapparvovirus | Avian chapparvovirus | QKX49056 | 4359 | 4286 | 207.5 | 48.55% |
| MT138291 | rbu036par2 | RusticBunting36 | Parvoviridae/Chapparvovirus | Peafowl parvovirus 2 | QGJ83204 | 4317 | 1514 | 74 | 44.21% |
| MT138292 | rbu019par1 | RusticBunting19 | Parvoviridae/Chapparvovirus | Parvoviridae sp. | AUW34315 | 4990 | 6826 | 288.6 | 48.08% |
| MT138293 | rbu020par1 | RusticBunting20 | Parvoviridae/Chapparvovirus | Avian chapparvovirus | QKX49056 | 4405 | 4704 | 225.3 | 50.22% |
| MT138294 | rbu036par1 | RusticBunting36 | Parvoviridae/Chapparvovirus | Peafowl parvovirus 2 | QGJ83204 | 4402 | 1036 | 49.7 | 48.80% |
| MT138297 | ytb023par01 | Yellow-throatedBunting23 | Parvoviridae/Chapparvovirus | Peafowl parvovirus 2 | QGJ83204 | 4249 | 140 | 7 | 49.63% |
| MT138303 | swa134par1 | swallow134 | Parvoviridae/Chapparvovirus | Avian chapparvovirus | QKX49056 | 4287 | 4871 | 239.7 | 48.06% |
| MT138310 | ybb150par04 | Yellow-browedBunting150 | Parvoviridae/Chapparvovirus | Chapparvovirus sp. | QID88579 | 4139 | 59842 | 3050.7 | 49.14% |
| MT138311 | ybb150par01 | Yellow-browedBunting150 | Parvoviridae/Chapparvovirus | Psittacara leucophthalmus chapparvovirus | QEJ80805 | 4394 | 3552 | 170.6 | 46.02% |
| MT138318 | wftcra74par2 | CraneFe074 | Parvoviridae/Chapparvovirus | Parvoviridae sp. | AUW34321 | 4263 | 544 | 26.9 | 53.92% |
| MT138320 | wftcra79par2 | CraneFe079 | Parvoviridae/Chapparvovirus | Peafowl parvovirus 2 | QGJ83204 | 4209 | 536 | 26.9 | 45.86% |
| MT138321 | zftegr01par1 | Egret01 | Parvoviridae/Chapparvovirus | Peafowl parvovirus 2 | QGJ83204 | 4290 | 642 | 33.5 | 51.73% |
| MT138325 | zfwcb07par1 | ftyang07 | Parvoviridae/Chapparvovirus | Tasmanian devil-associated chapparvovirus 1 | QBJ04585 | 3527 | 331 | 19.8 | 34.72% |
| MT138217 | brb001par1 | Brambling01 | Parvoviridae/Chapparvovirus | Peafowl parvovirus 2 | QGJ83204 | 4352 | 1148 | 55.7 | 48.52% |
| MT138218 | brb024par1 | Brambling24 | Parvoviridae/Chapparvovirus | Avian chapparvovirus | QKX49056 | 4438 | 1652 | 78.5 | 46.23% |
| MT138219 | brb030par1 | Brambling30 | Parvoviridae/Chapparvovirus | Avian chapparvovirus | QKX49056 | 4838 | 21192 | 924.2 | 45.95% |
| MT138220 | brb030par2 | Brambling30 | Parvoviridae/Chapparvovirus | Avian chapparvovirus | QKX49056 | 4915 | 21428 | 919.9 | 47.13% |
| MT138221 | bs219par01 | blackswan219 | Parvoviridae/Chapparvovirus | Chestnut teal chaphamaparvovirus 3 | QMI57837 | 4427 | 4742 | 226 | 65.53% |
| MT138222 | bsk136par01 | BrownShrike136 | Parvoviridae/Chapparvovirus | Avian chapparvovirus | QKX49056 | 4361 | 1130 | 54.7 | 48.82% |
| MT138229 | cftwhg05par1 | WhitegeeseFe005 | Parvoviridae/Chapparvovirus | Chestnut teal chaphamaparvovirus 1 | QMI57829.1 | 4372 | 399 | 21 | 46.26% |
| MT138230 | cftwhg07par1 | WhitegeeseFe007 | Parvoviridae/Chapparvovirus | Chestnut teal chaphamaparvovirus 1 | QMI57829.1 | 4326 | 851 | 41.5 | 43.38% |
| MT138231 | cftwhg09par1nc | WhitegeeseFe009 | Parvoviridae/Chapparvovirus | Chestnut teal chaphamaparvovirus 2 | QMI57833.1 | 4203 | 1448 | 72.7 | 60.96% |
| MT138234 | dar170par1 | DaurianRedstart170 | Parvoviridae/Chapparvovirus | Parvoviridae sp. | AUW34315.1 | 4706 | 2888 | 154.2 | 47.96% |
| MT138238 | fcc172par1 | Flycatcher172 | Parvoviridae/Chapparvovirus | Parvoviridae sp. | AUW34315.1 | 4944 | 22648 | 966.6 | 48.11% |
| MT138251 | hwf061par1 | hawfinch61 | Parvoviridae/Chapparvovirus | Peafowl parvovirus 2 | QGJ83204.1 | 4389 | 14762 | 709.7 | 51.98% |
| MT138258 | ltt164par1 | long-tailedtit164 | Parvoviridae/Chapparvovirus | Avian chapparvovirus | QKX49056.1 | 4417 | 3903 | 186.4 | 55.33% |
| MT138275 | rfi063par2 | rosefinch63 | Parvoviridae/Chapparvovirus | Peafowl parvovirus 2 | QGJ83204 | 4559 | 84236 | 3898.6 | 47.62% |
| MT138276 | rfi063par1 | rosefinch63 | Parvoviridae/Chapparvovirus | Avian chapparvovirus | QKX49056 | 4668 | 83672 | 3782.1 | 48.60% |
| MT138282 | rfb200par2 | Red-flankedBluetail200 | Parvoviridae/Chapparvovirus | Mouse kidney parvovirus | YP_009553675.1 | 4603 | 12562 | 575.8 | 64.83% |
| MT138288 | xftots60par1nc | OtistardaFe060 | Parvoviridae/Chapparvovirus | Chestnut teal chaphamaparvovirus 3 | QMI57837 | 4224 | 172 | 8.6 | 49.04% |
| MT138304 | swa134par2 | swallow134 | Parvoviridae/Chapparvovirus | Psittacara leucophthalmus chapparvovirus | QEJ80805 | 4697 | 2963 | 133.1 | 51.07% |
| MT138306 | rtr167par1 | Rufous-tailedRobin167 | Parvoviridae/Chapparvovirus | Peafowl parvovirus 2 | QGJ83204 | 4487 | 20194 | 949.6 | 46.88% |
| MT138312 | ybb150par03 | Yellow-browedBunting150 | Parvoviridae/Chapparvovirus | Parvoviridae sp. | AUW34315 | 4900 | 9344 | 402.4 | 48.43% |
| MT138316 | thr146par1 | thrush146 | Parvoviridae/Chapparvovirus | Peafowl parvovirus 2 | QGJ83204 | 4284 | 2128 | 104.8 | 46.77% |
| MT138322 | zftegr01par2 | Egret01 | Parvoviridae/Chapparvovirus | Peafowl parvovirus 2 | QGJ83204 | 4560 | 2180 | 101.6 | 48.41% |
| MT138323 | xftpec96par1 | PeacockFe096 | Parvoviridae/Chapparvovirus | Avian chapparvovirus | QKX49056 | 4415 | 208 | 9.9 | 41.49% |
| MT138335 | zftwig06par1 | WildGoose06 | Parvoviridae/Chapparvovirus | Chestnut teal chaphamaparvovirus 2 | QMI57833 | 4549 | 350 | 16.2 | 64.48% |
| MT138226 | cftbif21par1 | BirdFe021 | Parvoviridae/Dependoparvovirus | Adeno-associated virus | YP_009552823 | 4724 | 1456 | 65 | 88.58% |
| MT138227 | cftbif22par1 | BirdFe022 | Parvoviridae/Dependoparvovirus | Adeno-associated virus | YP_009552823.1 | 4359 | 663 | 33.5 | 88.43% |
| MT138232 | cfw059par1 | Chestnut-flankedWhite-eye59 | Parvoviridae/Dependoparvovirus | Muscovy duck parvovirus | ANG60949.1 | 4861 | 1234 | 53.6 | 51.16% |
| MT138242 | hbl169par1 | hornbill69 | Parvoviridae/Dependoparvovirus | Murine adeno-associated virus 1 | AWB14637.1 | 4523 | 49880 | 2326.9 | 82.87% |
| MT138243 | hbl169par2 | hornbill69 | Parvoviridae/Dependoparvovirus | Murine adeno-associated virus 2 | AWB14639.1 | 4805 | 8200 | 360.1 | 92.74% |
| MT138246 | hftbif16par1 | BirdFe016 | Parvoviridae/Dependoparvovirus | Adeno-associated virus | YP_009552823.1 | 4425 | 213 | 10.2 | 85.52% |
| MT138247 | hftbif17par1 | BirdFe017 | Parvoviridae/Dependoparvovirus | Adeno-associated virus | YP_009552823.1 | 4743 | 722 | 32.1 | 87.52% |
| MT138248 | hftbif18par1 | BirdFe018 | Parvoviridae/Dependoparvovirus | Adeno-associated virus | YP_009552823.1 | 4599 | 1931 | 88.6 | 88.58% |
| MT138249 | hftbif19par1 | BirdFe019 | Parvoviridae/Dependoparvovirus | Adeno-associated virus | YP_009552823.1 | 4597 | 3923 | 185.4 | 87.50% |
| MT138255 | ltt163par1 | long-tailedtit163 | Parvoviridae/Dependoparvovirus | Avian adeno-associated virus | QEJ80808.1 | 4515 | 188193 | 8794.8 | 69.95% |
| MT138259 | ltt164par2 | long-tailedtit164 | Parvoviridae/Dependoparvovirus | Avian adeno-associated virus | QEJ80808.1 | 6352 | 11202 | 372.1 | 69.95% |
| MT138260 | ltt164par3 | long-tailedtit164 | Parvoviridae/Dependoparvovirus | Avian adeno-associated virus | QEJ80808.1 | 5291 | 10984 | 438 | 69.95% |
| MT138261 | ltt192par1 | long-tailedTit192 | Parvoviridae/Dependoparvovirus | Avian adeno-associated virus | QEJ80808.1 | 4815 | 51573 | 2260 | 69.95% |
| MT138277 | plw155par1 | PallassLeafWarbler155 | Parvoviridae/Dependoparvovirus | Avian adeno-associated virus | QEJ80808 | 4447 | 2833 | 134.4 | 65.95% |
| MT138283 | xftbif15par1nc | Birdfe015 | Parvoviridae/Dependoparvovirus | Adeno-associated virus | YP_009552823 | 3544 | 3894 | 231.8 | 88.58% |
| MT138296 | wpk049par01 | woodpecker49 | Parvoviridae/Dependoparvovirus | Adeno-associated virus | QDH44267 | 5143 | 706634 | 28990.8 | 50.96% |
| MT138301 | swa134par4 | swallow134 | Parvoviridae/Dependoparvovirus | Adeno-associated virus | QHD57622 | 4659 | 543 | 26.3 | 59.90% |
| MT138326 | zftwig01adas1 | WildGoose01 | Parvoviridae/Dependoparvovirus | Adeno-associated virus | QHD57622 | 4589 | 436 | 20 | 61.56% |
| MT138328 | zftwig05par3 | WildGoose05 | Parvoviridae/Dependoparvovirus | Avian adeno-associated virus | QEJ80808 | 4532 | 876 | 40.8 | 68.84% |
| MN933875 | nut158shi2 | nuthatch158 | Picornavirales sp. | Hubei picorna-like virus 46 | YP_009330031.1 | 9262 | 2521 | 57.4 | 55.03% |
| MN933876 | rfb198shi2 | Red-flankedBluetail198 | Picornavirales sp. | Burke-Gilman virus | AOX15251.1 | 9810 | 116048 | 2496 | 48.87% |
| MN933877 | rob114shi1 | robin114 | Picornavirales sp. | Burke-Gilman virus | AOX15251.1 | 10012 | 77704 | 1637.6 | 46.04% |
| MN933878 | trb102shi1nc | TristramsBunting102 | Picornavirales sp. | Hubei picorna-like virus 46 | YP_009330031.1 | 9889 | 9906 | 211.4 | 53.61% |
| MN933879 | wag88shi1nc | wagtail88 | Picornavirales sp. | Sanxia water strider virus 9 | YP_009337438.1 | 9699 | 5202 | 113.2 | 48.87% |
| MN933899 | ltt163shi2 | long-tailedtit163 | Picornavirales sp. | Beihai picorna-like virus 29 | APG77923.1 | 9715 | 39282 | 888.5 | 44.40% |
| MN933900 | tit162shi1nc | tit162 | Picornavirales sp. | Providence virus | YP_003620397.1 | 6594 | 58718 | 1878.9 | 38.41% |
| MN933901 | wpk139shi03 | woodpecker139 | Picornavirales sp. | Camponotus yamaokai virus | YP_009143313.1 | 5482 | 3902 | 150.2 | 47.78% |
| MT138031 | wpk139hep01 | woodpecker139 | Picornaviridae | Hepatovirus A | ART66868 | 7699 | 56306 | 1543.1 | 43.58% |
| MT138034 | zftegr01hep1nc | Egret01 | Picornaviridae | hepatovirus D2 | ALL35264 | 6250 | 2308 | 77.9 | 37.15% |
| MT138035 | zftegr01hep2nc | Egret01 | unclassified Picornavirales | Hepatovirus A | AAQ08056 | 6535 | 1218 | 44.3 | 42.62% |
| MT138036 | zftegr01hep3nc | Egret01 | Picornaviridae | Phopivirus | YP_009164966 | 6852 | 1386 | 42.7 | 47.69% |
| MT138337 | bbr034pic | BrownBrowrockpipit34 | Picornaviridae | Duck hepatitis A virus 1 | YP_007969883.1 | 7446 | 4604 | 130.5 | 52.49% |
| MT138338 | bbr144pit1 | BrownBrowrockpipit144 | Picornaviridae | Duck hepatitis A virus 1 | YP_007969883.1 | 8520 | 66686 | 1651.5 | 47.10% |
| MT138339 | bbr144pit2 | BrownBrowrockpipit144 | Picornaviridae | Picornaviridae sp. | QCF41176.1 | 7728 | 39578 | 1080.6 | 60.08% |
| MT138340 | bpk75meg01 | Bluepeacock75 | Picornaviridae/Megrivirus | Chicken megrivirus | QOQ86113.1 | 6797 | 14902 | 465.8 | 96.92% |
| MT138341 | cftwhg01pic1 | WhitegeeseFe001 | Picornaviridae/Megrivirus | Goose megrivirus | YP_009345890.1 | 7066 | 769 | 23 | 96.15% |
| MT138342 | cftwhg03pic1 | WhitegeeseFe003 | Picornaviridae/Megrivirus | Duck megrivirus | YP_009030047.1 | 9088 | 724 | 16.8 | 98.21% |
| MT138343 | cftwhg06pic2 | WhitegeeseFe006 | Picornaviridae/Aalivirus | Pacific black duck aalivirus | QMI57978.1 | 8244 | 3145 | 80.5 | 57.31% |
| MT138344 | cra070pic1 | Crane70 | Picornaviridae | Scott virus | QIS87940.1 | 7481 | 18514 | 522.2 | 37.36% |
| MT138345 | dwb062pic1nc | DuskyWarbler62 | Picornaviridae/Megrivirus | Wood duck megrivirus | QMI57989.1 | 8135 | 51548 | 1337 | 74.73% |
| MT138346 | ecw123pic1 | EasternCrownedWarbler123 | Picornaviridae | Duck hepatitis A virus 1 | YP_007969883.1 | 7490 | 41506 | 1169.3 | 48.55% |
| MT138348 | fcc172pic1 | Flycatcher172 | Picornaviridae/Oscivirus | Oscivirus A2 | ADL38962.1 | 7968 | 74110 | 1962.5 | 79.47% |
| MT138349 | gbt104pic2 | Grey-backedThrush104 | unclassified Picornavirales | Arivirus 2 | AIS73139.1 | 9444 | 17458 | 390.1 | 65.48% |
| MT138354 | muf159pic1 | MugimakiFlycatcher159 | Picornaviridae | Duck hepatitis A virus 1 | YP_007969883.1 | 7541 | 16302 | 458.4 | 50.97% |
| MT138355 | muf160pic1 | MugimakiFlycatcher160 | Picornaviridae | Duck hepatitis A virus 1 | YP_007969883.1 | 7487 | 3550 | 100 | 51.36% |
| MT138356 | nut157pic2 | nuthatch157 | Picornaviridae | Duck hepatitis A virus 3 | AEK70365.1 | 7665 | 6648 | 183 | 51.43% |
| MT138357 | phe068pic1 | pheasant68 | Picornaviridae/Megrivirus | Chicken picornavirus 5 | YP_009054902.1 | 9233 | 8132 | 185.8 | 92.73% |
| MT138358 | plw156pic4 | PallassLeafWarbler156 | Picornaviridae | Picornaviridae sp. | AUW34305.1 | 7546 | 21196 | 592.7 | 60.00% |
| MT138359 | pst217pic01 | pheasant217 | Picornaviridae/Megrivirus | Turkey hepatitis virus 2993D | YP_007974220.1 | 9500 | 6262 | 139.1 | 92.29% |
| MT138360 | rfb094pic2 | Red-flankedBluetail94 | Picornaviridae/Oscivirus | Oscivirus A2 | ADL38962.1 | 7752 | 32608 | 887.6 | 79.47% |
| MT138362 | rfb199pic1 | Red-flankedBluetail199 | Picornaviridae | Aichi virus 1 | NP_740444.1 | 7944 | 47520 | 1262.2 | 52.13% |
| MT138363 | spa143pic2 | sparrow143 | unclassified Picornavirales | Blackbird arilivirus | YP_009553600.1 | 9820 | 137902 | 2963.1 | 68.79% |
| MT138364 | spa143pic5 | sparrow143 | unclassified Picornavirales | Blackbird arilivirus | YP_009553600.1 | 10378 | 74082 | 1565.6 | 61.50% |
| MT138365 | sru051pic1 | SiberianRubythroat51 | Picornaviridae | Duck hepatitis A virus 1 | YP_007969883.1 | 7418 | 1976 | 56.2 | 48.11% |
| MT138366 | thr106pic1 | thrush106 | Picornaviridae/Megrivirus | Wood duck megrivirus | QMI57991.1 | 8914 | 19976 | 472.8 | 75.00% |
| MT138367 | thr173pic1 | thrush173 | Picornaviridae/Oscivirus | Oscivirus A2 | ADL38962.1 | 7453 | 21061 | 596.3 | 85.81% |
| MT138368 | w3chi090pic1 | chickenfe090 | Picornaviridae/Megrivirus | Chicken megrivirus | AYH53256.1 | 9092 | 2546 | 59.1 | 99.78% |
| MT138369 | w3chi091pic1 | chickenfe091 | Picornaviridae/Megrivirus | Chicken megrivirus | AYH53256.1 | 9265 | 4126 | 98.5 | 99.56% |
| MT138370 | wbp226pic1 | Whitebellygoldenpheasant226 | Picornaviridae/Megrivirus | Chicken megrivirus | QBZ68787.1 | 9506 | 12684 | 281.5 | 92.51% |
| MT138371 | wftcra73pic2 | CraneFe073 | unclassified Riboviria | Wenzhou picorna-like virus 28 | YP_009336994.1 | 8479 | 14808 | 368.5 | 40.70% |
| MT138374 | wpk049shi07 | woodpecker49 | unclassified Riboviria | Hubei tombus-like virus 40 | YP_009336559.1 | 3296 | 10210 | 653.6 | 34.64% |
| MT138375 | ybb044pic01nc | Yellow-browedBunting44 | unclassified Picornavirales | Arivirus 2 | AIS73139.1 | 9563 | 22622 | 499.1 | 56.51% |
| MT138376 | ybb117hav01 | Yellow-browedBunting117 | Picornaviridae | Pacific black duck aalivirus | QMI57978.1 | 7820 | 109068 | 2945.6 | 48.51% |
| MT138377 | ybw202pic01nc | Yellow-browedWarbler202 | Picornaviridae | Picornaviridae sp. | AUW34305.1 | 7356 | 2756 | 79.1 | 60.31% |
| MT138378 | ybw202pic02 | Yellow-browedWarbler202 | Picornaviridae | Tupaia hepatovirus A | YP_009220464.1 | 6762 | 954 | 29.8 | 47.89% |
| MT138379 | zftwig05pic3 | WildGoose05 | Picornaviridae | Picornaviridae sp. | QCF41176 | 7622 | 22956 | 635.5 | 60.08% |
| MT138380 | zftwig05pic4 | WildGoose05 | Picornaviridae | Avihepatovirus sp. | AXF38648 | 8516 | 72172 | 1788.2 | 53.38% |
| MT138381 | zftwig06pic1 | WildGoose06 | Picornaviridae/Megrivirus | Duck megrivirus | YP_009030047 | 9270 | 2220 | 50.5 | 94.57% |
| MT138393 | xftphe89pic1 | Pheasant089 | unclassified Picornavirales | Phacovirus sp. | AVN87706 | 7883 | 790 | 21.1 | 77.75% |
| MT137933 | bsk136pol01nc | BrownShrike136 | Polycipiviridae | Cacaos virus | QHA33683.1 | 10191 | 6461670 | 133785.9 | 30.03% |
| MT137935 | nut158pol1 | nuthatch158 | Polycipiviridae | Apple picorna-like virus 1 | QIC52838.1 | 10287 | 71694 | 1470.3 | 44.64% |
| MT137936 | rfb094pol1 | Red-flankedBluetail94 | Polycipiviridae | Solenopsis invicta virus 2 | AXL96895.1 | 11363 | 19616 | 364.3 | 51.27% |
| MT137937 | rfb199pol1 | Red-flankedBluetail199 | Polycipiviridae | Polycipiviridae sp. | AZL87720.1 | 11003 | 93940 | 1801.4 | 35.03% |
| MT137938 | rob114pol1 | robin114 | Polycipiviridae | Solenopsis invicta virus 2 | AXL96895 | 11290 | 13432 | 251 | 51.22% |
| MT137939 | rtr167pol1 | Rufous-tailedRobin167 | Polycipiviridae | Solenopsis invicta virus 2 | AXL96895 | 11575 | 17458 | 318.2 | 51.22% |
| MT137940 | rtr168pol1 | Rufous-tailedRobin168 | Polycipiviridae | Solenopsis invicta virus 2 | AXL96895 | 11328 | 21634 | 404.5 | 51.22% |
| MT137934 | coa196pol1 | coaltit196 | Polycipiviridae sp. | Myrmica scabrinodis virus 1 | YP_009407949.1 | 11342 | 10240 | 190.5 | 97.46% |
| MT138119 | bls219ret1 | blackswan219 | Retroviridae | Endogenous retrovirus group K member 18 | OPJ83304 | 6890 | 4796 | 146.9 | 64.20% |
| MT138120 | cat223ret1 | cabotstragopan223 | Retroviridae | Reticuloendotheliosis virus | AIG99439 | 8210 | 108 | 2.8 | 99.91% |
| MT138121 | gps222ret1 | goldenpheasant222 | Retroviridae | Reticuloendotheliosis virus | AIG99439 | 7828 | 172 | 4.6 | 99.91% |
| MN928918 | blp210cre2 | Bluepeacock210 | Smacoviridae | Human associated porprismacovirus | QBP37047 | 2549 | 4534 | 375.3 | 64.68% |
| MN928919 | bpk075sma01 | Bluepeacock75 | Smacoviridae | Chicken smacovirus mg4_964 | QIR82267 | 2423 | 1998 | 174 | 79.83% |
| MT138076 | w3chi090cir1 | chickenfe090 | Smacoviridae | Lynx rufus smacovirus 1 | QDH43738 | 2619 | 283 | 22.8 | 65.25% |
| MT138077 | w3chi091cir1 | chickenfe091 | Smacoviridae | Chicken smacovirus mg4_881 | QIR82263 | 2601 | 1707 | 138.5 | 73.90% |
| MT138078 | w3chi091cir2 | chickenfe091 | Smacoviridae | Chicken smacovirus mg2_55 | QIR82261 | 2474 | 703 | 65.4 | 79.42% |
| MT138079 | w3chi091cir3 | chickenfe091 | Smacoviridae | Lynx rufus smacovirus 1 | QDH43738 | 2587 | 181 | 14.8 | 65.25% |
| MT138081 | w3chi091cir5 | chickenfe091 | Smacoviridae | CRESS virus sp. | QCC72677 | 2475 | 709 | 60.4 | 65.77% |
| MT138084 | wbp226sma1 | Whitebellygoldenpheasant226 | Smacoviridae | Human associated porprismacovirus | QBP37047 | 2933 | 1218 | 87.6 | 63.89% |
| MT138085 | wbp226sma2 | Whitebellygoldenpheasant226 | Smacoviridae | Chicken virus mg5_1345 | QIR82213 | 2701 | 9282 | 725.1 | 97.30% |
| MT138086 | wbp226sma3 | Whitebellygoldenpheasant226 | Smacoviridae | Chicken virus mg7_59 | QIR82217 | 2687 | 5284 | 414.9 | 95.37% |
| MT138087 | wbp226sma4 | Whitebellygoldenpheasant226 | Smacoviridae | Rat stool-associated circular ssDNA virus | AKV57235 | 2594 | 1438 | 117 | 52.33% |
| MT138088 | wbp226sma5 | Whitebellygoldenpheasant226 | Smacoviridae | Porcine associated porprismacovirus | QBP37151 | 3691 | 6074 | 345.2 | 81.77% |
| MT138161 | ytb135shi04 | Yellow-throatedBunting135 | Solemo-like virus | Plasmopara viticola lesion associated sobemo-like 1 | QHD64767 | 3238 | 38848 | 2531.5 | 50.45% |
| MT138162 | bsk136shi03 | BrownShrike136 | Solemo-like virus | Hubei mosquito virus 2 | YP_009337877 | 2802 | 1566 | 117.9 | 65.71% |
| MT138163 | bsk136shi07 | BrownShrike136 | Solemo-like virus | Hubei sobemo-like virus 7 | YP_009330005 | 2846 | 9568 | 709.4 | 54.84% |
| MT138164 | bsk136shi09 | BrownShrike136 | Solemo-like virus | Hubei sobemo-like virus 45 | YP_009330092 | 2768 | 588 | 44.8 | 80.18% |
| MT138165 | bsk136shi11 | BrownShrike136 | Solemo-like virus | Jeffords solemo-like virus | QIJ70118 | 2991 | 10616 | 748.9 | 64.29% |
| MT138166 | bsk136shi18 | BrownShrike136 | Solemo-like virus | Medway virus | AWA82252 | 2670 | 26168 | 2068 | 58.85% |
| MT138167 | ybw116shi01 | Yellow-browedWarbler116 | Solemo-like virus | Hubei sobemo-like virus 49 | YP_009330082 | 3712 | 27240 | 1548.4 | 77.84% |
| MT138168 | ybw132shi03 | Yellow-browedWarbler132 | Solemo-like virus | Hubei sobemo-like virus 49 | YP_009330082 | 2816 | 856 | 66.5 | 79.78% |
| MN933865 | ecw123shi2nc | EasternCrownedWarbler123 | Totiviridae sp. | Sanxia water strider virus 8 | YP_009337665.1 | 9104 | 173464 | 4020.3 | 63.04% |
| MN933866 | fcc172shi1 | Flycatcher172 | Totiviridae sp. | Hubei picorna-like virus 41 | YP_009337750.1 | 9356 | 203224 | 4583.2 | 60.85% |
| MN933867 | ltt192shi2 | long-tailedTit192 | Totiviridae sp. | Harmonia axyridis virus 1 | QFR15912.1 | 10106 | 13969 | 291.7 | 46.67% |
| MN933868 | plw155shi1 | PallassLeafWarbler155 | Totiviridae sp. | Harmonia axyridis virus 1 | QFR15912.1 | 8849 | 10259 | 244.6 | 58.64% |
| MN933869 | rfb200shi3 | Red-flankedBluetail200 | Totiviridae sp. | Hubei picorna-like virus 41 | YP_009337750.1 | 9169 | 43094 | 998.7 | 60.85% |
| MN933870 | trb102shi2nc | TristramsBunting102 | Totiviridae sp. | Harmonia axyridis virus 1 | QFR15912.1 | 8981 | 5418 | 127.3 | 58.64% |
| MN933871 | wag54shi1nc | wagtail54 | Totiviridae sp. | Hubei picorna-like virus 40 | YP_009336539.1 | 9508 | 16148 | 358.4 | 52.65% |
| MN933872 | war203shi3 | warbler203 | Totiviridae sp. | Harmonia axyridis virus 1 | QFR15912.1 | 11790 | 638486 | 11426.7 | 60.00% |
| MN933873 | war204shi1nc | warbler204 | Totiviridae sp. | Hubei picorna-like virus 41 | YP_009337750.1 | 8705 | 6590 | 159.7 | 64.44% |
| MT138169 | ltt164upi1 | long-tailedtit164 | Totiviridae sp. | Robinvale bee virus 5 | AWK77873 | 9353 | 18896 | 429.7 | 96.23% |
| MT138170 | brb028tot1 | Brambling28 | Totiviridae sp. | Spissistilus festinus virus 1 | YP_003800001 | 8457 | 10876 | 271.4 | 34.38% |
| MT138171 | bsk136shi15nc | BrownShrike136 | Totiviridae sp. | Hubei picorna-like virus 41 | YP_009337750 | 8856 | 71846 | 1711.8 | 64.07% |
| MT138172 | bsk136shi17 | BrownShrike136 | Totiviridae sp. | Spissistilus festinus virus 1 | YP_003800001 | 6955 | 5094 | 154.5 | 43.83% |
| MT138173 | wag88rna1 | wagtail88 | Totiviridae sp. | Robinvale bee virus 5 | AWK77873.1 | 9123 | 23598 | 545.8 | 73.38% |
| MT138174 | zftfla02rna1 | Flamingo02 | Totiviridae sp. | Shahe picorna-like virus 13 | YP_009336810.1 | 9036 | 2988 | 69.8 | 100.00% |
| MT138175 | dwb110rvt2nc | DuskyWarbler110 | Totiviridae sp. | Harmonia axyridis virus 1 | QFR15912.1 | 8333 | 3092 | 78.3 | 79.32% |
| MT138176 | ybw202shi03nc | Yellow-browedWarbler202 | Totiviridae sp. | Sacbrood virus | AID58096.1 | 7292 | 2338 | 68.2 | 36.84% |
| MT138397 | cfe153pic01 | Chestnut-flankedWhite-eye153 | Totiviridae sp. | Sacbrood virus | AID58096 | 9519 | 4642 | 102.9 | 51.03% |
| MT138398 | cfw059pic1 | Chestnut-flankedWhite-eye59 | Totiviridae sp. | Harmonia axyridis virus 1 | QFR15912 | 8897 | 150996 | 3581 | 79.66% |
| MT138399 | wftcra73pic1 | CraneFe073 | Totiviridae sp. | Shahe picorna-like virus 13 | YP_009336810 | 8985 | 52948 | 1243.4 | 99.79% |
| MT138400 | xftoti59pic1 | OtistardaFe059 | Totiviridae sp. | Shahe picorna-like virus 13 | YP_009336810 | 8905 | 2512 | 59.5 | 99.79% |
| MN928921 | cra070cir2 | Crane70 | unclassified CRESS DNA virus | Circoviridae sp. | AXH76160 | 2482 | 506 | 43 | 64.62% |
| MN928922 | fla06cir1 | Flamingo06 | unclassified CRESS DNA virus | Circovirus-like genome DCCV-11 | YP_009259700 | 2981 | 740 | 52.4 | 83.75% |
| MN928923 | fla07cir1 | Flamingo07 | unclassified CRESS DNA virus | CRESS virus sp. | AXH74143 | 1804 | 198 | 24.3 | 38.72% |
| MN928924 | fla07cir2 | Flamingo07 | unclassified CRESS DNA virus | CRESS virus sp. | AXH75335 | 1953 | 778 | 84.1 | 39.06% |
| MN928927 | brs113cre1 | BrownShrike113 | unclassified CRESS DNA virus | Lake Sarah-associated circular virus-32 | ALE29729 | 2076 | 658 | 66.9 | 40.16% |
| MN928928 | cra070cir1 | Crane70 | unclassified CRESS DNA virus | Giant panda circovirus 1 | YP_009389456 | 2817 | 396 | 29.7 | 82.65% |
| MN928929 | dth148cir1 | duskythrush148 | unclassified CRESS DNA virus | Panicum ecklonii-associated virus | YP_009552773 | 2087 | 52738 | 5331.9 | 63.04% |
| MN928930 | fmg067cre3 | flamingo67 | unclassified CRESS DNA virus | Circoviridae sp. | AXQ66573 | 3327 | 3134 | 198.8 | 31.86% |
| MN928931 | hbl169cir1 | hornbill69 | unclassified CRESS DNA virus | Giant panda circovirus 1 | YP_009389456 | 2820 | 706 | 52.8 | 72.34% |
| MN928932 | hbl169cre1 | hornbill69 | unclassified CRESS DNA virus | CRESS virus sp. | AXH77842 | 2088 | 16948 | 1712.7 | 75.52% |
| MN928933 | hbl169cre2 | hornbill69 | unclassified CRESS DNA virus | Capybara virus 28_cap1_2580 | QDJ95263 | 2158 | 1324 | 128.4 | 65.81% |
| MN928934 | brs113cir1 | BrownShrike113 | unclassified CRESS DNA virus | Bat associated circovirus | QOR29567 | 2172 | 38768 | 3766.1 | 52.59% |
| MN928935 | fla04cir1 | Flamingo04 | unclassified CRESS DNA virus | Lake Sarah-associated circular virus-10 | YP_009237516 | 2015 | 402 | 42.1 | 48.91% |
| MN928936 | fla05cir2 | Flamingo05 | unclassified CRESS DNA virus | Lake Sarah-associated circular virus-10 | YP_009237516 | 2015 | 406 | 42.5 | 48.91% |
| MN928937 | fla06cir2 | Flamingo06 | unclassified CRESS DNA virus | Lake Sarah-associated circular virus-10 | YP_009237516 | 2015 | 702 | 73.5 | 48.91% |
| MN928938 | hftoti49cir2 | OtistardaFe049 | unclassified CRESS DNA virus | Circovirus-like genome DCCV-10 | YP_009259692 | 2590 | 752 | 61.3 | 48.03% |
| MN928939 | brs113cre2 | BrownShrike113 | unclassified CRESS DNA virus | Dragonfly orbiculatusvirus | YP_009021243 | 1816 | 6342 | 736.9 | 61.01% |
| MN928940 | fla04cir2 | Flamingo04 | unclassified CRESS DNA virus | Forsythia suspensa CRESS virus | QKI28982 | 1785 | 266 | 31.4 | 57.43% |
| MN928941 | fla06cir3 | Flamingo06 | unclassified CRESS DNA virus | Forsythia suspensa CRESS virus | QKI28982 | 1785 | 712 | 85.2 | 57.43% |
| MN928942 | fmg067cir4 | flamingo67 | unclassified CRESS DNA virus | Apis mellifera virus-16 | QBX89316 | 1997 | 492 | 52 | 97.64% |
| MN928943 | fmg067cir3 | flamingo67 | unclassified CRESS DNA virus | Odonata-associated circular virus-15 | YP_009551674 | 2166 | 2060 | 200.7 | 69.48% |
| MN928944 | fmg067cre2 | flamingo67 | unclassified CRESS DNA virus | CRESS virus sp. | AXH77272 | 2475 | 35662 | 3040.3 | 44.27% |
| MN928945 | fla07cir4nc | Flamingo07 | unclassified CRESS DNA virus | Avon-Heathcote Estuary associated circular virus 17 | YP_009126922 | 1772 | 332 | 39.5 | 42.35% |
| MN928946 | bbr144cre1 | BrownBrowrockpipit144 | unclassified CRESS DNA virus | CRESS virus sp. | AWY06809 | 2669 | 1012 | 80 | 64.41% |
| MN928947 | bbr144cre2 | BrownBrowrockpipit144 | unclassified CRESS DNA virus | CRESS virus sp. | AWY06809 | 2712 | 1068 | 83.1 | 56.33% |
| MN928948 | bbr144cre3 | BrownBrowrockpipit144 | unclassified CRESS DNA virus | CRESS virus sp. | AWY06809 | 2647 | 600 | 47.8 | 52.48% |
| MN928950 | blp210cre3 | Bluepeacock210 | unclassified CRESS DNA virus | Chicken microvirus mg8_95 | QIR82393 | 4924 | 712 | 31.5 | 82.29% |
| MN928952 | fla05cir3 | Flamingo05 | unclassified CRESS DNA virus | CRESS virus sp. | AXH75267 | 1829 | 1130 | 130.4 | 42.63% |
| MN928953 | fla06cir4 | Flamingo06 | unclassified CRESS DNA virus | CRESS virus sp. | AXH75267 | 1829 | 3834 | 442.3 | 42.63% |
| MN928954 | fla07cir5 | Flamingo07 | unclassified CRESS DNA virus | CRESS virus sp. | AXH75267 | 1829 | 1364 | 157.4 | 42.63% |
| MN928956 | hftoti49cir1 | OtistardaFe049 | unclassified CRESS DNA virus | Circovirus-like genome DCCV-13 | YP_009259718 | 2665 | 676 | 53.5 | 40.00% |
| MN928957 | fla05cir1nc | Flamingo05 | unclassified CRESS DNA virus | CRESS virus sp. | AXH75335 | 1984 | 306 | 32.5 | 39.06% |
| MT138040 | wpk049cir01 | woodpecker49 | unclassified CRESS DNA virus | Bat associated circovirus | QOR29567.1 | 2172 | 8020 | 779.4 | 53.70% |
| MT138045 | xftoti59cir1 | OtistardaFe059 | unclassified CRESS DNA virus | Circovirus-like genome DCCV-10 | YP_009259692.1 | 2783 | 170 | 12.9 | 38.69% |
| MT138047 | zftegr02cir1 | Egret02 | unclassified CRESS DNA virus | uncultured virus | AUM62000.1 | 2238 | 1156 | 109 | 50.62% |
| MT138049 | zftfla01cir1 | Flamingo01 | unclassified CRESS DNA virus | CRESS virus sp. | AXH75267.1 | 1829 | 4506 | 519.8 | 42.63% |
| MT138050 | zftfla01cir2 | Flamingo01 | unclassified CRESS DNA virus | uncultured virus | AUM61990.1 | 1953 | 1680 | 181.5 | 41.53% |
| MT138051 | zftfla01cir3 | Flamingo01 | unclassified CRESS DNA virus | Circoviridae sp. | AXH77818.1 | 2296 | 410 | 37.7 | 55.17% |
| MT138052 | zftfla02cir1 | Flamingo02 | unclassified CRESS DNA virus | uncultured virus | AUM61990.1 | 1953 | 11194 | 1231.8 | 41.53% |
| MT138053 | zftfla02cir2 | Flamingo02 | unclassified CRESS DNA virus | CRESS virus sp. | AXH75267.1 | 1829 | 3090 | 356.5 | 42.63% |
| MT138054 | zftfla02cir4 | Flamingo02 | unclassified CRESS DNA virus | Circovirus-like genome DCCV-11 | YP_009259700.1 | 2981 | 984 | 69.6 | 83.75% |
| MT138055 | zftfla02cir5 | Flamingo02 | unclassified CRESS DNA virus | CRESS virus sp. | AXH75500.1 | 1962 | 312 | 33.6 | 57.82% |
| MT138056 | zftfla02cir6 | Flamingo02 | unclassified CRESS DNA virus | Lake Sarah-associated circular virus-10 | YP_009237516.1 | 2015 | 1260 | 131.9 | 48.91% |
| MT138057 | zftfla02cir7 | Flamingo02 | unclassified CRESS DNA virus | Forsythia suspensa CRESS virus | QKI28982.1 | 1785 | 710 | 83.9 | 57.43% |
| MT138058 | zftfla02cir8 | Flamingo02 | unclassified CRESS DNA virus | Circoviridae sp. | AXH77818.1 | 1993 | 396 | 41.9 | 55.17% |
| MT138059 | zftfla02cir9 | Flamingo02 | unclassified CRESS DNA virus | CRESS virus sp. Ct6pe1 | QGH73510.1 | 2132 | 200 | 19.8 | 49.35% |
| MT138060 | zftfla02cir10 | Flamingo02 | unclassified CRESS DNA virus | Avon-Heathcote Estuary associated circular virus 17 | YP_009126922.1 | 1846 | 878 | 102.3 | 42.35% |
| MT138061 | zftfla03cir1 | Flamingo03 | unclassified CRESS DNA virus | Circovirus-like genome DCCV-11 | YP_009259700.1 | 2981 | 568 | 40.2 | 83.75% |
| MT138062 | zftfla03cir2 | Flamingo03 | unclassified CRESS DNA virus | CRESS virus sp. | AXH75267 | 1829 | 2642 | 304.8 | 42.63% |
| MT138064 | zftwig07cir1 | WildGoose07 | unclassified CRESS DNA virus | Chicken stool associated circular virus 1 | YP_009551346 | 3038 | 6046 | 419.9 | 80.00% |
| MT138065 | zfwcb08cir1 | ftyang08 | unclassified CRESS DNA virus | uncultured virus | AOV86329 | 2228 | 649 | 61.5 | 63.35% |
| MT138067 | zfwcb10cir1 | ftyang10 | unclassified CRESS DNA virus | uncultured virus | AOV86329 | 2228 | 394 | 37.3 | 63.35% |
| MT138069 | rtr167cir1 | Rufous-tailedRobin167 | unclassified CRESS DNA virus | Bat associated circovirus | QOR29567 | 2172 | 13236 | 1287.9 | 52.59% |
| MT138070 | sto073CRE1 | stork73 | unclassified CRESS DNA virus | Giardia intestinalis ATCC 50581 | EES99438 | 2135 | 11880 | 1174.1 | 45.37% |
| MT138071 | thr095cir1nc | thrush95 | unclassified CRESS DNA virus | Bat associated circovirus | QOR29567 | 2429 | 730 | 63.4 | 52.59% |
| MT138072 | thr146cir1 | thrush146 | unclassified CRESS DNA virus | Odonata-associated circular virus-19 | YP_009551677 | 1803 | 3898 | 456.2 | 44.40% |
| MT138073 | tou80cre1 | toucan80 | unclassified CRESS DNA virus | CRESS virus sp. | AXF52253 | 4944 | 7746 | 330.6 | 48.26% |
| MT138074 | tou80cre2 | toucan80 | unclassified CRESS DNA virus | CRESS virus sp. | AXH75182 | 4867 | 10726 | 465 | 46.82% |
| MT138075 | tou80cre3 | toucan80 | unclassified CRESS DNA virus | Crucivirus-195 | QMW68702 | 2914 | 2914 | 211 | 55.80% |
| MT138080 | w3chi091cir4 | chickenfe091 | unclassified CRESS DNA virus | Circoviridae sp | AYP28964 | 2873 | 81 | 5.9 | 95.94% |
| MT138082 | war204cre1 | warbler204 | unclassified CRESS DNA virus | CRESS virus sp. | AWY06809 | 2664 | 6172 | 487.9 | 52.48% |
| MT138094 | wftcra74cir1 | CraneFe074 | unclassified CRESS DNA virus | Circoviridae sp | AXH75398 | 1676 | 140 | 17.6 | 30.50% |
| MT138095 | wftcra75cir1nc | CraneFe075 | unclassified CRESS DNA virus | Tundra vole stool-associated circular virus | QIK03923 | 2925 | 350 | 25.2 | 35.32% |
| MN918661 | dwb184shi1 | duskywarbler184 | unclassified Picornavirales | Wuhan spider virus 5 | YP_009345015.1 | 10662 | 39278 | 777.3 | 51.48% |
| MN918662 | hbl169shi1 | hornbill69 | unclassified Picornavirales | Wenzhou picorna-like virus 28 | YP_009336994.1 | 9058 | 1758 | 41 | 39.85% |
| MN918663 | ltt163shi1 | long-tailedtit163 | unclassified Picornavirales | Hubei picorna-like virus 14 | YP_009337313.1 | 8748 | 4699 | 113.3 | 61.78% |
| MN918664 | ltt163shi4 | long-tailedtit163 | unclassified Picornavirales | Wuhan arthropod virus 3 | YP_009342254.1 | 9148 | 12603 | 290.7 | 29.24% |
| MN918665 | ltt164shi1 | long-tailedtit164 | unclassified Picornavirales | Hubei picorna-like virus 15 | YP_009336540.1 | 9405 | 7301 | 163.8 | 91.33% |
| MN918666 | mag166shi1nc | magpie166 | unclassified Picornavirales | Moyer virus | AOC55061.1 | 10001 | 1237 | 26.1 | 57.37% |
| MN918667 | muf159shi1 | MugimakiFlycatcher159 | unclassified Picornavirales | Wuhan spider virus 5 | YP_009345015.1 | 11704 | 25971 | 487.5 | 51.08% |
| MN918668 | muf160shi2 | MugimakiFlycatcher160 | unclassified Picornavirales | Rolda virus | AOY34458.1 | 9093 | 165293 | 3835.6 | 45.45% |
| MN918669 | muf160shi3 | MugimakiFlycatcher160 | unclassified Picornavirales | Hubei picorna-like virus 75 | YP_009336987.1 | 9303 | 1975 | 44.8 | 82.15% |
| MN918670 | nut158shi1 | nuthatch158 | unclassified Picornavirales | Rolda virus | AOY34458.1 | 8946 | 172995 | 4080.3 | 39.56% |
| MN918671 | nut158shi3nc | nuthatch158 | unclassified Picornavirales | Hubei picorna-like virus 43 | YP_009337372.1 | 9065 | 5820 | 135.5 | 54.88% |
| MN918672 | plw155shi3 | PallassLeafWarbler155 | unclassified Picornavirales | Sanxia water strider virus 17 | YP_009337232.1 | 9415 | 59449 | 1332.3 | 47.80% |
| MN918673 | plw156shi1 | PallassLeafWarbler156 | unclassified Picornavirales | Wuhan spider virus 5 | YP_009345015.1 | 11611 | 11228 | 204 | 51.08% |
| MN918674 | rfb092shi1 | Red-flankedBluetail92 | unclassified Picornavirales | Wuhan spider virus 5 | YP_009345015.1 | 10858 | 17015 | 330.6 | 34.90% |
| MN918675 | rfb093shi1 | Red-flankedBluetail93 | unclassified Picornavirales | Wuhan spider virus 5 | YP_009345015.1 | 8180 | 70201 | 1811.5 | 37.26% |
| MN918676 | rfb094shi2 | Red-flankedBluetail94 | unclassified Picornavirales | Wuhan spider virus 5 | YP_009345015.1 | 11259 | 1682 | 31.5 | 53.99% |
| MN918677 | rfb198shi1 | Red-flankedBluetail198 | unclassified Picornavirales | Hubei picorna-like virus 15 | APG77985.1 | 9260 | 9584 | 218.4 | 94.41% |
| MN918678 | rfb198shi3 | Red-flankedBluetail198 | unclassified Picornavirales | Hubei picorna-like virus 71 | YP_009337174.1 | 10914 | 69414 | 1342 | 52.14% |
| MN918679 | rfb199shi1 | Red-flankedBluetail199 | unclassified Picornavirales | Hubei picorna-like virus 71 | YP_009337174.1 | 10217 | 86438 | 1785.1 | 50.89% |
| MN918680 | rfb199shi3 | Red-flankedBluetail199 | unclassified Picornavirales | Wuhan spider virus 4 | YP_009345021.1 | 11603 | 18028 | 327.8 | 43.41% |
| MN918681 | rfb199shi4 | Red-flankedBluetail199 | unclassified Picornavirales | Hubei picorna-like virus 71 | YP_009337174.1 | 10041 | 32720 | 687.6 | 52.34% |
| MN918682 | rfb200shi2 | Red-flankedBluetail200 | unclassified Picornavirales | Wuhan spider virus 5 | YP_009345015.1 | 11140 | 23924 | 453.1 | 51.97% |
| MN918683 | rfb200shi4nc | Red-flankedBluetail200 | unclassified Picornavirales | Duwamo virus | YP_009272817.1 | 11558 | 29470 | 548 | 50.23% |
| MN918684 | stc111Shi1 | stonechat111 | unclassified Picornavirales | Wuhan spider virus 5 | YP_009345015.1 | 11338 | 4827 | 89.8 | 52.86% |
| MN918685 | tit162shi1 | tit162 | unclassified Picornavirales | Wuhan spider virus 5 | YP_009345015.1 | 9004 | 96700 | 2266.1 | 51.28% |
| MN918686 | tom099shi2 | tomtit99 | unclassified Picornavirales | Duwamo virus | YP_009272817.1 | 12041 | 18081 | 316.8 | 58.01% |
| MN918687 | tom152shi1 | tomtit152 | unclassified Picornavirales | Hubei picorna-like virus 71 | YP_009337174.1 | 9198 | 1532 | 35.1 | 48.37% |
| MN918688 | wag054shi1 | wagtail54 | unclassified Picornavirales | Wuhan spider virus 4 | YP_009345021.1 | 11486 | 2600 | 47.8 | 44.25% |
| MN918690 | wwb174shi04 | willowwarbler174 | unclassified Picornavirales | Nesidiocoris tenuis iflavirus 1 | YP_009552832.1 | 9976 | 99724 | 2109.2 | 39.67% |
| MN933874 | ltt163shi3 | long-tailedtit163 | unclassified Picornavirales | Fesavirus 1 | AII82258.1 | 8753 | 43914 | 1058.6 | 51.22% |
| MT138177 | coa131shi1 | coaltit131 | unclassified Picornavirales | Sanxia water strider virus 9 | YP_009337438.1 | 9320 | 11830 | 267.8 | 62.54% |
| MT138178 | dar170shi1 | DaurianRedstart170 | unclassified Picornavirales | Hubei picorna-like virus 46 | YP_009330031.1 | 8180 | 3344 | 86.3 | 64.81% |
| MT138179 | dar170shi2 | DaurianRedstart170 | unclassified Picornavirales | Burke-Gilman virus | AOX15251.1 | 11604 | 6904 | 126.4 | 48.87% |
| MT138180 | dwb062shi1 | DuskyWarbler62 | unclassified Picornavirales | Sanxia water strider virus 9 | YP_009337438.1 | 9006 | 3120 | 73.1 | 83.02% |
| MT138181 | ybw132shi02 | Yellow-browedWarbler132 | unclassified Picornavirales | Hubei picorna-like virus 50 | YP_009337053.1 | 11221 | 4754 | 89.4 | 80.29% |
| MT138391 | blp211pic1 | Bluepeacock211 | unclassified Picornavirales | Posavirus sp. | APQ44562 | 9102 | 83176 | 1928.2 | 40.66% |
| MT138392 | war204pic2 | warbler204 | unclassified Picornavirales | Paroligolophus agrestis posalike virus 1 | AOX15244 | 10396 | 79006 | 1603.5 | 41.43% |
| MT138403 | gbt104pic1 | Grey-backedThrush104 | unclassified Picornavirales | Fesavirus 1 | AII82258 | 9008 | 7750 | 181.5 | 57.91% |
| MT138404 | ltt163pic3 | long-tailedtit163 | unclassified Picornavirales | Bat felisavirus | AQP31140 | 9320 | 42420 | 965.2 | 59.29% |
| MT138405 | ltt163pic4 | long-tailedtit163 | unclassified Picornavirales | Bat felisavirus | AQP31140 | 9103 | 42442 | 983.8 | 59.64% |
| MN917668 | sbr121shi1 | SiberianBlueRobin121 | unclassified Riboviria | Solenopsis invicta virus 9 | QBL75897.1 | 9524 | 16214 | 359.2 | 69.54% |
| MN917676 | rfb198shi4 | Red-flankedBluetail198 | unclassified Riboviria | Wuhan spider virus 5 | YP_009345015.1 | 10113 | 70700 | 1475.1 | 32.89% |
| MN917677 | jyt032shi4 | jynxtorquilla32 | unclassified Riboviria | Wenzhou picorna-like virus 47 | APG78496.1 | 8906 | 6752 | 160 | 91.86% |
| MN917679 | nut157shi3 | nuthatch157 | unclassified Riboviria | Mayfield virus 1 | QAY29257.1 | 9599 | 9418 | 207 | 36.15% |
| MN917680 | ybb117shi01 | Yellow-browedBunting117 | unclassified Riboviria | Mayfield virus 1 | QAY29257.1 | 9625 | 32732 | 717.6 | 39.84% |
| MN917681 | gbt105shi1 | Grey-backedThrush105 | unclassified Riboviria | Hubei picorna-like virus 49 | YP_009336567.1 | 9499 | 300028 | 6664.5 | 39.02% |
| MN933880 | ltt163shi5 | long-tailedtit163 | unclassified Riboviria | Hubei picorna-like virus 61 | QIN93584.1 | 9244 | 15555 | 355.1 | 36.76% |
| MN933881 | ltt192shi1nc | long-tailedTit192 | unclassified Riboviria | Hubei tetragnatha maxillosa virus 4 | YP_009336535.1 | 8793 | 9583 | 254 | 99.26% |
| MN933882 | muf160shi4 | MugimakiFlycatcher160 | unclassified Riboviria | Murine feces-associated hepe-like virus | AWB14594.1 | 8331 | 20066 | 508.2 | 42.60% |
| MN933883 | plw155shi2 | PallassLeafWarbler155 | unclassified Riboviria | Hubei picorna-like virus 55 | AWS06670.1 | 11216 | 17039 | 320.5 | 99.65% |
| MN933884 | plw156shi3nc | PallassLeafWarbler156 | unclassified Riboviria | Hubei picorna-like virus 55 | AWS06670.1 | 9673 | 4619 | 100.8 | 88.97% |
| MN933885 | rfb198shi5 | Red-flankedBluetail198 | unclassified Riboviria | Boghill Burn virus | QAY29242.1 | 10414 | 46644 | 945.1 | 64.31% |
| MN933886 | swa134shi1 | swallow134 | unclassified Riboviria | Changjiang crawfish virus 6 | APG77989.1 | 9664 | 16630 | 363.1 | 97.15% |
| MN933887 | thr095shi2 | thrush95 | unclassified Riboviria | Beihai picorna-like virus 109 | APG78612.1 | 9792 | 44182 | 952 | 32.07% |
| MN933888 | tit162shi2nc | tit162 | unclassified Riboviria | Hubei tombus-like virus 13 | YP_009337096.1 | 6312 | 28674 | 958.5 | 67.09% |
| MN933889 | trb102shi1 | TristramsBunting102 | unclassified Riboviria | Changjiang crawfish virus 6 | APG77989.1 | 9776 | 12738 | 280.3 | 97.15% |
| MN933890 | wag054shi3 | wagtail54 | unclassified Riboviria | Changjiang crawfish virus 6 | APG77989.1 | 9583 | 1422 | 31.3 | 97.15% |
| MN933891 | war203shi2 | warbler203 | unclassified Riboviria | Changjiang crawfish virus 6 | YP_009336630.1 | 9768 | 2348 | 50.7 | 96.80% |
| MN933892 | wiw119shi1 | willowwarbler119 | unclassified Riboviria | Hubei tetragnatha maxillosa virus 4 | YP_009336535.1 | 9057 | 24348 | 567.2 | 52.08% |
| MN933893 | wpk049shi04 | woodpecker49 | unclassified Riboviria | Shuangao permutotetra-like virus 1 | APG76925.1 | 4863 | 12218 | 530.1 | 43.23% |
| MN933894 | wpk049shi05 | woodpecker49 | unclassified Riboviria | Renton virus | AOC55070.1 | 4327 | 9466 | 461.6 | 43.90% |
| MN933895 | wpk049shi06 | woodpecker49 | unclassified Riboviria | Hubei permutotetra-like virus 4 | YP_009337378.1 | 4068 | 10102 | 524 | 85.39% |
| MN933896 | wpk139shi04 | woodpecker139 | unclassified Riboviria | Serdyukov virus | QIS87976.1 | 4986 | 3226 | 136.5 | 69.74% |
| MN933897 | wpk139shi05 | woodpecker139 | unclassified Riboviria | Sanxia tombus-like virus 5 | YP_009337188.1 | 4283 | 6346 | 312.6 | 40.21% |
| MN933898 | wwb174shi05 | willowwarbler174 | unclassified Riboviria | Shuangao permutotetra-like virus 1 | APG76925.1 | 4418 | 619476 | 29585.7 | 44.39% |
| MT138122 | bsk136shi08 | BrownShrike136 | unclassified Riboviria | Hubei picorna-like virus 49 | YP_009336567 | 9356 | 10350 | 233.4 | 43.16% |
| MT138123 | cfe153shi06 | Chestnut-flankedWhite-eye153 | unclassified Riboviria | Sanxia water strider virus 9 | YP_009337438 | 9970 | 17594 | 375.6 | 24.74% |
| MT138134 | zftwig05rna2nc | WildGoose05 | unclassified Riboviria | Rhopalosiphum padi virus | AWK77928 | 9396 | 16046 | 360.3 | 64.13% |
| MT138135 | zftwig05rna3 | WildGoose05 | unclassified Riboviria | Wabat virus | YP_009272708 | 10271 | 2032 | 41.7 | 37.73% |
| MT138136 | zftwig05rna4 | WildGoose05 | unclassified Riboviria | Hubei picorna-like virus 15 | APG77985 | 8459 | 14748 | 367.9 | 97.83% |
| MT138137 | ybb117shi02nc | Yellow-browedBunting117 | unclassified Riboviria | Hubei orthoptera virus 3 | YP_009336506 | 9824 | 894 | 19.2 | 43.78% |
| MT138138 | ytb135shi01 | Yellow-throatedBunting135 | unclassified Riboviria | Wuhan spider virus 5 | YP_009345015 | 12024 | 305472 | 5360.5 | 56.83% |
| MT138140 | bfb201hbp01 | BlackfaceBunting201 | unclassified Riboviria | Hubei picorna-like virus 33 | YP_009337692 | 9222 | 109876 | 2514 | 71.63% |
| MT138142 | brb030urn1 | Brambling30 | unclassified Riboviria | Hubei picorna-like virus 73 | YP_009336623 | 9842 | 2176 | 47.5 | 49.22% |
| MT138143 | bsk136shi02 | BrownShrike136 | unclassified Riboviria | Tribolium castaneum iflavirus | AUE23905 | 8853 | 15544 | 370.5 | 51.82% |
| MT138144 | bsk136shi05 | BrownShrike136 | unclassified Riboviria | Wuhan spider virus 5 | YP_009345015 | 11362 | 6556 | 121.7 | 52.17% |
| MT138145 | bsk136shi12 | BrownShrike136 | unclassified Riboviria | Hubei narna-like virus 5 | YP_009336672 | 2460 | 5552 | 476.2 | 42.23% |
| MT138146 | bsk136shi16 | BrownShrike136 | unclassified Riboviria | Lampyris noctiluca iflavirus 1 | QBP37019 | 9628 | 14202 | 311.2 | 50.00% |
| MT138147 | zftfla02rna2 | Flamingo02 | unclassified Riboviria | Beihai tombus-like virus 16 | YP_009336606 | 4725 | 1420 | 63.4 | 33.54% |
| MT138149 | ybw202shi06 | Yellow-browedWarbler202 | unclassified Riboviria | PNG bee virus 4 | QKW94206 | 8445 | 9088 | 227.1 | 56.45% |
| MT138150 | ybw202shi07 | Yellow-browedWarbler202 | unclassified Riboviria | Tesano Aedes virus | BBN21000 | 3276 | 12414 | 799.6 | 53.57% |
| MT138151 | tou80rnanc1 | toucan80 | unclassified Riboviria | Hubei picorna-like virus 44 | YP_009336529 | 8477 | 1150 | 28.6 | 99.30% |
| MT138152 | cfe153shi02 | Chestnut-flankedWhite-eye153 | unclassified Riboviria | Vespa velutina associated ifla-like virus | QGL51726 | 9827 | 6142 | 131.9 | 63.32% |
| MT138153 | bs219upi01 | blackswan219 | unclassified Riboviria | Calhevirus-2a | BAP81885 | 10150 | 16738 | 348 | 60.07% |
| MT138154 | bs219upi03 | blackswan219 | unclassified Riboviria | Hubei tombus-like virus 40 | YP_009336561 | 4431 | 101278 | 4822.8 | 32.39% |
| MT138155 | ybb150shi01 | Yellow-browedBunting150 | unclassified Riboviria | Insect-associated waikavirus 1 | QHB15178 | 9070 | 21092 | 497.6 | 39.27% |
| MT138156 | bsk136shi13 | BrownShrike136 | unclassified Riboviria | Beihai noda-like virus 6 | APG76165 | 4297 | 2914 | 143.1 | 44.13% |
| MT138158 | ybw202shi02 | Yellow-browedWarbler202 | unclassified Riboviria | Ek Balam virus | AYW01753 | 5430 | 366 | 14.2 | 83.14% |
| MT138160 | bpk205shi03 | Bluepeacock205 | unclassified Riboviria | Ketchum virus | QIS88001 | 3736 | 6342 | 358.2 | 31.59% |
| MT138182 | zftwig05rna5 | WildGoose05 | unclassified Riboviria | Hubei picorna-like virus 52 | YP_009337118.1 | 6750 | 630 | 19.7 | 62.03% |
| MT138183 | wpk049rna01 | woodpecker49 | unclassified Riboviria | HVAC-associated RNA virus 1 | AVD69111.1 | 10818 | 1808930 | 35282.3 | 85.04% |
| MT138184 | ybb150shi02 | Yellow-browedBunting150 | unclassified Riboviria | Shuangao permutotetra-like virus 1 | APG76925.1 | 4286 | 1104 | 54.3 | 43.23% |
| MT138185 | ytb135shi02 | Yellow-throatedBunting135 | unclassified Riboviria | Serdyukov virus | QIS87976.1 | 3862 | 18656 | 1019.3 | 68.89% |
| MT138186 | ytb135shi03 | Yellow-throatedBunting135 | unclassified Riboviria | Hubei permutotetra-like virus 5 | YP_009337742.1 | 5119 | 6822 | 285.6 | 43.33% |
| MT138187 | yrf108shi01 | Yellow-rumpedFlycatcher108 | unclassified Riboviria | Changjiang crawfish virus 6 | APG78044.1 | 9738 | 21940 | 475.4 | 97.15% |
| MT138188 | brb141rna1 | Brambling141 | unclassified Riboviria | Robinvale bee virus 3 | AWK77878.1 | 8716 | 96154 | 2327.7 | 65.37% |
| MT138189 | bsk136shi01 | BrownShrike136 | unclassified Riboviria | Hubei permutotetra-like virus 9 | YP_009337778.1 | 5017 | 18286 | 769.1 | 45.64% |
| MT138190 | bsk136shi04 | BrownShrike136 | unclassified Riboviria | Serdyukov virus | QIS87976.1 | 4662 | 1774 | 80.3 | 68.37% |
| MT138191 | bsk136shi06 | BrownShrike136 | unclassified Riboviria | Shuangao permutotetra-like virus 1 | APG76925.1 | 4683 | 49074 | 2211.1 | 38.21% |
| MT138192 | bpk205shi01 | Bluepeacock205 | unclassified Riboviria | Forsythia suspensa tombusvirus | QKE44134.1 | 3889 | 44398 | 2408.8 | 59.32% |
| MT138193 | bpk205shi02 | Bluepeacock205 | unclassified Riboviria | Forsythia suspensa tombusvirus | QKE44134.1 | 3493 | 5212 | 314.8 | 56.13% |
| MT138195 | ybw115shi01 | Yellow-browedWarbler115 | unclassified Riboviria | Changjiang crawfish virus 6 | YP_009336630.1 | 4559 | 17780 | 822.9 | 94.68% |
| MT138196 | ybw115shi02nc | Yellow-browedWarbler115 | unclassified Riboviria | Hubei tetragnatha maxillosa virus 4 | YP_009336535.1 | 8009 | 556 | 15 | 56.69% |
| MT138198 | ybw132shi01 | Yellow-browedWarbler132 | unclassified Riboviria | Changjiang crawfish virus 6 | APG77989.1 | 9694 | 14094 | 306.8 | 95.36% |
| MT138199 | ybw202shi01 | Yellow-browedWarbler202 | unclassified Riboviria | Thrips tabaci associated picorna-like virus 1 | QNM37809.1 | 8278 | 7274 | 185.4 | 33.33% |
| MT138200 | ybw202shi05nc | Yellow-browedWarbler202 | unclassified Riboviria | Iflaviridae sp. | QKN89066.1 | 9589 | 2906 | 63.9 | 53.11% |
| MT138201 | plw155urn1 | PallassLeafWarbler155 | unclassified Riboviria | Sitobion miscanthi virus 1 | QCI31816.1 | 9660 | 13835 | 302.2 | 99.75% |
| MT138202 | rob180rna1 | robin180 | unclassified Riboviria | HVAC-associated RNA virus 1 | AVD69111.1 | 10914 | 152346 | 2945.3 | 82.04% |
| MT138203 | cfe153shi01 | Chestnut-flankedWhite-eye153 | unclassified Riboviria | Thrips tabaci associated picorna-like virus 1 | QNM37809.1 | 10073 | 1247686 | 26135.4 | 33.33% |
| MT138204 | cfe153shi03nc | Chestnut-flankedWhite-eye153 | unclassified Riboviria | Hubei picorna-like virus 48 | YP_009337018.1 | 5612 | 2230 | 83.8 | 69.75% |
| MT138205 | cfe153shi05 | Chestnut-flankedWhite-eye153 | unclassified Riboviria | Shuangao permutotetra-like virus 1 | APG76925.1 | 4944 | 414430 | 17687 | 43.23% |
| MT138382 | plw156pic1 | PallassLeafWarbler156 | unclassified Riboviria | Bat badicivirus 1 | YP_009345910 | 8238 | 2799 | 71.7 | 62.24% |
| MT138383 | rob114shi2 | robin114 | unclassified Riboviria | Wuhan spider virus 5 | YP_009345015 | 11737 | 115766 | 2081.2 | 60.14% |
| MT138385 | wftcra75pic2 | CraneFe075 | unclassified Riboviria | Hubei picorna-like virus 35 | YP_009337666 | 9165 | 7026 | 161.8 | 52.57% |
| MT138386 | wiw119pic1 | willowwarbler119 | unclassified Riboviria | Bat badicivirus 1 | YP_009345910 | 8419 | 59264 | 1465.4 | 62.24% |
| MT138387 | ytb089pic01 | Yellow-throatedBunting89 | unclassified Riboviria | Bat badicivirus 1 | YP_009345910 | 8317 | 8544 | 216.8 | 62.24% |
| MT138388 | ytb135pic02 | Yellow-throatedBunting135 | unclassified Riboviria | Scaphoideus titanus iflavirus 2 | QIJ56911 | 10572 | 11048 | 220.5 | 70.56% |
| MT138389 | ytb135pic03 | Yellow-throatedBunting135 | unclassified Riboviria | Nilaparvata lugens honeydew virus-3 | YP_008130310 | 8650 | 7144 | 174.3 | 55.12% |
| MT138390 | ytb135pic04nc | Yellow-throatedBunting135 | unclassified Riboviria | Hubei tick virus 3 | YP_009336533 | 8780 | 2930 | 70.4 | 51.25% |
| MT138394 | zftwig05pic1 | WildGoose05 | unclassified Riboviria | Paroligolophus agrestis posalike virus 1 | AOX15244 | 8312 | 842 | 21.4 | 51.09% |
| MT138395 | zftwig05pic5 | WildGoose05 | unclassified Riboviria | Paroligolophus agrestis posalike virus 1 | AOX15244 | 9260 | 90710 | 2066.9 | 50.11% |
| MT138396 | zftwig05pos1 | WildGoose05 | unclassified Riboviria | Paroligolophus agrestis posalike virus 1 | AOX15244 | 10752 | 48900 | 959.6 | 48.21% |
| MT138406 | dth148pic1 | duskythrush148 | unclassified Riboviria | Robinvale bee virus 3 | AWK77878 | 8031 | 10838 | 287.2 | 65.12% |
| MT138407 | gbt105pic1nc | Grey-backedThrush105 | unclassified Riboviria | Lycopersicon esculentum picorna-like virus | QKK82959 | 7919 | 3898 | 103.9 | 61.21% |
| MT138408 | nut157pic1nc | nuthatch157 | unclassified Riboviria | Lycopersicon esculentum picorna-like virus | QKK82959 | 8851 | 3263 | 77.8 | 74.10% |
| MT138409 | rob114pic1 | robin114 | unclassified Riboviria | Lycopersicon esculentum picorna-like virus | QKK82959 | 9266 | 14542 | 331.1 | 72.36% |
| MT138410 | rtr167pic3 | Rufous-tailedRobin167 | unclassified Riboviria | Lycopersicon esculentum picorna-like virus | QKK82959 | 8923 | 13026 | 308 | 60.50% |
| MT138411 | sto073pic1 | stork73 | unclassified Riboviria | Robinvale bee virus 3 | AWK77878 | 7907 | 30560 | 815.5 | 92.17% |
| MT138412 | thr095pic1nc | thrush95 | unclassified Riboviria | Washington bat picornavirus | YP_009272812 | 8170 | 14904 | 384.9 | 59.22% |
| MT138413 | war203pic1 | warbler203 | unclassified Riboviria | Washington bat picornavirus | YP_009272812 | 9261 | 68450 | 1559.5 | 55.01% |
| MT138414 | ytb135pic01 | Yellow-throatedBunting135 | unclassified Riboviria | Thrips tabaci associated picorna-like virus 1 | QNM37809 | 7234 | 21784 | 635.4 | 50.00% |
| MT138415 | zftegr01pic1nc | Egret01 | unclassified Riboviria | Hubei picorna-like virus 52 | YP_009337118 | 10534 | 5924 | 119.2 | 32.01% |
| MT138420 | zftwig05pic2nc | WildGoose05 | unclassified Riboviria | Insect-associated waikavirus 1 | QHB15178 | 8200 | 27220 | 700.4 | 38.75% |
| MT138206 | cra070shi2 | Crane70 | Weivirus-like virus sp. | Hubei picorna-like virus 20 | YP_009337055.1 | 8321 | 33528 | 850.2 | 80.20% |
| MT138207 | cra070shi3 | Crane70 | Weivirus-like virus sp. | Hubei picorna-like virus 20 | YP_009337055.1 | 8426 | 46846 | 1198.5 | 80.45% |
| MN917675 | swa066shi2 | swan66 | (Weivirus-like)unclassified Riboviria | Changjiang crawfish virus 3 | YP_009336760.1 | 8230 | 12868 | 329.9 | 99.38% |
| MT138416 | plw156pic2 | PallassLeafWarbler156 | (Weivirus-like)unclassified Riboviria | Human blood-associated dicistrovirus | AWK23470 | 9252 | 2711 | 670.8 | 73.51% |
| MT138417 | war204pic4nc | warbler204 | (Weivirus-like)unclassified Riboviria | Bundaberg bee virus 2 | AWK77852 | 9716 | 11306 | 245.5 | 73.68% |
| MT138418 | zftfla02pic2 | Flamingo02 | (Weivirus-like)unclassified Riboviria | Shahe picorna-like virus 9 | YP_009336753 | 8530 | 1436 | 36 | 58.72% |
| MT138419 | zftfla02pic3 | Flamingo02 | (Weivirus-like)unclassified Riboviria | Wenzhou picorna-like virus 40 | YP_009336862 | 4834 | 1684 | 73.5 | 52.87% |
