# Supplementary material for: Virome in the cloaca of wild and breeding birds revealed a diversity of significant viruses
Source: Microbiome. 2022 Apr 12;10:60. doi: 10.1186/s40168-022-01246-7 (PMC9001828; doi:10.1186/s40168-022-01246-7)
Supplement: Supplementary file 17 — Additional file 16: Supplementary Table 5. Primers used in the PCR confirmation of the 10 unclassified CRESS DNA viruses and 10 unclassified Riboviria genomes in the original sample pools of birds’ cloacal swabs. [file 40168_2022_1246_MOESM16_ESM.docx]

**Supplementary Table 5. Primers used in the PCR conformation of the 10 unclassified CRESS DNA virus and 10 unclassified Riboviria in the original sample pools of birds’ cloacal swabs**.

| GenBank no. | Virus name | Primers | | Fragment size(bp) | Target region | Annealing temperature (°C) |
| --- | --- | --- | --- | --- | --- | --- |
|  |  | Forward primer(5'-3') | Reverse primer(5'-3') |  |  |  |
| MN933887 | unclassified Riboviria | CAACCAATCCTACACGCCCT | TCTCCACATCCAACGGAAGC | 461 | RdRp | 55 |
| MN933892 | unclassified Riboviria | TGGCATTGCAGACAACATGC | GGAAGCTTCCGTCAGACACA | 542 | RdRp | 55 |
| MT138137 | unclassified Riboviria | TGGCGCTCTTCTTACTGCAA | CTTCCCAAGAGGCTGCTTCA | 461 | RdRp | 55 |
| MT138185 | unclassified Riboviria | GATCATGACGGCGACTGGAT | TCAATGCTCACTGCCAGAGG | 374 | RdRp | 57 |
| MT138191 | unclassified Riboviria | CGAGTTGCTTGTCTCCCAGT | ACCTGAGAATTTCGCTCGCA | 361 | RdRp | 55 |
| MT138199 | unclassified Riboviria | ACACCCTGGTACGTTTGGAC | AGTGATGCTGTAGGCTGCTG | 486 | RdRp | 55 |
| MT138205 | unclassified Riboviria | TGTCGGAATTGCTGGCAGAT | ATCGTCTCCATAGGCGAAG | 455 | RdRp | 52 |
| MT138390 | unclassified Riboviria | ACTTTGGTCAGTGGCAAGCT | CAGCCATTCACGCATTCTCG | 272 | RdRp | 55 |
| MT138407 | unclassified Riboviria | GCATATGCGAGCTGAACTGC | AATCCGGGTGCTTCCACTC | 352 | RdRp | 55 |
| MT138420 | unclassified Riboviria | ACGTCGCCTGGTCTATGCT | AGCGACTATACAGCTGCGTC | 418 | RdRp | 55 |
| MN928923 | unclassified CRESS DNA virus | CGCGACTCTTTCCAGAACCT | CATTGGCAGGGGTACGTACA | 376 | Rep | 57 |
| MN928929 | unclassified CRESS DNA virus | AATTTGGTGAGCCGCCTGTA | CCTCGACAACAGATGGCTCA | 480 | Rep | 55 |
| MN928933 | unclassified CRESS DNA virus | ACTGCTTCCTGGTTGTAGCC | CAAGGAGGTGGGAGAGCAAG | 474 | Rep | 55 |
| MN928938 | unclassified CRESS DNA virus | ACCAACCAAGAACACCCGTT | ATGATCTCGCGCATGTTTGC | 381 | Rep | 55 |
| MN928941 | unclassified CRESS DNA virus | CCCTTACCTCCTTCGCGATC | GGACTCCTCATCTCCAGGGT | 451 | Rep | 55 |
| MN928945 | unclassified CRESS DNA virus | CTTCTCGACGGTCCAGTAGC | GTGAGGCCTATGGAGAACGG | 450 | Rep | 55 |
| MN928948 | unclassified CRESS DNA virus | GTTCGTCGCAGTAACGCATC | TCTGGCTCTGGCAAGACTTG | 320 | Rep | 55 |
| MT138040 | unclassified CRESS DNA virus | ATTGAATGACCGGGCACACT | TTGCCACTACCACTCTTGCC | 300 | Rep | 55 |
| MT138051 | unclassified CRESS DNA virus | CCTGTTCCTGTTGGTCCGAA | AAGGAACACAACTCGACGCT | 376 | Rep | 55 |
| MT138070 | unclassified CRESS DNA virus | CAGTTAAGCTCATGTCGCGC | TGTACACTGAGAGCCACTTG | 463 | Rep | 55 |
